# Standardizing Cell Ontology terms for cross-study integration in the female reproductive tract

**DOI:** 10.1126/sciadv.aef2358

**Published:** 2026-10-09

**Authors:** Huan Ting Ong, Diane C. Saunders, Caroline Eastwood, Aleix Puig-Barbe, Alison Kochersberger, Anna Galligos, Bailey Marshall, Benjamin K. Johnson, Caroline E. Kratka, Chen Jin, Daniela Betancur, Gabriela Rapozo Guimarães, Jordan H. Machlin, Jose V. V. Isola, Katelyn M. Adam, Osmaray Morales-Casanova, Svetlana Djirackor, Taylor Schissel, Valentina Lorenzi, Xifan Wang, Xingyu Shen, Bikem Soygur, Brian Aevermann, Eliza A. Gaylord, Mariko H. Foecke, Norbert K. Tavares, Ryan M. Samuel, Sophia Szady, Srinivasan Mahalingam, Subhasri Biswas, Will Liao, Bérénice A. Benayoun, Hattie Chung, Wipawee Winuthayanon, Jun Z. Li, Matteo A. Molè, Michael Angelo, Patricia Jeudin, Ronny Drapkin, Ariella Shikanov, Diana J. Laird, Francesca Duncan, Hui Shen, Jennifer L. Garrison, Mariana Boroni, Michael B. Stout, Roser Vento-Tormo, Sophia H. L. George, Yousin Suh, Jennifer L. Young, Monica M. Laronda

**Affiliations:** ^1^ Mechanobiology Institute, National University of Singapore, Singapore, Singapore.; ^2^ Stanley Manne Children’s Research Institute, Ann & Robert H. Lurie Children’s Hospital of Chicago, Chicago, IL, USA.; ^3^ Department of Pediatrics, Division of Endocrinology, Northwestern University Feinberg School of Medicine, Chicago, IL, USA.; ^4^ Wellcome Sanger Institute, Wellcome Genome Campus, Cambridge, UK.; ^5^ EMBL-European Bioinformatics Institute, Wellcome Genome Campus, Cambridge, UK.; ^6^ Buck Institute for Research on Aging, Novato, CA, USA.; ^7^ Stowers Institute for Medical Research, Kansas City, MO, USA.; ^8^ Chan Zuckerberg Initiative Biohub, Redwood City, CA, USA.; ^9^ Department of Epigenetics, Van Andel Institute, Grand Rapids, MI, USA.; ^10^ Department of Obstetrics and Gynecology, Northwestern University Feinberg School of Medicine, Chicago, IL, USA.; ^11^ Department of Obstetrics and Gynecology, Columbia University Irving Medical Center, New York, NY, USA.; ^12^ Miller School of Medicine, Medical Campus, University of Miami, Miami, FL, USA.; ^13^ Sylvester Comprehensive Cancer Center, Miller School of Medicine, Medical Campus, University of Miami, Miami, FL, USA.; ^14^ Laboratory of Bioinformatics and Computational Biology, Division of Experimental and Translational Research, Brazilian National Cancer Institute (INCA), Rio de Janeiro, Brazil.; ^15^ Department of Biomedical Engineering, University of Michigan, Ann Arbor, MI, USA.; ^16^ Cellular and Molecular Biology Program, University of Michigan, Ann Arbor, MI, USA.; ^17^ Aging & Metabolism Research Program, Oklahoma Medical Research Foundation, Oklahoma City, OK, USA.; ^18^ Nutrition College, Federal University of Pelotas, Pelotas, Brazil.; ^19^ Leonard Davis School of Gerontology, University of Southern California, Los Angeles, CA, USA.; ^20^ Center for Healthy Aging in Women, Buck Institute for Research on Aging, Novato, CA, USA.; ^21^ Eli and Edythe Broad Center for Regeneration Medicine and Stem Cell Research, University of California, San Francisco, San Francisco, CA, USA.; ^22^ Center for Reproductive Sciences, Department of Obstetrics, Gynecology and Reproductive Science, University of California, San Francisco, San Francisco, CA, USA.; ^23^ Bakar Aging Research Institute, University of California, San Francisco, San Francisco, CA, USA.; ^24^ Vitra Labs Inc., San Francisco, CA, USA.; ^25^ Department of Cellular and Molecular Pharmacology, University of California, San Francisco, CA, USA.; ^26^ Department of Medicine, Cardiovascular Research Center, Yale School of Medicine, New Haven, CT, USA.; ^27^ Department of Obstetrics, Gynecology and Women’s Health, School of Medicine, University of Missouri-Columbia, Columbia, MO, USA.; ^28^ New York Genome Center, New York, NY, USA.; ^29^ Cancer Biology Department, USC Keck School of Medicine, USC Norris Comprehensive Cancer Center, Los Angeles, CA, USA.; ^30^ Pharmacology and Pharmaceutical Sciences Department, USC Mann School of Pharmacy and Pharmacology, Los Angeles, CA, USA.; ^31^ USC Stem Cell Initiative, Los Angeles, CA, USA.; ^32^ Department of Molecular, Cellular and Developmental Biology, Yale University, New Haven, CT, USA.; ^33^ Department of Obstetrics, Gynecology and Reproductive Sciences, Yale School of Medicine, New Haven, CT, USA.; ^34^ Yale Center for Research on Aging, Yale School of Medicine, New Haven, CT, USA.; ^35^ Division of Animal Sciences, College of Agriculture, Food and Natural Resources, University of Missouri-Columbia, Columbia, MO, USA.; ^36^ Department of Molecular Genetics and Genome Sciences, College of Medicine, University of Oklahoma Health Campus, Oklahoma City, OK, USA.; ^37^ Department of Human Genetics, University of Michigan, Ann Arbor, MI, USA.; ^38^ Gilbert S. Omenn Department of Computational Medicine and Bioinformatics, University of Michigan, Ann Arbor, MI, USA.; ^39^ Institute for Stem Cell Biology and Regenerative Medicine, Stanford University, Stanford, CA, USA.; ^40^ Department of Obstetrics and Gynecology, Stanford University School of Medicine, Stanford, CA, USA.; ^41^ Dunlevie Maternal-Fetal Medicine Center for Discovery, Innovation and Impact, Stanford University School of Medicine, Stanford, CA, USA.; ^42^ Department of Pathology, Stanford University, Stanford, CA, USA.; ^43^ Immunology Program, Stanford University School of Medicine, Stanford, CA, USA.; ^44^ Cancer Biology Program, Stanford University School of Medicine, Stanford, CA, USA.; ^45^ Parker Institute for Cancer Immunotherapy, San Francisco, CA, USA.; ^46^ Penn Ovarian Cancer Research Center, Department of Obstetrics and Gynecology, University of Pennsylvania Perelman School of Medicine, Philadelphia, PA, USA.; ^47^ Basser Center for BRCA, Abramson Cancer Center, University of Pennsylvania School of Medicine, Philadelphia, PA, USA.; ^48^ Department of Obstetrics and Gynecology, University of Michigan, Ann Arbor, MI, USA.; ^49^ Helen Diller Family Comprehensive Cancer Center, University of California, San Francisco, San Francisco, CA, USA.; ^50^ Productive Health Global Consortium, Novato, CA, USA.; ^51^ Experimental Medicine Research Cluster (EMRC), Universidade Estadual de Campinas (UNICAMP), Campinas, Brazil.; ^52^ Oklahoma City Veterans Affairs Medical Center, Oklahoma City, OK, USA.; ^53^ Cambridge Stem Cell Institute, University of Cambridge, Cambridge, UK.; ^54^ Department of Genetics and Development, Columbia University Irving Medical Center, New York, NY, USA.; ^55^ Department of Biomedical Engineering, National University of Singapore, Singapore, Singapore.

## Abstract

The female reproductive tract is essential for fertility, pregnancy, and overall health, yet many of its cellular components remain poorly defined. Recent advances in single-cell and spatial transcriptomics have begun to reveal this complexity, but inconsistent naming of cell types and states has limited the ability to compare findings across studies. To address this challenge, an international group of reproductive biologists and ontology experts collaborated to harmonize annotations within Cell Ontology, focusing on the ovary, fallopian tube, and uterus. Standardized terms are proposed for major epithelial, stromal, and germ cell populations, supported by marker gene sets and anatomical linkages. This framework provides a shared reference that can be used to harmonize existing datasets and guide annotation of future studies, enabling consistent classification of oocytes, granulosa, theca, luteal, epithelial, stromal, and immune cells across tissues. By establishing a unified taxonomy, this work lays the foundation for integrating datasets, supporting cross-tissue comparisons, and advancing the understanding of reproductive biology, health, and disease.

## INTRODUCTION

The female reproductive tract (FRT) (Fig. 1) not only enables reproduction but also supports systemic health, with its dysfunction contributing to many pathologies. This has made it a robust area for scientific research, with a recent surge in public interest. However, despite the great strides that the field has made toward untangling the complex biology of reproduction, many unanswered questions remain within the FRT. A deeper understanding of the FRT at cellular resolution is critical for addressing global health concerns, including infertility, contraceptive development, reproductive toxicity, pregnancy complications, and gynecological diseases that affect millions worldwide. The emergence of single-cell and spatial transcriptomic technologies has provided a new avenue to capture fine biological details through the generation of highly detailed cellular maps. However, while such methods have greatly enhanced our ability to understand the FRT, they have been accompanied by new challenges, including the development of a standardized nomenclature for all newly and previously identified cell types, as well as cross-tissue and species comparisons. To begin addressing this need, a group of mammalian reproductive biologists actively working on FRT single-cell and spatial datasets convened to harmonize annotations and guide improvements on the representation of FRT cell types within the Cell Ontology (commonly abbreviated as CL). This review article summarizes the consensus of this meeting, with the goal of providing a shared vocabulary and structured definitions that allow cell identities to be compared, integrated, and reused across future studies and atlas efforts (*1*).

**Fig. 1.**
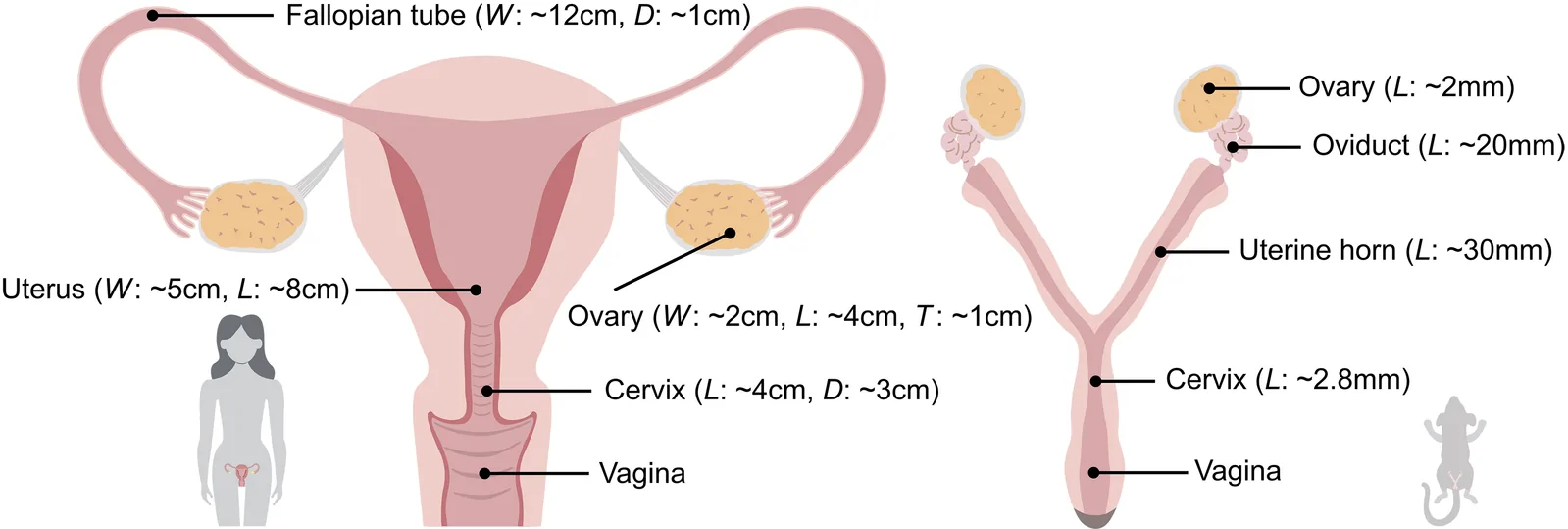
Schematic of the FRT. FRT in human (left) and mouse (right), with approximate dimensions of each anatomical compartment. Figure created by L. S. E. Kim using Adobe Illustrator (Adobe Inc., San Jose, California).

The primary function of the female reproductive system is to produce mature gametes and support embryonic and fetal development. This journey begins with an immature egg, an oocyte, established in the ovary. Each oocyte exists within a follicle composed of multiple specialized cell types tasked with preparing the oocyte for ovulation and, ultimately, fertilization. Under the guidance of hormonal cues, a single cumulus-oocyte complex (oocyte and surrounding cumulus granulosa cells) in humans or multiple cumulus oocyte complexes in poly-ovulatory mammalian species (e.g., mouse) will reach maturity and be ovulated from the ovary into the fallopian tube (FT). The FT’s ciliated walls and peristaltic contractions (*2*) mediate the mature oocyte’s transport to the uterus. If copulation has occurred and sperm has reached the FT, fertilization may take place. Upon fertilization by a single sperm, a zygote is formed. The zygote will undergo multiple mitotic divisions within the FT, and by the blastocyst stage, it will reach the hormonally primed uterus. The blastocyst embryo will attach to the lining of the uterus, termed the endometrium, where it will begin the process of uterine invasion and placentation. If fertilization does not occur, the mature egg continues its passage through the FT toward the uterus, where it degenerates ∼12 to 24 hours postovulation. Concurrently, the corpus luteum, formed from the remnants of the follicle, ceases hormonal secretions because of the absence of pregnancy signals. The resulting decline in hormonal levels initiates the shedding of the uterine lining, known as menstruation, effectively resetting the reproductive cycle for future opportunities for fertilization. This phenomenon is a delicately orchestrated process requiring the synchronization of multiple organs and many specialized cell types that are still being characterized. Yet despite this complexity, Cell Ontology has historically relied on broad, static terms that failed to capture the cellular resolution now achievable with single-cell and spatial transcriptomic approaches, a gap this manuscript directly addresses.

A major challenge in this endeavor lies in distinguishing between cell type and cell state (*3*), a distinction that has become increasingly important in the era of single-cell omics. Broadly, cell types are defined by relatively stable and heritable features, including lineage, characteristic gene expression profiles, and functional roles within tissues, whereas cell states describe more transient or context-dependent expression states cells adopted in response to physiological cues, environmental signals, or developmental timing (*1*, *3*). Historically, cells were categorized on the basis of morphology, function, location, the expression of a handful of known markers, or a mixture of all these features (*4*–*6*). For example, epithelial cells in the uterus could be classified into luminal cells, glandular cells, or ciliated cells via traditional histology. While bulk sequencing experiments failed to detect heterogeneity because of their lack of cellular resolution (*3*), single-cell and spatial technologies are now powerful enough to capture dynamic biological processes and show that cells previously categorized under the same type can exhibit substantial variability in gene expression profiles, making the boundary between cell type and cell state increasingly nuanced (*5*, *7*–*9*). In the context of the female reproductive system, these technologies can show how gene expression within a given cell type changes in response to physiological conditions, such as the hormonal fluctuations of the menstrual cycle, biological processes such as ovulation, or the adaptations required during pregnancy. As the FRT is highly dynamic and its function is driven by cycling hormonal cues, cells undergo multiple transitions in which they adopt distinct morphologies, biological functions, and gene expression profiles. The refined cell type annotations presented in this review reflect the dynamic nature of these tissues, and although these cell types are transient, they represent discrete biological functions within a cell’s trajectory and irreversible alterations to cell identity, distinguishing them from reversible cell states or developmental intermediates.

It is essential to catalog and map all cell identities, which include cell types and cell states, using datasets such as Cell Ontology (commonly abbreviated as CL) (*1*, *10*). This standardized framework provides consistent classification and legacy and evolving knowledge, enabling more accurate comparisons and interpretations across studies, as well as integration of sequencing datasets. Cell Ontology provides unique identifiers to cell types, along with associated metadata and relationships, not only between Cell Ontology terms but also with other ontologies, such as Uberon, which is a dictionary used to describe cellular localization. Formalizing biological knowledge into machine-readable definitions and axioms, ontologies (e.g., Cell Ontology) enable researchers to link disparate datasets and ensure consistent interpretation of findings across studies, thereby enhancing computational access and interoperability (*11*). Multiple efforts, such as CZ CELLxGENE (*12*), the Human BioMolecular Atlas Program (*13*), or the Human Cell Atlas (*10*, *14*), annotate all their cellular datasets to the Cell Ontology identifiers.

## TOWARD A UNIFIED NOMENCLATURE ACROSS THE FRT

A comparison of existing mouse ovary transcriptomics datasets using their published marker genes reveals overlapping cell types with differing nomenclature (fig. S1). For example, Mixed Luteal (*9*), Luteal-Parm1 (*8*), GC_Lytic (*15*), and Luteal 1 (*15*) share many marker genes indicating a common cell type, but their naming does not make this clear, making it difficult to interpret results across publications without reanalyzing or integrating the data. A unified nomenclature for the FRT is essential to enable cross-study integration and allow data from diverse sources to be compared, harmonized, and studied collectively. Accurate ontology terms provide the foundation for consistent dataset annotation and interpretation across tissues and species. To advance this goal, we propose an updated taxonomy and nomenclature for FRT cell types that can be directly applied to annotation of new single-cell and spatial transcriptomic datasets, as well as the reannotation and integration of existing studies. Because different platforms vary in their ability to recover cell populations and detect transcripts, the marker gene sets proposed here are intended as combinatorial signatures and interpreted as enriched expression rather than exclusively restricted to the indicated cell type. This framework is supported by curated, species-specific marker gene sets (Tables 1 to 3 and table S1) intended to guide reproducible cell-type assignment across platforms and analytical workflows.

**Table 1.** Ovarian cell types with Cell Ontology. Ovarian cell types with Cell Ontology (CL) IDs and marker genes in mouse and human. Marker genes represent transcriptionally differentially expressed genes from the cited literature and should be interpreted as enriched rather than exclusively restricted to the indicated cell type. Expression of individual genes across multiple cell types is expected and does not preclude their utility as part of a marker gene panel.

CL ID	CL label (cell type)	Markers
Ovary-specific		
CL:0000023	Oocyte	**Mouse**: *Gdf9*, *Kit*, *Lhx8*, *Nobox*, *Ooep*, *Oope*, *Sohlh1*, *Sohlh2*, *Zp3*
		**Human**: *ATG7*, *C6orf52*, *DPPA3*, *FIGLA*, *GDF9*, *H1-1*, *HIST1H2BK*, *KCNQ1-AS1*, *LHX8*, *OOSP2*, *PADI6*, *PDCD5*, *REC114*, *STAG3*, *TUBB8*, *UCHL1*, *WEE2*, *ZFAND2A*, *ZP3*, *ZP4*
CL:2000064	Ovarian surface epithelial cell	**Mouse**: *Epcam*, *Ildr2*, *Krt18*, *Krt19*, *Krt7*, *Lgals7*, *Upk1b*, *Upk3b*
		**Human**: *CALB2*, *CDH1*, *CLDN1*, *COBL*, *ERBB4*, *FYB2*, *LRRN4*, *MSLN*, *OVGP1*, *PAX8*, *UPK3B*
CL:4033066	Pre-granulosa cell	**Mouse**: *Foxl2*, *Kitl*, *Krt19*, *Upk3b*, *Wnt4*, *Wnt6*
		**Human**: *FOXL2*, *SOX15*, *SOX17*, *WNT6*
CL:0000501	Granulosa cell	**Mouse**: *Amh*, *Areg*, *Bex4*, *Btc*, *Cyp19a1*, *Ereg*, *Fam13a*, *Fgfr1*, *Foxl2*, *Foxp2*, *Fshr*, *Gdf7*, *Gja4*, *Grem2*, *Hsd17b1*, *Inha*, *Ivns1abp*, *Kcnq5*, *Lect1*, *Nr5a2*, *Pgr*, *Prkar2b*, *Sema5a*, *Serpine2*, *Slc18a2*, *Socs2*, *Star*, *Tuba1a*, *Wt1*
		**Human**: *AMH*, *ATPIF1*, *BEX1*, *CDH2*, *COL1A2*, *CYP11A1*, *CYP19A1*, *DCN*, *EMX2*, *FOXL2*, *FST*, *GATA4*, *GATM*, *GSTA1*, *HSD17B1*, *INHBA*, *INHBB*, *IRX3*, *IVNS1ABP*, *MIR202HG*, *PRKAR2B*, *REEP6*, *SERPINE2*, *SPINT2*, *STAR*, *TNNI3*, *WIPF3*, *WT1*
CL:0000711	Cumulus granulosa cell	**Mouse**: *Col18a1*, *Cyp19a1*, *Elobl*, *Fndc5*, *Gatm*, *Gdf9*, *H1foo*, *Kctd14*, *Khdc1b*, *Nlrp14*, *Npm2*, *Nupr1*, *Padi6*, *Uchl1*, *Wt1*, *Zbed3*
		**Human**: *FST*, *HTRA1*, *IGFBP2*, *IHH*, *INHBB*, *VCAN*
CL:4033067	Mural granulosa cell	**Mouse**: *Cited2*, *Cyp19a1*, *Ephx2*, *Grem1*, *Gsta4*, *Hsd17b1*, *Inhba*, *Mro*, *Nap1l5*, *Nppc*, *Nts*, *Rhox8*, *Slc26a7*, *Star*, *Sult1ei*, *Tom1l1*, *Tsc22d1*, *Vcan*, *X1110032F04Rik*
		**Human**: *CITED2*, *KRT18*
CL:0002095	Hilar cell	**Human**: *CALB2*, *INHA*, *INHBA*, *INHBB*, *INHBC*, *INHBE*, *MLANA*
CL:0000503	Theca cell	**Mouse**: *Aldh1a1*, *Cyp11a1*, *Dlk1*, *Lhcgr*, *Mgarp*, *Notch3*, *Ptch1*, *Srd5a1*, *Star*
		**Human**: *CYP11A1*, *CYP17A1*, *ENAH*, *LAMB1*, *LINC02476*, *MEG8*, *OSBPL1A*, *PPP3CA*, *PSD3*, *PTCH2*, *STAR*, *SULF2*
CL:4052013	Externa theca cell	**Human**: *STAT3*
CL:4052012	Interna theca cell	**Mouse**: *Apoa1*, *Cyp17a1*, *Plin2*
		**Human**: *ANPEP*, *FDX1*, *INSL3*
CL:0000175	Luteal cell	**Mouse**: *Adamts1*, *Cdkn1a*, *Cemip*, *Cyp11a1*, *Loxl2*, *Mrap*, *Mt2*, *Onecut2*, *Parm1*, *Prss35*, *Ptgfr*, *S100a6*, *Tinagl1*
CL:4052046	Early luteal cell	**Mouse**: *Lhcgr*, *Parm1*, *Prlr*, *Star*
CL:4052047	Late luteal cell	**Mouse**: *Akr1c18*, *Nupr1*, *Ptgfr*
CL:0002132	Stromal cell of the ovary	No specific marker genes established; population defined by absence of markers distinguishing pre-theca, steroidogenic stromal, fibroblast, endothelial, pericyte, smooth muscle, immune, and neural subpopulations. See text.
CL:4052010	Pre-theca cell	**Mouse**: *Enpep*, *Hhip*, *Ptch1*
		**Human**: *HHIP*, *PTCH1*
CL:4052045	Steroidogenic stromal cell of ovary	**Mouse**: *Ptch1*
Endothelial		
CL:0000115	Endothelial cell	**Human**: *CD34*, *CDH5*, *EMCN*, *FLI1*, *FLT1*, *NES*, *NUAK1*, *PALMD*, *PECAM1*, *PIK3R3*, *PODXL*
CL:0002139	Endothelial cell of vascular tree	**Human**: *APOD*, *CLDN5*, *CLEC14A*, *CTGF*, *EGFL7*, *EPAS1*, *GNG11*, *LIFR*, *MEF2C*, *RAMP2*, *RBP7*, *SLC9A3R2*, *TXNIP*, *VWF*
CL:0002138	Lymphatic endothelial cell	**Human**: *ANGPT2*, *APOD*, *FABP5*, *JMJD1C*, *LIMS1*, *LMO2*, *LOX*, *MMRN1*, *PPFIBP1*, *PTX3*, *RAB11A*, *SEMA3A*, *SMAD1*, *SPTBN1*, *TFPI*
Immune		
CL:0000236	B cell	**Mouse**: *Cd19*, *Cd79a*, *Ptprc*
CL:0000795	CD8-positive, alpha-beta regulatory T cell	**Mouse**: *Cd8b1*
CL:0000451	Dendritic cell	**Mouse**: *Clec10a*, *Clec9a*, *Fscn1*, *Itgax*
CL:0011024	Double negative T regulatory cell	**Mouse**: Absence of *Cd4*, absence of *Cd4*, *Cd3e*, *Il7r*, *Tcrgc2*, *Tmem176b*
CL:0001044	Effector CD4-positive, alpha-beta T cell	**Mouse**: *Cd28*, *Cd4*
CL:0000771	Eosinophil	**Mouse**: *S100a8*, *S100a9*
CL:0001069	Group 2 innate lymphoid cell	**Mouse**: *Il17rb*
CL:0001071	Group 3 innate lymphoid cell	**Mouse**: *Tmem176b*
CL:0000738	Leukocyte	**Mouse**: *Ptprc*
		**Human**: *C1QA*, *CCL3*, *CCL4*, *CCL5*, *CD69*, *HCST*, *IL8*, *NKG7*, *RGS1*, *TYROBP*
CL:0000235	Macrophage	**Mouse**: *Adgre1*, *Cd68*, *Csf1r*, *Itgam*, *Ly6c2*, *Mrc1*
		**Human**: *AKAP13*, *CD14*, *CD68*, *DPYD*, *FKBP5*, *FOLR2*, *KANSL1*
CL:0000814	Mature NK T cell	**Mouse**: *Ly6c2*
CL:0000576	Monocyte	**Mouse**: *Ccr2*, *Treml4*
CL:0000623	Natural killer cell	**Mouse**: *Klrb1c*, *Ly6c2*
CL:0000775	Neutrophil	**Mouse**: *S100a8*, *S100a9*
CL:0000084	T cell	**Mouse**: *Cd3e*, *Ptprc*
		**Human**: *CD3E*, *CD3G*
CL:0000864	Tissue-resident macrophage	**Human**: *LYVE1*, *MAF*, *NRP1*
Other stromal		
CL:0000057	Fibroblast	**Human**: *BNC2*, *CCL2*, *CFD*, *CXCL1*, *CXCL9*, *DCN*, *ENPP2*, *IGFBP5*, *IL24*, *LUM*, *PDGFRA*, *RAMP1*, *VIM*
CL:0000134	Mesenchymal stem cell	**Human**: *GATA2*, *NR2F1*, *TCF21*
CL:0000540	Neuron	**Human**: *CDH19*, *NRXN1*
CL:0000669	Pericyte	**Human**: *NOTCH3*, *PDGFRB*
CL:0000192	Smooth muscle cell	**Mouse**: *Myh11*
		**Human**: *ACTA2*, *CDC42EP3*, *ESR1*, *JAZF1*, *LITAF*, *LONRF2*, *NRIP1*, *PAWR*, *PLAT*, *PRLR*, *RAMP1*, *RGS5*, *TAGLN*

**Table 2.** FT cell types with Cell Ontology. FT cell types with Cell Ontology (CL) IDs and marker genes in mouse and human. Marker genes represent transcriptionally differentially expressed genes from the cited literature and should be interpreted as enriched rather than exclusively restricted to the indicated cell type. Expression of individual genes across multiple cell types is expected and does not preclude their utility as part of a marker gene panel.

CL ID	CL label (cell type)	Markers
Fallopian tube–specific		
CL:4052070	Unclassified fallopian tube progenitor cell	**Human**: Inconsistent across literature
CL:4030007	Fallopian tube multiciliated epithelial cell	**Mouse**: *Ccdc153*, *Dnah5*, *Foxj1*, *Tubb4b*
		**Human**: *CAPS*, *CATIP*, *DNAH5*, *FOXJ1*, *TUBB4B*
CL:4052019	Fallopian tube non-ciliated epithelial cell	**Mouse**: *Wnt7a*
CL:4030006	Fallopian tube secretory epithelial cell	**Mouse**: *Krt7*, *Ovgp1*, *Pax8*
		**Human**: *KRT7*, *MUC1*, *OVGP1*, *PAX8*
CL:4052022	Fallopian tube smooth muscle cell	**Mouse**: *Myh11*
		**Human**: *ACTA2*, *DES*, *MYH11*
CL:4033014	Peg cell	**Huma**n: *CD44*, *ITGA6*
Endothelial		
CL:0000115	Endothelial cell	**Human**: *CLDN5*
Immune		
CL:0000236	B cell	**Human**: *CD79A*, *MS4A1*
CL:0000624	CD4-positive, alpha-beta T cell	**Human**: *CD3E*, *CD4*, *IL7R*
CL:0000625	CD8-positive, alpha-beta T cell	**Human**: *CD3E*, *CD8A*, *GZMK*
CL:0000451	Dendritic cell	**Human**: *CD1C*, *CLEC10A*, *FCER1A*, *HLA-DRA*
CL:0000235	Macrophage	**Human**: *APOE*, *C1QA*, *C1QB*, *C1QC*, *CD163*, *FOLR2*, *TREM2*
CL:0000097	Mast cell	**Human**: *CPA3*, *KIT*, *MS4A2*, *TPSAB1*, *TPSB2*
CL:0000576	Monocyte	**Human**: *CD14*, *LYZ*, *S100A8*, *S100A9*, *FCN1*, *VCAN*
CL:0000623	Natural killer cell	**Human**: *GNLY*, *KLRC1*, *KLRD1*, *NKG7*, *PRF1*
CL:0000786	Plasma cell	**Human**: *IGHG1*, *JCHAIN*, *MZB1*, *SDC1*
Other stromal		
CL:0000057	Fibroblast	**Mouse**: *Col1a1*, *Dcn*, *Pdgfra*
		**Human**: *COL1A1*, *DCN*, *PDGFRA*

**Table 3.** Uterus cell types with Cell Ontology. Uterus cell types with Cell Ontology (CL) IDs and marker genes in mouse and human. Marker genes represent transcriptionally differentially expressed genes from the cited literature and should be interpreted as enriched rather than exclusively restricted to the indicated cell type. Expression of individual genes across multiple cell types is expected and does not preclude their utility as part of a marker gene panel.

CL ID	CL label (cell type)	Markers
Uterus-specific		
CL:4030040	Endometrial ciliated epithelial cell	**Human**: *CAPS*, *CCNO*, *CDC20B*, *EPCAM*, *FOXJ1*, *MUC12*, *PIFO*, *TPPP3*
CL:0002149	Epithelial cell of the uterus	**Human**: *EPCAM*, *MMP7*, *SOX9*
CL:0002656	Glandular endometrial unciliated epithelial cell	**Human**: *ABCG1*, *CBR3*, *CXCL8*, *DENND2C*, *DKK1*, *DPP4*, *EMID1*, *EPCAM*, *FOXA2*, *GPX3*, *HPRT1*, *IHH*, *KMO*, *LMO7*, *MMP26*, *MUC16*, *OPRK1*, *PAEP*, *PAX8*, *PHLDA1*, *PIMKLB*, *S100P*, *SCGB2A2*, *SLC7A11*, *SOX5*, *SOX9*, *SUFU*, *TSPAN8*
CL:4052050	Luminal epithelial cell of endometrium	**Human**: *CLDN22*, *EPCAM*, *LCN2*, *LEFTY1*, *LGR5*, *LPAR3*, *PTGS1*, *SAA1*, *SOX9*, *SULT1E1*, *WNT7A*
CL:0002255	Stromal cell of endometrium	**Human**: *ALDH1A*, *ATP5L*, *CD24*, *CD3E*, *NT5E*, *CDH5*, *CDKN1A*, *CRABP2*, *CTGF*, *DEPP1*, *FOXC1*, *FOXL2*, *FOXO1*, *GNLY*, *HAND2*, *HLA-A*, *HLA-C*, *HOPX*, *IGF1*, *IGFBP1*, *INHBA*, *INSR*, *MCAM*, *MMP10*, *MMP11*, *MYH11*, *OGN*, *PCOLCE*, *PDGFRB*, *PRKCDBP*, *RUNX3*, *STEAP4*, *SUSD2*, *THBD*, *TNFSF10*, *WNT4*
CL:4052028	Uterine natural killer cell	**Human**: *CCL5*, *CD69*, *HMGB2*, *NCAM1*
CL:0002601	Uterine smooth muscle cell	**Human**: *ACTA2*, *ACTG2*, *DES*, *LEFTY2*, *MYH11*
Endothelial		
CL:0002144	Capillary endothelial cell	**Human**: *ACTA2*, *CD44*, *COL1A1*, *COL1A2*, *CXCR4*, *EDNRB*, *ESM1*, *ESR1*, *KDR*, *MKI67*, *MYH11*, *PGR*, *PLVAP*, *STEAP4*, *ZEB2*
CL:1000413	Endothelial cell of artery	**Human**: *BMX*, *EFNB2*, *GJA4*, *GJA5*, *MECOM*, *SEMA3G*
CL:0002652	Endothelial cell of high endothelial venule	**Human**: *ICAM1*, *IL10*, *TIMP1*, *WNT5A*
CL:0002139	Endothelial cell of vascular tree	**Human**: *ACKR1*, *CD34*, *SEMA3G*
CL:1000414	Endothelial cell of venule	**Human**: *CCL15*, *EGR1*, *FOSB*, *ICAM1*, *KLF4*, *POSTN*, *SELE*, *SELP*, *TNFRSF1A*, *VCAM1*
CL:0002138	Lymphatic endothelial cell	**Human**: *CAVIN2*, *CD36*, *FXYD6*, *NRP2*, *PDPN*, *PROX1*, *TIE1*
Immune		
CL:0000236	B cell	**Human**: *CCL5*, *CD69*, *CXCR4*, *MS4A1*
CL:0000451	Dendritic cell	**Human**: *CD68*, *IL1B*
CL:0001065	Innate lymphoid cell	**Human**: *CCL5*, *CD69*, *CXCR4*, *IL7R*, *KIT*, *SPINK2*
CL:0000542	Lymphocyte	**Human**: *NCAM1*, *PTPRC*
CL:0000235	Macrophage	**Human**: *CD14*, *CD68*, *CSF1R*, *IL1B*, *SEPP1*
CL:0000097	Mast cell	**Human**: *CD69*, *CSF1*, *KIT*, *MS4A2*
CL:0000813	Memory T cell	**Human**: *CD69*, *MUC5B*, *RUNX3*, *SAA1*, *TFF3*
CL:0000084	T cell	**Human**: *CCL5*, *CD3G*, *CD69*, *CXCR4*, *HMGB2*, *IL7R*
Other stromal		
CL:0000057	Fibroblast	**Human**: *C7*, *CFH*, *CTSK*, *OGN*
CL:4033054	Perivascular cell	**Human**: *ACTA2*, *FHL5*, *GUCY1A2*, *MYH11*, *NTRK2*, *RGS5*, *STEAP4*

Throughout this review, the term “marker gene” refers to genes identified as differentially expressed in a given cell population relative to others within a transcriptomic dataset and should not be conflated with protein markers used in antibody-based detection methods such as immunohistochemistry. Marker genes are combinatorial by nature: No single gene is expected to uniquely identify a cell type in isolation, and the gene sets provided in Tables 1 to 3 should be applied as combinatorial enriched signatures and validated spatially or orthogonally where possible, as demonstrated by Morris *et al.* (*16*) using RNA in situ hybridization.

While the FRT is an incredibly dynamic and responsive system, the scope of this proposal is limited to the baseline state of the female reproductive adult (cycling, nonpregnant, ovaries, FTs, and uterus). To ensure that this resource is broadly relevant to all female reproductive biologists, not limited to the species they work on, this paper reviews cell types identified across the two most commonly studied mammals: mouse and human. Recent comparative single-cell and spatial studies have identified both pronounced species-specific differences (*17*) and conserved transcriptional features within the ovary (*18*), with similar observations reported in other female reproductive studies (*19*, *20*). Hence, it is important to account for interspecies variation when referring to the cell types and markers mentioned in this review. In the ovary specifically, some markers are shared between human and mouse, whereas others are not, and even shared markers may differ in their specificity or relative utility for defining a given cell identity. Accordingly, annotation and label transfer across datasets and species are best supported by broader transcriptional signatures or multigene modules rather than by single marker genes alone, particularly in single-cell datasets where transcript nondetection may reflect technical dropout or differences in sequencing depth rather than true biological absence. Such strategies should improve interpretability and facilitate future cross-species studies, which will be essential for defining both conserved and species-specific transcriptional programs across the FRT.

The rapid growth of high-throughput single-cell and spatial technologies makes it essential for the field to align on a resting-state nomenclature as a baseline for future work. Establishing this reference point will enable robust cross-study integration and facilitate the discovery of new biological insights into health, aging, and disease of the FRT. As additional datasets incorporating spatial, temporal, and functional context become available, definitions of existing cell types will continue to be refined, and new cell types or states may emerge. Extending reproductive cell taxonomy and nomenclature to additional reproductive stages, including prenatal development, puberty, pregnancy, perimenopause, menopause, and diseased states, will require future community efforts beyond the scope of this paper. Similarly, comprehensive annotation of other components of the FRT, such as the cervix, vagina, and external genitalia, will depend on continued data generation and coordinated ontology updates. Where possible, proposed cell type boundaries are anchored to functional distinctions such as steroidogenic capacity, spatial position within the follicle, or irreversible changes in cell state, rather than transcriptional clustering alone, ensuring that the ontology reflects biological reality rather than technical artifact. The cell type markers (Tables 1 to 3 and table S1) and ontology terms proposed here (Fig. 2) reflect the best available evidence at the time of writing and are intended to evolve and be updated as new data emerge in the field, consistent with how the Cell Ontology is maintained by the Open Biological and Biomedical Ontology Foundry.

**Fig. 2.**
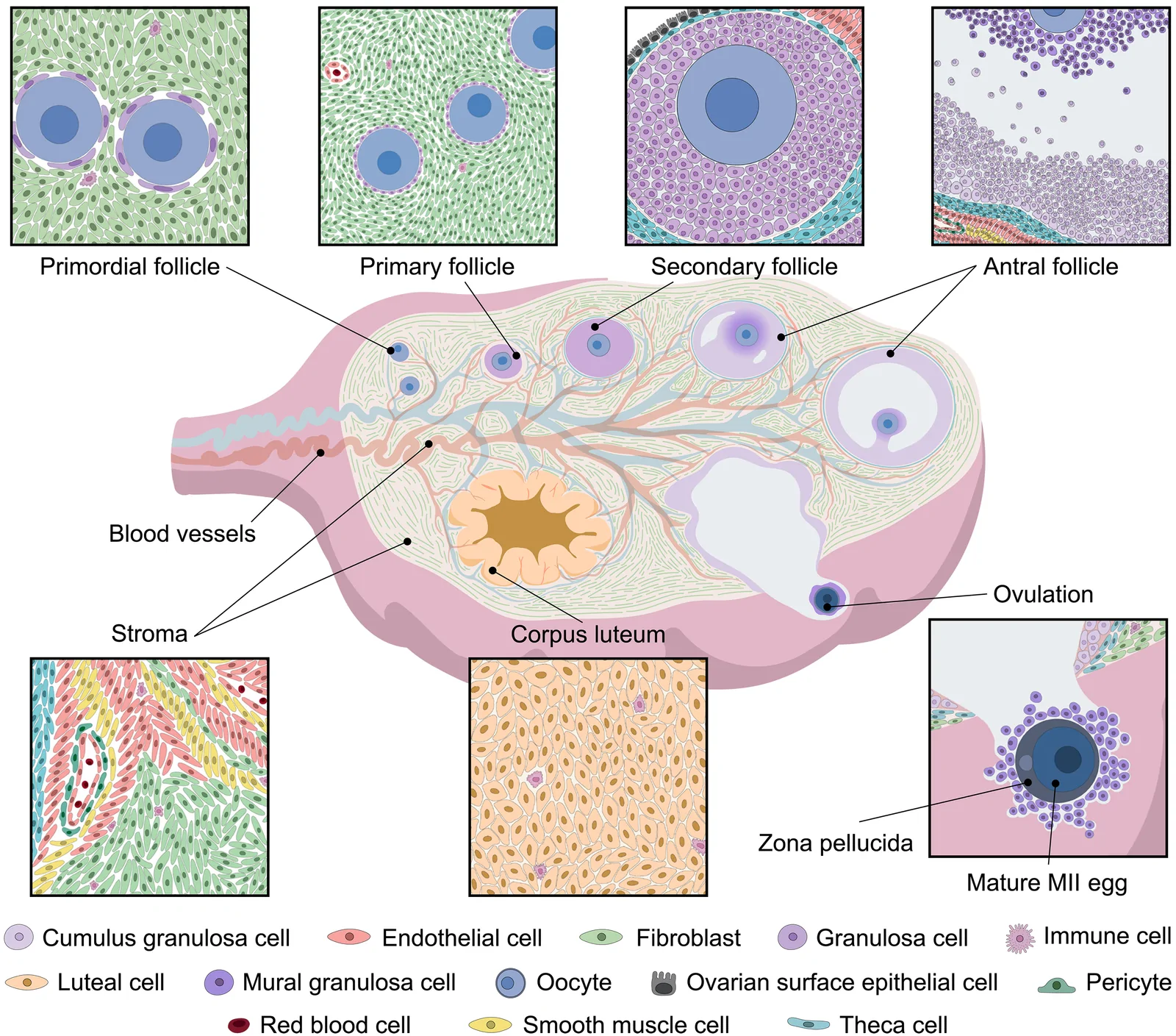
Illustration of folliculogenesis. Overview of folliculogenesis in the ovary and the cell types present. Figure created by L. S. E. Kim using Adobe Illustrator (Adobe Inc., San Jose, California).

Last, a balance must be maintained between detailed classification of tissue-specific subpopulations and higher-level terminology that supports integration across organ systems. While identifying specific cell subtypes is important for understanding tissue-specific biology, standardized overarching terminology will facilitate comparisons across atlases. This approach will allow comprehensive cross-tissue comparisons and a unified perspective on human health and disease. Future data generation should bridge the gap between detailed tissue-specific insights and high-level annotations, ensuring that the evolving taxonomy is both precise and broadly applicable.

## CONSENSUS PROCESS FOR ONTOLOGY DEVELOPMENT AND IMPLEMENTATION

The cell type terms proposed in this manuscript were developed through a structured, in-person consensus meeting spanning four full days, attended by coauthors representing 19 principal investigator groups with active research programs in single-cell and spatial transcriptomics of the FRT. Before the meeting, attendees contributed either published dataset repository identifiers or referenced unpublished datasets from their own laboratories, forming the empirical basis for discussion.

During the meeting, attendees were divided into organ-specific working groups covering the ovary, FT, and uterus. Each group followed a three-stage process: first, curation of marker genes from contributed published and unpublished datasets with associated references; second, discussion and agreement on the biological criteria defining each cell type within that organ and the rationale for proposed parent-child relationships; and third, presentation of the proposed hierarchy to the full group for collective review and final agreement before implementation.

Within each organ group, attendees were further subdivided into cell type–specific subgroups to curate marker gene sets and draft the proposed ontology hierarchy. Each proposed hierarchy was then presented to the full assembly, where attendees discussed whether the classification accurately reflected the underlying biology and how it should be reconciled with existing, often incorrect, Cell Ontology terms. Where disagreements arose, both positions were presented and discussed until a resolution was reached that addressed all concerns. Decisions were guided primarily by ease of use for researchers entering the field, biological accuracy, and consistency across the three organs, rather than by individual preference or historical convention within any single research group.

An illustrative example of this process was the classification of ovarian stromal cells, where attendees acknowledged that the heterogeneity of this compartment remains incompletely understood, for instance, whether mechanically activated fibroblasts represent a distinct cell state or type remains unresolved. In contrast, attendees working on the uterus noted that “stromal cell” has a well-established conventional meaning in endometrial biology, referring to the predominant mesenchymal population that undergoes cyclical remodeling. These cross-organ considerations were discussed collectively, with the final classification representing the consensus visible below.

Following agreement, formal ontology updates including parent-child relationship mappings, correction of preexisting misclassifications, and cross-ontology relationship axioms linking new terms to Uberon anatomical structures were implemented directly into the Cell Ontology repository and CELLxGENE by coauthors with ontology development expertise (A.P.-B., C.E., and B.A.), ensuring immediate computational accessibility. All proposed terms have been assigned stable CL identifiers and are publicly queryable via www.ebi.ac.uk/ols4/ontologies/cl. Full formal logical definitions; OWL axioms; equivalence axioms with necessary and sufficient conditions; relationship mappings to external ontologies including Uberon, GO, PATO, and Protein Ontology; disjointness declarations between sibling terms; synonym provenance; and GitHub issue tracking for all new and modified terms are provided in Supplementary Text 1. Governance for future updates follows the standard Cell Ontology repository process, where proposed changes are submitted as GitHub issues, reviewed by ontology curators, and accepted on the basis of community consensus and biological evidence.

## OVARY

The ovaries (Fig. 2 and figs. S2 and S3) are the gonads that typically develop in XX mammalian individuals, serving both reproductive and endocrine functions. The ovary contains a unique functional unit called the follicle, which consists of a germ cell (oocyte) surrounded by granulosa cells and, later in development, theca cells. Primordial follicles are continuously activated to initiate growth throughout postnatal life; however, it is only at puberty that the hypothalamic-pituitary-gonadal axis signaling enables follicles to progress beyond the gonadotropin-independent stages and mature into antral follicles through the process of folliculogenesis. During this process, the oocyte develops from an incompetent oocyte paused in prophase of meiosis I to a competent egg in meiosis II at ovulation. Following ovulation, the theca and granulosa cells left behind luteinize to form the corpus luteum, producing progesterone that signals to the uterine lining to prepare for and support a pregnancy. If there is no implantation, the corpus luteum regresses.

Beyond the follicles, the ovary is embedded within a complex stromal microenvironment that supports follicle development, vascular remodeling, hormonal signaling, and tissue homeostasis. This ovarian stroma comprises multiple interacting cell types, including fibroblasts, endothelial cells, smooth muscle cells, pericytes, immune cells, peripheral nerves, and associated glial cells. However, the definition of ovarian stroma has not been standardized, and many datasets annotate these diverse populations under a single broad “stroma” label. Here, we propose that, where possible, large stromal clusters should be resolved into specific constituent cell types, including ovarian epithelial cells, endothelial cells, smooth muscle cells, pericytes, immune cells, fibroblasts, and neural and glial populations, while retaining theca progenitor cells and steroidogenic stromal cells as ovary-specific stromal lineages. These stromal populations are discussed in more detail in the subsequent section on stromal cell types. The updated cell type hierarchy for the ovary is summarized in Fig. 3, and marker genes enriched for each cell type are listed in Table 1.

**Fig. 3.**
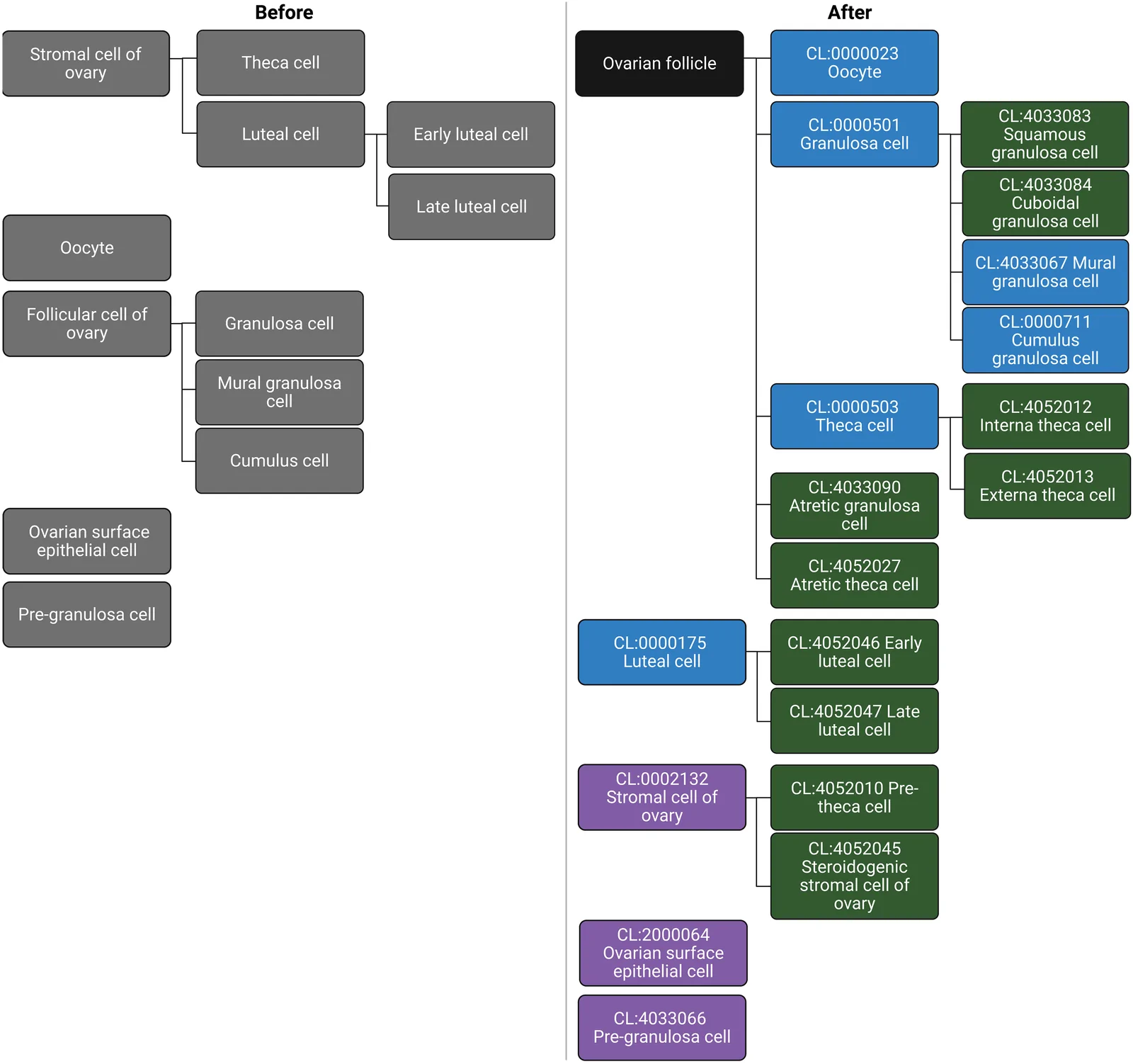
Updates to ovarian Cell Ontology. Updates to the cell type hierarchies of the ovary from before (left) to after (right) in the Cell Ontology following the jamboree. Changes to ovarian cell classifications include newly added terms (green), revised or reclassified terms (blue), and preexisting terms (purple). In the ovarian diagram, the term “ovarian follicle” (black) is included to represent its anatomical context, linking relevant cell types to the Uberon ontology. Created in BioRender. Ong, H.T. (2026), https://biorender.com/l7ba6or.

### Oocytes

During folliculogenesis, oocytes activate from dormancy, begin transcribing and storing mRNAs in preparation for fertilization, and undergo tremendous volumetric expansion, growing from ∼20 μm to up to 120 μm (in humans) (*21*, *22*). Each of these steps occurs within or throughout several stages of folliculogenesis. Although follicle growth relies on close coordination between oocytes and granulosa cells, these two cell types are biologically distinct and should be studied as separate entities. To elucidate the relationship between these cell types, the Cell Ontology has separate entries for “oocyte” (http://purl.obolibrary.org/obo/CL_0000023), “granulosa cell” (http://purl.obolibrary.org/obo/CL_0000501), and “theca cell” (http://purl.obolibrary.org/obo/CL_0000503), each of which is “part of” the Uberon term “ovarian follicle” (http://purl.obolibrary.org/obo/UBERON_0001305) (Fig. 2). Recent advances in single-cell RNA sequencing (scRNA-seq) technologies and refined mechanical isolation techniques have enabled transcriptomic profiling of oocytes at discrete stages of folliculogenesis (*23*, *24*). However, later-stage oocytes often exceed the size limitations of common single-cell sequencing workflows and must be selectively filtered from single-cell suspensions, enriched, and sequenced separately (*25*). Still, genes with enriched expression in oocytes have been reported, including *ZP2*, *DDX4*, *SYCP3*, *SOX30*, *ZAR1*, *DAZL*, *YBX2*, and *LHX8* (*24*). However, these oocyte-specific genes correspond mainly to oocytes at later stages of follicle development, and it remains possible that as oocytes progress through folliculogenesis, their gene profiles shift. For example, the first milestone in oocyte development is the transition from a quiescent state within primordial follicles to an activated state within primary follicles. Between these two stages lies the “transitioning” or “transitional” primordial follicle state, which is most clearly defined by changes in the surrounding granulosa cells (discussed below). Given the subtlety in this transition, ongoing studies are investigating whether oocytes at primordial, transitioning, and primary stages exhibit distinct transcriptional profiles that reflect the shift from dormancy to activation in mouse (*26*) and human oocytes (*27*). Similarly, as oocytes progress in development from primary to pre-ovulatory stages, changes in mRNA transcription may distinguish them from one another. Notably, a recent study concluded that classifying follicles as dormant or active based solely on morphology is insufficient and recommends integrating morphology with transcriptomic data for accurate staging (*28*). To improve clarity and consistency in future studies, we propose adopting a standardized nomenclature that specifies the follicle stage of origin when describing oocytes (e.g., “primary follicle oocyte”) (*29*). Although there is not yet enough evidence to propose follicle stage–specific oocyte markers, results from future studies will elucidate how the oocyte transcriptome shifts across folliculogenesis and whether oocyte gene expression alone is sufficient to inform follicle stage annotation without relying on histology context. As it stands currently, oocyte-specific marker genes identified are curated in Table 1, with specific references for each marker gene listed in table S1.

### Granulosa cells

Granulosa cells function as the primary support cells for developing oocytes. They provide metabolic support to the oocyte through the transport of amino acids, pyruvate, and other metabolites via specialized cellular projections known as transzonal projections (TZPs), which extend from granulosa cells across the zona pellucida to contact the oocyte (*30*). Notably, TZPs are largely absent in primordial follicles and form during the transition to the primary follicle stage, coinciding with granulosa cell cuboidalization and increased oocyte-somatic cell communication.

In primordial follicles, which make up the ovarian reserve, a single layer of granulosa cells surrounds the oocyte. These cells exhibit a squamous morphology and express *FOXL2* (*31*). Squamous granulosa cells are also referred to as flattened or pre-granulosa cells. The term “pre-granulosa cell” (http://purl.obolibrary.org/obo/CL_4033066) is additionally used to describe precursor cells that give rise to squamous granulosa cells during primordial follicle formation in the fetal ovary (*32*). Future direct comparisons of these populations may clarify whether they are transcriptionally distinct beyond their temporal separation. Follicles transitioning between the primordial and primary stages contain a mixture of squamous and cuboidal granulosa cells. By the primary follicle stage, granulosa cells uniformly adopt a cuboidal morphology, from which point onward they remain cuboidal throughout folliculogenesis. Cuboidal granulosa cells, also referred to as columnar granulosa cells, proliferate to form multiple layers in secondary follicles and establish extensive TZP-mediated contacts with the growing oocyte. Although follicle growth up to the secondary stage is largely gonadotropin-independent, expression of gonadotropin receptors such as FSHR (follicle-stimulating hormone receptor) has been detected in subsets of early preantral follicles (*33*), suggesting earlier hormonal responsiveness than traditionally appreciated.

Upon formation of the antrum, follicle development becomes fully gonadotropin-dependent, and granulosa cells segregate into two functionally and spatially distinct subtypes: mural granulosa cells and cumulus granulosa cells. Mural granulosa cells line the follicle wall adjacent to the basal lamina and vasculature, express high levels of aromatase (CYP19A1), and are responsive to luteinizing hormone (LH). These cells convert androstenedione produced by theca cells into estradiol, which blocks LH secretion from the pituitary gland. Once estradiol levels increase to reach a certain threshold, the LH surge is initiated, which induces ovulation. In contrast, cumulus granulosa cells, named for their cloud-like (“cumulus”) appearance, form a compact structure immediately surrounding the oocyte, maintain dense TZP-mediated contacts, and play a central role in oocyte metabolic support, meiotic competence, and ovulation-associated expansion. The cumulus cell layer directly surrounding the oocyte is called the corona radiata. Oocyte-secreted factors promote the differentiation of the granulosa cells nearest to them into cumulus cells. The LH surge induces a signaling cascade through which expansion of the cumulus-oocyte complex (COC) is initiated. Oocyte-secreted factors, as well as proteins translated in granulosa cells, contribute to the expansion process, which occurs primarily through the production of a hyaluronan-rich matrix, among other extracellular matrix (ECM) components. Key COC expansion genes expressed by cumulus cells include *Has2*, *Sult1e1*, *Lox*, *Emb*, *Btc*, *Tnfaip6*, and *Cox2* in mice and *TNFSF11*, *PAPPA*, *LHCGR*, *INHBB*, *PTGS1*, *PTX3*, *BMPR2*, *SEMA3A*, *CD200*, *CD24*, and more in humans (*34*, *35*). COC expansion is critical for ovulation and fertilization and is thought to promote expulsion of the COC from the follicle during follicle rupture.

Previously, CL lacked the granularity to map the distinct morphological and functional shifts across folliculogenesis, grouping diverse cells simply as “granulosa cells.” To resolve this and more accurately reflect these developmental changes, the Cell Ontology has been updated such that “granulosa cell” is now a parent term for two newly added terms [“cuboidal granulosa cell” (http://purl.obolibrary.org/obo/CL_4033084) and “squamous granulosa cell” (http://purl.obolibrary.org/obo/CL_4033083)], alongside reclassified terms [“mural granulosa cell” (http://purl.obolibrary.org/obo/CL_4033067) and “cumulus granulosa cell” (http://purl.obolibrary.org/obo/CL_0000711)] (Fig. 3). However, the number of reliable markers for preantral follicles is limited, making cell annotation of granulosa cells challenging. Because of the low abundance of squamous granulosa cells, they are not reliably detected in single-cell datasets. Cumulus granulosa cells of antral follicles before cumulus expansion and the cuboidal granulosa cells of preantral follicles are challenging to separate, as they have a large functional overlap in supporting oocyte growth through physical connections. However, the advent of spatial technology has helped facilitate granulosa cell subclustering by adding a morphological component to help identify granulosa cells from specific follicle stages in addition to known markers. A proof of principle is provided by Lan *et al.* (*8*), where spatially resolved granulosa cell populations in mouse ovarian tissue are distinguishable by proximity to the oocyte and differential expression of *Kctd14*, *Ccnd2*, and *Serpine2*, consistent with the preantral and antral classifications proposed here. Spatial information will also promote the identification of new granulosa cell subtype markers, and current marker genes are listed in Table 1.

### Theca cells

Along with granulosa cells, theca cells are the hormone-producing cells of the follicle. In the traditional two-cell-two-gonadotropin theory, theca cells express CYP17A1, which allows them to convert cholesterol into androstenedione, and then, granulosa cells expressing CYP19A1 (“aromatase”) take up androstenedione and convert it into estradiol. Given that theca cells do not express CYP19A1 and granulosa cells do not express CYP17A1, steroid hormone production in the follicle is a coordinated effort between these two cell types. Separated from the oocyte and granulosa cells by the basement membrane, theca cells can be identified morphologically starting at the secondary (preantral) follicle stage. Beginning at the antral stage, theca cells differentiate into the theca interna and the theca externa (*36*). At ovulation, theca cells become luteinized, as discussed in the following luteal cell section. The terms “theca interna” and “theca externa” have been used to refer to specific theca cells found in those layers. The theca interna layer becomes highly vascularized and innervated, while the theca externa layer is enriched with smooth muscle cells and nerves. Historically, the Cell Ontology relied on a single, overarching “theca cell” term, which lacked the necessary granularity to distinguish these functionally and transcriptomically divergent layers. To align the ontology with these distinct biological roles and enable accurate single-cell annotation, we restructured the hierarchy by establishing the preexisting “theca cell” term as a parent class and introducing two newly defined child classes: “interna theca cell” (http://purl.obolibrary.org/obo/CL_4052012) and “externa theca cell” (http://purl.obolibrary.org/obo/CL_4052013) (Fig. 3).

The primary role of theca interna cells is the production of hormones. Transcriptionally, these cells are characterized by genes related to steroidogenesis and steroid metabolism. Genes that demarcate theca externa cells include ECM components, as shown in table S2. Recent insights from single-cell datasets have suggested that there may be additional theca subtypes based on expression of marker genes and proximity to the granulosa cells and basement membrane. The use of “structural theca” and “androgenic theca” have been proposed as alternatives to the theca externa and theca interna, respectively, as well as the emergence of a possible new subtype, “peri-follicular theca” (*37*). The current list of marker genes annotating the theca cell populations is listed in Table 1, and references are listed in table S1. Future integration with spatial transcriptomics will aid in further distinctions between theca subtypes.

### Luteal cells

Following the rupture of the antral follicle, the corpus luteum forms as a transient endocrine structure that produces progesterone to support early pregnancy. However, in the absence of fertilization, progesterone production declines and the corpus luteum undergoes regression, accompanied by immune cell recruitment. The timing and life span of this structure vary between species; for example, in mice, regressing corpora lutea can persist across multiple cycles (*38*). Although early and late luteal phases are well recognized physiologically, the exact point at which an active corpus luteum transitions to a regressing one remains difficult to define transcriptomically (*8*). For this reason, the Cell Ontology includes both “early luteal cell” (http://purl.obolibrary.org/obo/CL_4052046) and “late luteal cell” (http://purl.obolibrary.org/obo/CL_4052047) under the parent term “luteal cell” (http://purl.obolibrary.org/obo/CL_0000175), allowing future datasets to refine distinctions among these post-ovulatory states (Fig. 3).

Within mouse single-cell data, two broad functional populations of luteal cells are consistently detected. Early or active luteal cells, representing the progesterone-producing phase, express receptors required for luteotropic signaling such as the LH receptor (*Lhcgr*) and prolactin receptor (*Prlr*) (*39*, *40*). In contrast, late or regressing luteal cells down-regulate steroidogenic pathways and express prostaglandin F receptor (*Ptgfr*), which promotes corpus luteum regression (*41*, *42*). These populations also segregate spatially, with newly formed corpora lutea enriched for early/active luteal cells, whereas older corpora lutea contain regressing populations (*16*).

The corpus luteum originates from theca and granulosa cells remaining after follicle rupture (*43*). Consequently, the descriptors “luteal,” “lutein,” and “luteinizing” are used to denote cells that have undergone luteinization and are transcriptomically distinct from mural theca and granulosa cells (*44*). Historically, luteal cells were divided into large (granulosa-derived) and small (theca-derived) populations on the basis of classical work in primates and ruminants (*42*, *45*). However, modern transcriptomic datasets have not been able to reproduce this lineage-based division, and thus, the origin of luteal cells cannot be inferred from gene expression alone (*8*, *46*).

Recent single-cell and spatial transcriptome studies further highlighted transcriptional heterogeneity within luteal cells. Lan *et al.* (*8*) identified two luteal subclusters enriched for *Parm1* and *Akr1c18*, while Zhao *et al.* (*46*) described related populations marked by *Bhmt* or *Onecut2*. Morris *et al.* (*16*) similarly reported one luteal cluster associated with ongoing steroidogenic activity, enriched for proliferative markers such as *Top2a*, and a second cluster showing features of regression, including expression of *Cdkn1a*, *Sdc4*, *Cldnd1*, and *Btg1*. Although naming conventions differs across studies, these findings collectively highlight the presence of multiple luteal subpopulations distinguished by transcriptional signatures rather than clearly defined lineages.

In human and nonhuman primate datasets, luteal cells are detected at low abundance, hindering the ability to identify luteal subpopulations. This challenge is particularly relevant in mono-ovulatory species, where only a single corpus luteum forms each cycle and donor menstrual phase or contraceptive use is often unknown. Experimental design must therefore be considered carefully when attempting to capture and characterize luteal populations in human tissues.

### Stromal cell of the ovary: Pre-theca, steroidogenic, and unresolved populations

The distinction between pre-theca cells, theca cells, and steroidogenic stromal cells is difficult to discern, often leading to inconsistent annotations and a lack of resolution. Steroidogenic stromal cells are characterized by their expression of steroidogenic markers, *STAR*, *CYP11A1*, and *CYP17A1*. These cells share common characteristics with fibroblast-like stromal cells and steroid-producing theca cells. Cells in the ovarian stroma that later become theca cells have been described using various terms, including theca progenitor, theca precursor, pre-theca cell, early theca cell, and ovarian interstitial cells/glands (*36*, *47*), leading to fragmentation in nomenclature across the field. Instead, we propose to use the term “pre-theca” to describe this cell population. Previously, these ovary-specific cell types were absent from the Cell Ontology, which now includes new terms for “pre-theca cell” (http://purl.obolibrary.org/obo/CL_4052010) and “steroidogenic stromal cell of the ovary” (http://purl.obolibrary.org/obo/CL_4052045) as child terms under the preexisting parent term “stromal cell of the ovary” (http://purl.obolibrary.org/obo/CL_0002132) (Fig. 3). Future spatial and multiomics integration will further improve distinctions between these cell types.

Within this hierarchy, the broader stromal cell of ovary category represents a remainder population, cells residing within the ovarian stroma that cannot currently be resolved into more specific constituent subclasses including fibroblasts, endothelial cells, pericytes, smooth muscle cells, immune cells, or neural and glial populations, based on available transcriptomic data. This population is therefore defined in practice by the absence of markers that would assign it to one of these better-characterized subclasses, rather than by unique positive markers of their own. This reflects a genuine gap in current understanding rather than an oversight, as the transcriptional heterogeneity of the ovarian stroma remains incompletely resolved. Users are encouraged to first annotate the well-characterized stromal subpopulations using the marker genes provided, treating any remaining unassigned stromal clusters as candidates for this broad category. Iterative subclustering and spatial validation are recommended to progressively resolve their identity as new data become available.

### Ovarian surface epithelium

The ovarian surface epithelium is a monolayer of cuboidal mesothelial cells that encase the ovary and respond to cyclical ovarian hormones, including estrogen, progesterone, and gonadotropin-driven ovulatory cues (*48*, *49*). As the outermost layer of the ovary, it is situated above a basement membrane that attaches it to the tunica albuginea (*50*, *51*). Before ovulation, hormone fluctuations prompt the ovarian surface epithelium to produce proteolytic enzymes that break down surrounding tissue and activate TNF-α (tumor necrosis factor–α) signaling, leading to cell death above the antral follicle (*52*). Together, this enzymatic activity and TNF-α signaling facilitate the thinning of the ovarian wall at the future site of ovulation. However, the functional relevance of these activities has remained a point of contention in the field since the 1970s (*53*, *54*). The cyclic disruption of the ovarian surface epithelium during ovulation necessitates near-constant activation of wound healing and regeneration pathways. Now, few markers show enriched expression in the “ovarian surface epithelial cell” (http://purl.obolibrary.org/obo/CL_2000064), including *UPK3B* and *LRRN4* (*55*, *56*).

## FALLOPIAN TUBE

The FTs or oviducts in mice (Fig. 4 and figs. S4 and S5) are conduits between the ovary and the uterus that facilitate the transport and fertilization of the egg, as well as sperm capacitation. Structurally, the FTs can be divided into five main parts: fimbriae, infundibulum, ampulla, isthmus, and uterotubal junction (or “intramural region” in humans). The FT epithelium (FTE) is also thought to harbor the cell of origin for tubo-ovarian high-grade serous carcinoma (HGSC), which through a known series of cell state transitions results in oncogenic transformation (*57*–*59*). Given the importance of this tissue in HGSC, recent work has examined the impact of a range of clinical and patient characteristics, such as *BRCA1/2* mutations (*60*), menstrual cycle phase (*61*), and the contribution of transposable element expression to oncogenic transformation within the FTs (*62*). Together, characterization of cell type and cell state heterogeneity is critical to our understanding of development and disease within the FTs. The updated cell type hierarchy for the FT is summarized in Fig. 5, and marker genes for each cell type are listed in Table 2. Historically, three major cell types have been described in the primary human FTE: (i) nonciliated secretory epithelial (NCSE) cells, (ii) ciliated epithelial (CE) cells, and (iii) progenitor-like cell populations (*63*, *64*).

**Fig. 4.**
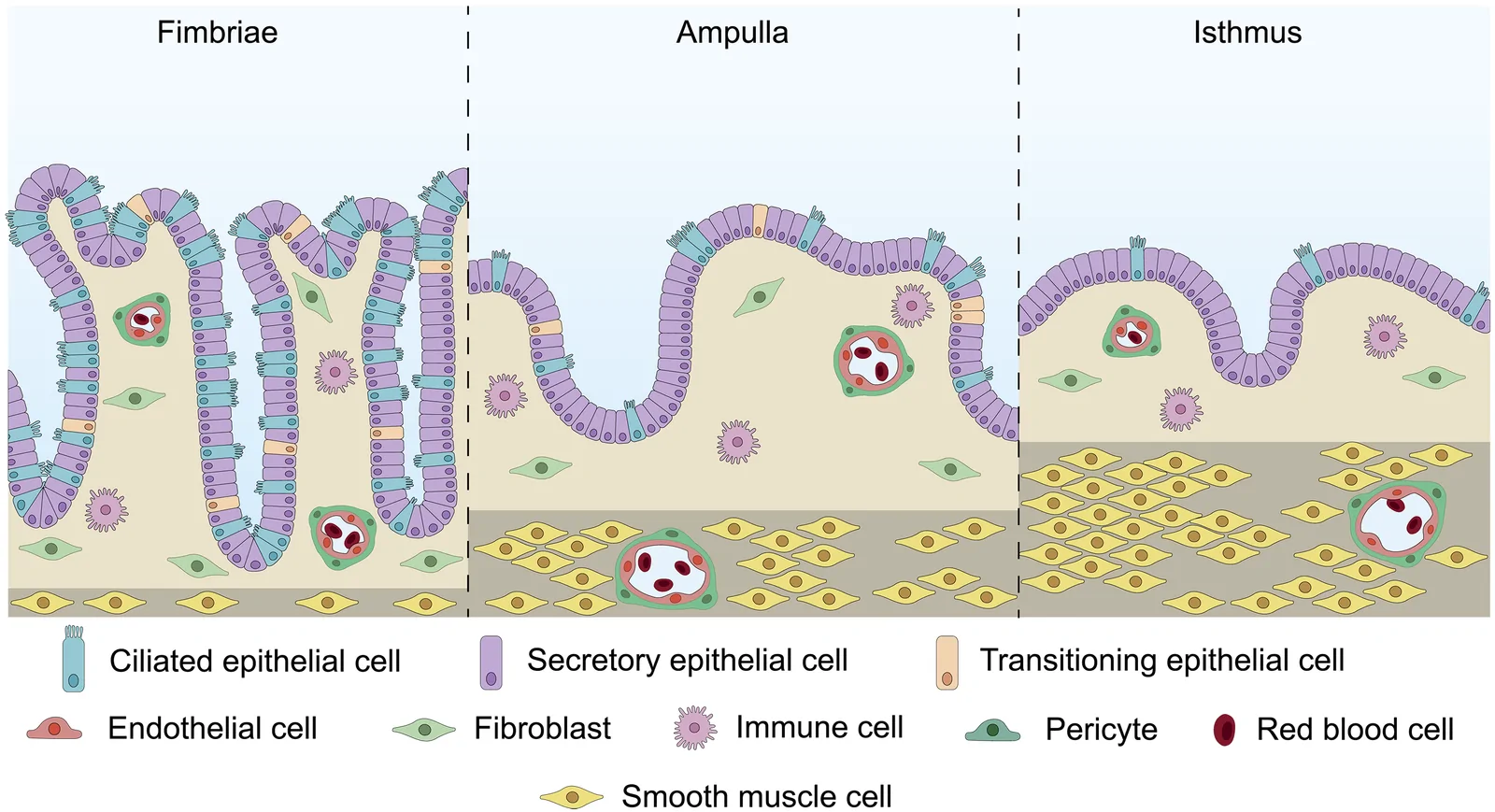
Illustration of the FT. Overview of the FT fimbriae, ampulla and isthmus segments, and the cell types that are present. Figure created by L. S. E. Kim using Adobe Illustrator (Adobe Inc., San Jose, California).

**Fig. 5.**
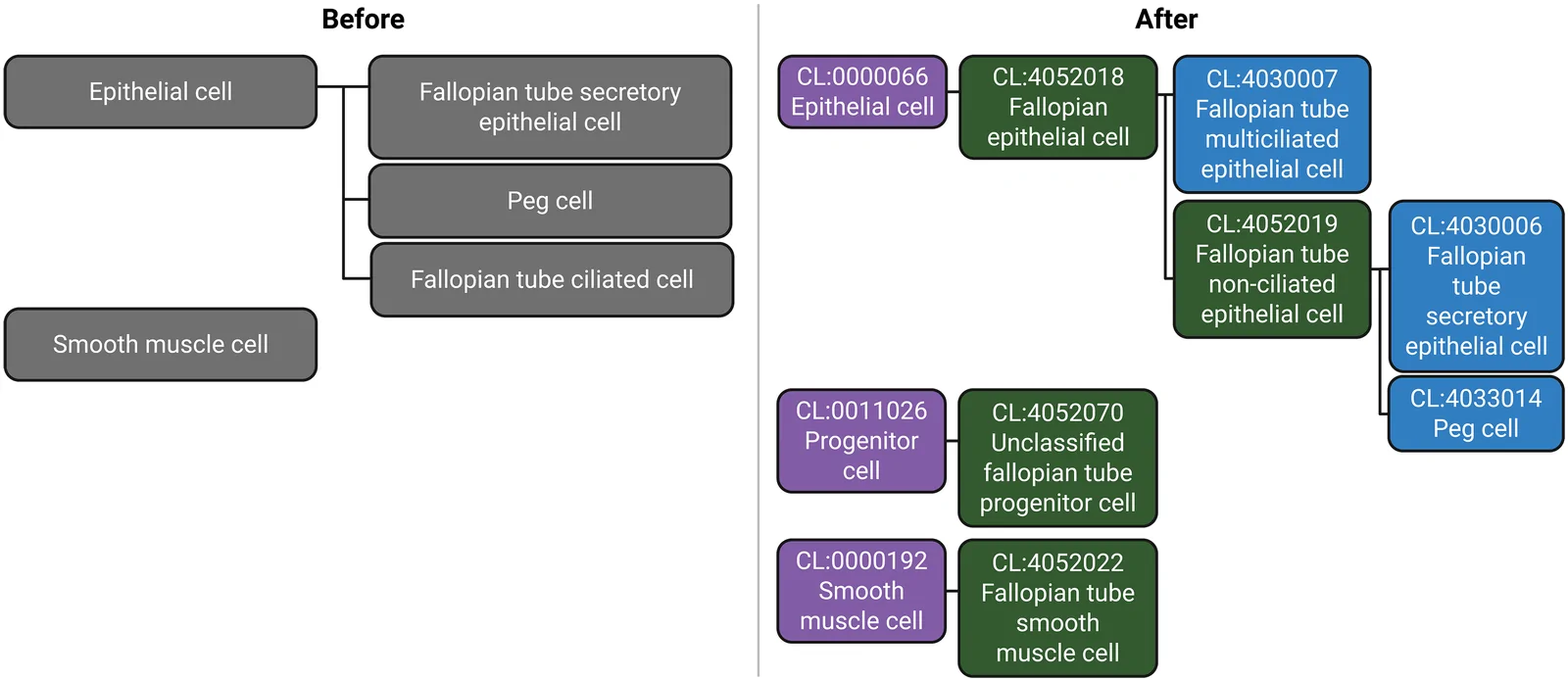
Updates to FT Cell Ontology. Updates to the cell type hierarchies of the FT from before (left) to after (right) in the Cell Ontology following the jamboree. Changes to FT cell classifications include newly added terms (green), revised or reclassified terms (blue), and preexisting terms (purple). Created in BioRender. Ong, H.T. (2026), https://biorender.com/wfuo5jy.

### Ciliated epithelial (CE) cells

The FTE is mainly composed of ciliated and secretory cells. CE cells are specialized cells characterized by the presence of hair-like structures called cilia, which play a key role as sperm reservoirs (*65*, *66*) and in the movement of the COC before fertilization from the ovary toward the uterus (*63*, *67*). These specialized cells also assist in the transport and clearance of fluid toward the uterus, which are essential for fertilization and implantation. Ciliated cells are more abundant in the fimbriated end of the FT, and their abundance decreases toward the isthmus, the closest section to the uterus (*68*, *69*). In addition, the number of ciliated cells in the FTE fluctuates during the ovulatory cycle and menopause as a result of hormonal changes, particularly in estrogen and progesterone. Estrogen stimulates cilium development and activity, whereas progesterone reduces ciliation (*70*, *71*). Recent scRNA-seq studies have provided new insights into the proportions and abundance of ciliated cells at pre- and postmenopause (*61*, *72*) and development stages (*73*). Complementing these findings, a large bulk transcriptomic study incorporating a greater number of individuals demonstrated depletion of ciliated cells in both postpartum and postmenopausal FTs (*60*). To accurately reflect the underscore dynamic regulation of ciliated cell proportions across physiological states, the Cell Ontology has been updated such that “fallopian tube epithelial cell” (http://purl.obolibrary.org/obo/CL_4052018) is now a parent term for the revised term “fallopian tube multiciliated epithelial cell” (http://purl.obolibrary.org/obo/CL_4030007) and the newly added term “fallopian tube non-ciliated epithelial cell” (http://purl.obolibrary.org/obo/CL_4052019) (Fig. 2).

These scRNA-seq studies have also identified different cell subtypes and states within ciliated cells. *FOXJ1*, a transcription factor critical for ciliogenesis, is a common marker used for annotation of CE cells (*74*). In addition, *CAPS* has been reported as a marker for ciliated cells in normal FTE and certain epithelial ovarian cancers, often coexpressed with *FOXJ1*, which reinforces its role in ciliogenesis and the functionality of the cilia (*75*, *76*). The cytoskeletal marker associated with microtubules, *TUBB4*, is often used to identify ciliated cells in single-cell studies and immunohistochemistry. Other markers showing variability across the literature include *DNAH5*, *RFX3*, *CCDC17*, and *CCDC78* (*77*). In mice, a recent scRNA-seq study showed that preciliated epithelial cells, marked by *Krt6*+, *Prom1*+, and *Trp73*+, are susceptible to HGSC (*59*).

### Nonciliated secretory epithelial (NCSE) cells

The NCSE cells are characterized by secretion of factors essential for oocyte health, fertilization, and early embryo development. While NCSE cells are distributed throughout the FTs, they are most abundant in the ampulla, comprising about 50% of the epithelia (*63*). With advancing age, the proportion of NCSE cells decreases, likely reflecting reduced reproductive activity. The proportions of NCSE cells are also influenced by hormonal fluctuations associated with different phases of the menstrual cycle, such as the proliferative and secretory phases, as well as by menopausal status (*73*). NCSE cells have been proposed as the cell of origin of HGSC, an aggressive histotype of ovarian cancer (*78*, *79*). PAX8, a transcription factor predominantly expressed in NCSE cells of the FTs, is often highly expressed in HGSC and, combined with the lack of observable ciliated cells by microscopy, supports the hypothesis that HGSC may originate from the FTs with NCSE cell characteristics (*57*, *80*, *81*). However, recent work has shown that some HGSC tumors retain gene expression programs found in CE cells (*60*). scRNA-seq studies have identified subpopulations of NCSE cells across different phases of the menstrual cycle, reproductive status, and development phases, supporting the dynamic changes occurring in these cells and the broader tissue in response to cyclic hormonal fluctuation within the FTs (*60*, *61*, *73*). Different gene expressions and cell surface markers have been used to identify NCSE cells, such as the general epithelial marker *EPCAM* and other well-established NCSE cell–specific markers including *PAX8* and *OVGP1*. A bulk study with over a hundred normal human FT samples (*60*) showed that these canonical NCSE cell markers fluctuate across menstrual cycle phases, with *OVGP1* expressed higher in the follicular phase and *PAX8*/*MUC1*/*KRT7* in the luteal phase. Multiple NCSE cell states have also been identified using single-cell studies on the basis of expression of markers such as *OVGP1* and *CXCL2* and the function associated with that cell state (*61*). Historically, the CL lacked a cohesive hierarchy to capture the diversity within the nonciliated epithelial compartment, often conflating actively secreting cells with functionally distinct ones, such as peg cells. To resolve this ambiguity and improve cross-study integration, the Cell Ontology (Fig. 5) has been updated to include “fallopian tube non-ciliated cell” (http://purl.obolibrary.org/obo/CL_4052019) as a parent term for the “fallopian tube secretory epithelial cell” (http://purl.obolibrary.org/obo/CL_4030006) and “peg cell” (http://purl.obolibrary.org/obo/CL_4033014), which are further described below.

### Unclassified FT progenitor cells

Stem and progenitor populations have long been sought after within the human FT and mouse oviduct alike. Multiple candidate populations and marker sets have been proposed, yet with limited overlap across studies (*61*, *63*, *64*, *73*, *75*, *82*).

In the mouse oviduct, *Lgr5+* was proposed as a marker (*64*), while a recent report suggested that stem-like epithelial cells are marked by *Slc1a3* (*59*). In the human FTs, one early proposed candidate for the progenitor cell types was “peg” cells, characterized by EpCAM^+^, CD44^+^, and ITGA6^high^ as they resembled small round wooden pegs driven between the cells on the basis of their morphology (*61*). “Peg” cells have been long described in the FTs. The literature has also used the phrase “intercalated” cells along with “peg” cells, as these cells were found between ciliated and secretory cells (*81*). Their function and role within the FTE were unclear. Some argued that they merely represent a variation of nonciliated cells (*83*) or, more specifically, emptied secretory cells (*84*).

Recent scRNA-seq work proposed different models for the human FTs. One study suggested a model in which secretory cells dedifferentiate into a *RUNX3*^*+*^ intermediate population, which then differentiates into ciliated cells (*75*), while others (*61*, *73*) revealed multiple populations of progenitor cell types within the FT that may give rise to the observed differentiated cell identities within the epithelial, mesenchymal, and endothelial compartments. While no full consensus has emerged among the studies, two studies (*73*, *82*) both proposed progenitor cell types that simultaneously express epithelial (*EPCAM*) and endothelial (*CD31*/*PECAM1*) markers, capable of differentiating both epithelial and endothelial cells within the FT. A divergence is whether these cells give rise to both ciliated and secretory cells (*82*) or ciliated cells only (*73*) in the epithelial compartment.

Similar progenitor/stem–like “dual feature” cells with multipotent differentiation capacity for tissue regeneration and repair exist in the developing embryo and liver, colorectal, and breast tissues (*85*–*87*). Moreover, the epithelial-to-mesenchymal transition and mesenchymal-to-epithelial transition are phenomena where cells of a given lineage compartment can transiently express other cell identity features during cancer progression and tissue repair. While these cell types have been identified across multiple FT studies and scRNA-seq technologies, they have not yet shown differentiation capacity in vitro beyond inference of lineage fate from single-cell transcriptional studies, combined with cytometry- and imaging-based studies to corroborate their presence within primary tissue (*82*). Hence, we seek to refine the existing cell identity, ontology, and nomenclature to harmonize the existing literature defining observed cell populations found in the human FT. We have created the use of “unclassified fallopian tube progenitor cell” (http://purl.obolibrary.org/obo/CL_4052070) to encompass cells with the capacity to differentiate into epithelial and/or endothelial stromal lineages. In the future, subtypes of this cell type to denote the relevant tissue compartment, such as epithelial, mesenchymal, and endothelial, might be added.

## UTERUS

The endometrium, the inner lining of the uterus (Fig. 6 and figs. S6 and S7), is a highly dynamic tissue that regenerates hundreds of times throughout the female reproductive years during the menstrual cycle. Structurally, the endometrium is divided into two main layers: the stratum functionalis (functionalis) and the stratum basalis (basalis). The functionalis, which is adjacent to the uterine cavity, undergoes cyclical changes through the menstrual cycle (*88*, *89*). The underlying basalis layer serves as a regenerative reservoir, largely maintaining its structure throughout the menstrual cycle. The uterine menstrual cycle is classically divided into three phases that change the composition of the functionalis endometrium: the proliferative phase (ovarian counterpart is the follicular phase), where the functionalis thickens under estrogen influence; the secretory phase (ovarian counterpart is the luteal phase), where it differentiates driven by progesterone after ovulation; and the shedding phase (menstruation) driven by progesterone withdrawal if embryo implantation does not occur (*90*, *91*). Besides ovarian hormones, the structure and function of the endometrium are also shaped by changes in the paracrine factors produced locally by its various cell types, all working in concert to maintain tissue homeostasis and reproductive capacity. The updated cell type hierarchy for the uterus is summarized in Fig. 7, and marker genes for each cell type are listed in Table 3.

**Fig. 6.**
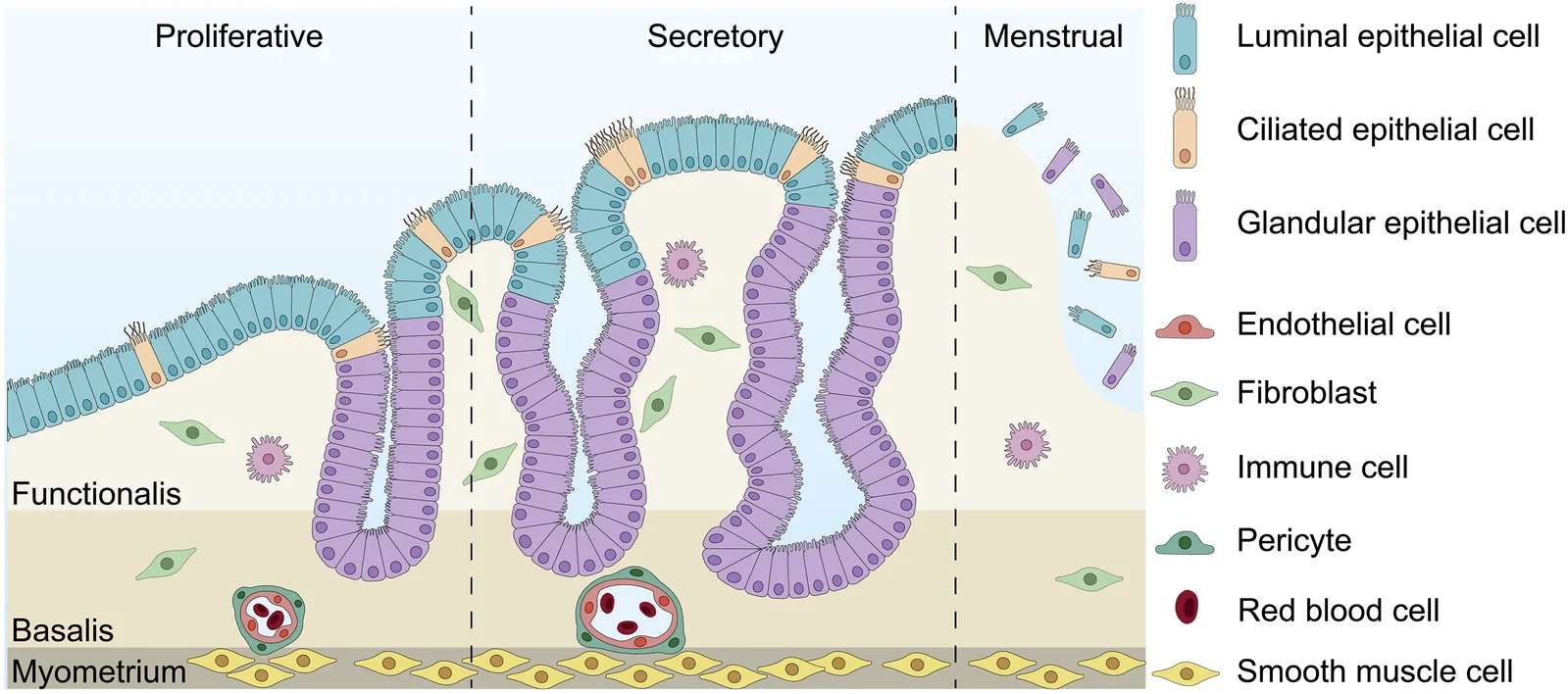
Illustration of the uterus. Overview of the uterus during proliferative, secretory, and menstrual stages and the cell types that are present. Figure created by L. S. E. Kim using Adobe Illustrator (Adobe Inc., San Jose, California).

**Fig. 7.**
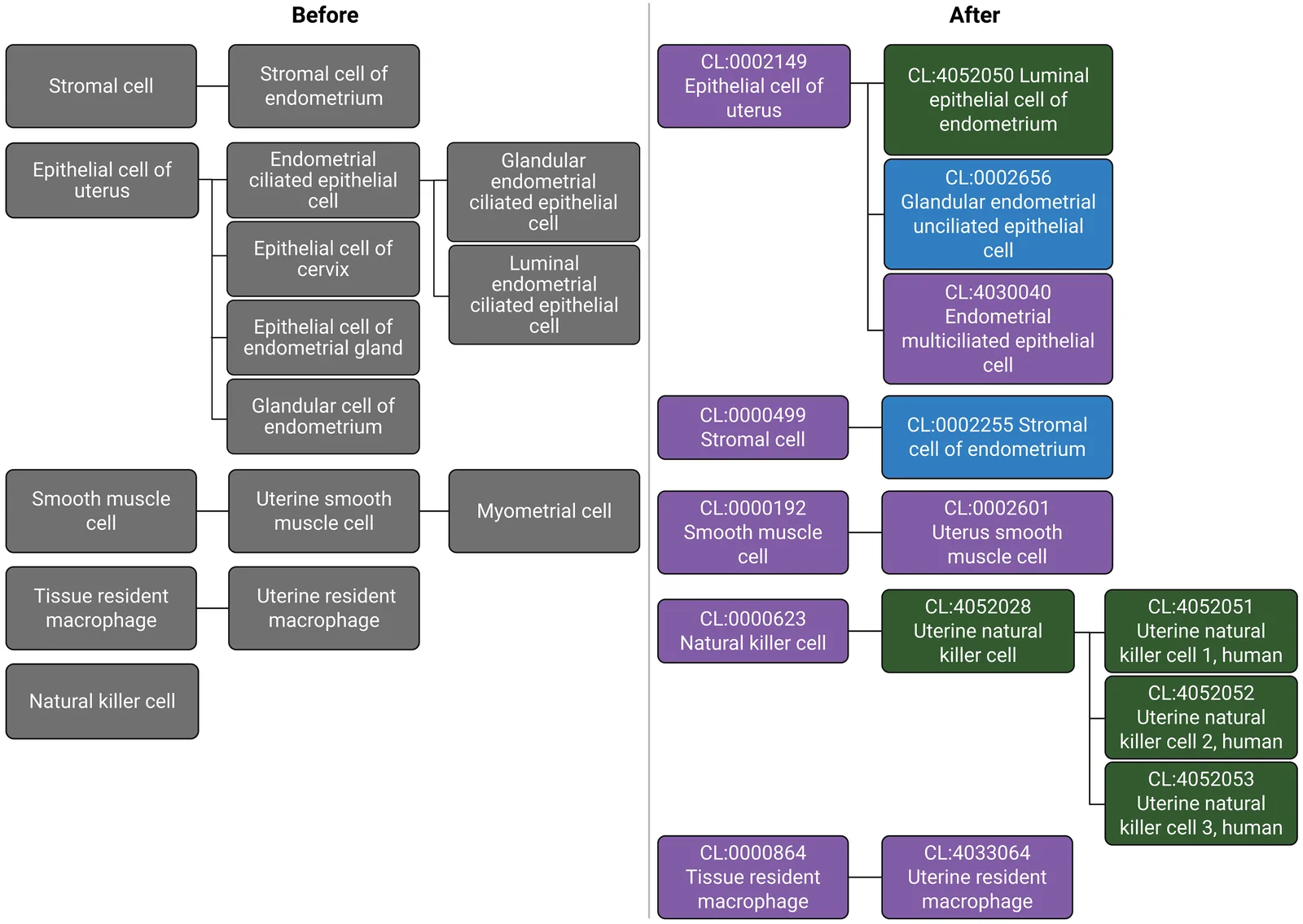
Updates to uterus Cell Ontology. Updates to the cell type hierarchies of the uterus from before (left) to after (right) in the Cell Ontology following the jamboree. Changes to FT cell classifications include newly added terms (green), revised or reclassified terms (blue), and preexisting terms (purple). Created in BioRender. H. T. Ong (2026), https://biorender.com/v3n8b84.

The endometrial glands are a crucial functional unit of the tissue, extending to the surface from the basalis layer (*89*). The basalis layer of the glands houses epithelial progenitor cells, which play a vital role in regenerating the epithelium after menstruation (*92*, *93*). The glands secrete essential nutrients and signaling molecules that create a favorable environment for embryo implantation, underscoring their dual role in both tissue renewal and pregnancy preparation.

At the core of the endometrium’s complex structure is the mesenchymal-epithelial cross-talk, which aids the endometrium’s cyclical remodeling. This interaction is an evolutionarily conserved and fundamental organizational principle in many barrier tissues (*94*), including the skin and intestinal lining (*95*). Mesenchymal cells (e.g., fibroblasts) provide structural support and communicate with epithelial cells to regulate tissue renewal. This dynamic cooperation, responsive to cyclical changes of ovarian hormones, is essential for maintaining the integrity and function of the endometrial tissue. The ECM also plays a vital role in these processes. Acting as both a scaffold and signaling platform, it mediates communication between different cell types, guiding cell behavior and promoting regeneration.

Endometrial tissue samples are typically obtained via the insertion of a suction tube into the uterine cavity. The specific region sampled substantially influences the insights obtained. Most biopsies target the functionalis layer, which is informative because of its cyclical changes during the menstrual cycle. However, this approach often misses the epithelial progenitor cells located in the basalis glands, essential for endometrial regeneration (*96*–*98*). Moreover, the use of exogenous hormones, such as in contraceptives or hormone replacement therapy, can alter the expected histological features and cell type composition at specific stages of the natural menstrual cycle. Accurate analysis and interpretation of endometrial biopsy data thus require consideration of both technical factors (e.g., sampling technique or tissue treatment protocols) and biological variables (e.g., menstrual phase or hormonal treatments).

Understanding the composition and coordinated communication between the various cell types in the endometrium is crucial for appreciating its cyclical regenerative capacity and role in reproduction. The dynamic interplay between epithelial, mesenchymal, smooth muscle, vascular, and immune cells, together with the ECM, orchestrates preparation for potential pregnancy or, alternatively, tissue regeneration after menstruation. These processes, finely tuned by hormonal signals, depend on precise regulation of cellular behavior and communication across endometrial layers. In the following section, we review these cell populations in detail, highlighting how our knowledge has evolved from early histological studies to modern single-cell analyses and providing a unifying nomenclature that can be used by the scientific community moving forward. As previously mentioned, this review focuses solely on the nonpregnant uterus during reproductive age, as no integrative effort has yet been made to assess cell type diversity in the pregnant uterus.

### Epithelial cell of the uterus

Endometrial epithelial cells have traditionally been classified according to their location within the uterine tissue. Luminal epithelial cells form a continuous layer lining the uterine cavity, serving as the initial interface for embryo implantation. Deeper within the endometrium, glandular epithelial cells organize into tubular structures, producing and secreting crucial nutrients and signaling molecules. Early microscopic examinations also revealed the presence of CE cells interspersed among both luminal and glandular epithelial cells (*99*, *100*), playing a key role in fluid movement within the uterine environment.

Research in the late 20th century elucidated the complex hormonal regulation of endometrial epithelial populations. During the proliferative phase of the menstrual cycle, elevated estrogen levels stimulate mitotic activity and DNA synthesis in glandular epithelial cells, which exhibit peak expression of estrogen receptor α (ERα) and ERβ during this phase (*101*–*103*). Concurrently, estrogen action induces progesterone receptor (PGR) expression in the glandular epithelium. Postovulation, progesterone antagonizes estrogen-mediated epithelial proliferation, shifting the glandular epithelial phenotype toward glycogen production, as evidenced by the appearance of subnuclear vacuoles (*91*). This transition is accompanied by a decrease in epithelial PGR expression during the secretory phase. Further insights emerged from knockout mouse studies and tissue recombination models, which highlighted the critical role of mesenchymal-epithelial interactions in regulating epithelial cell growth and PGR expression (*104*). Notably, estrogen signaling via ERα in mesenchymal cells was found to down-regulate PGR expression in epithelial cells, underscoring the importance of mesenchymal-derived signals in modulating epithelial behavior.

To address inconsistencies in cell type naming across different studies and standardize nomenclature, the Human Endometrial Cell Atlas (HECA) was created. The HECA integrates data from seven scRNA-seq datasets (*96*–*98*, *105*–*108*) and maps the identified cell types onto tissue architecture via spatial transcriptomics, providing a comprehensive framework for understanding endometrial cell types (*107*).

### Luminal epithelial cells

According to the HECA, luminal epithelial cells include three key states: *SOX9^+^* luminal, preluminal, and secretory luminal. *SOX9^+^* luminal cells are detected during the proliferative phase and are characterized by up-regulation of noncanonical WNT signaling mediator *WNT7A* (as inferred by lack of *AXIN2* signaling in these cells) (*109*, *110*) and *LGR5* (*111*)*.* As the cycle progresses into the early secretory phase, preluminal cells emerge, expressing genes such as *VTCN1* and *SULT1E1*, with the latter involved in estrogen catabolism (*112*). By the mid- and late-secretory phases, these cells differentiate into secretory luminal cells, characterized by the expression of *LEFTY1* [a TGFβ (transforming growth factor–β) signaling inhibitor] (*113*), *IL6* (an inflammatory cytokine) (*114*), and *PTGS1* (involved in prostaglandin synthesis) (*115*). *LGR5* expression is also heightened in these cells. These factors work in concert to create a receptive environment for embryo implantation.

### Glandular epithelial cells

Glandular epithelial cells exhibit a more complex hierarchy of states, which can be broadly categorized into *SOX9^+^* glandular cells (further subdivided into *SOX9^+^* glandular basalis, *SOX9^+^* glandular functionalis I, and *SOX9*^+^ glandular functionalis II), preglandular, glandular, and glandular secretory cells. *SOX9^+^* glandular basalis cells, located in the basalis layer, persist throughout both proliferative and secretory phases of the menstrual cycle. The expression of *AXIN2*, a direct target of β-catenin–dependent canonical Wnt signaling (*116*), suggests active WNT/β-catenin signaling in maintaining these putative stem/progenitor cells, similar to stem cell niches in other tissues such as the small intestine (*117*) and developing lung (*118*). During the proliferative phase, two distinct populations of *SOX9^+^* glandular cells emerge in the functionalis layer: *SOX9^+^* glandular functionalis I and functionalis II. Both populations show increased *IHH* expression compared to the *SOX9^+^* basalis cells, indicating a role for Hedgehog signaling in their differentiation (*119*). The *SOX9^+^* functionalis I population also expresses estrogen receptor signaling targets like *PHLDA1*, reflecting the response to estrogen during the proliferative phase (*120*).

As the menstrual cycle progresses into the secretory phase, preglandular cells become evident. These cells are marked by the down-regulation of Hedgehog signaling [e.g., *SUFU* (*121*, *122*)] and the establishment of apico-basal polarity [*CRB3* (*123*)]. They subsequently mature into mid-secretory glandular cells, distinguished by the enriched expression of genes such as *SCGB2A2* and *ABCG1*, which are involved in glandular structure formation (*124*) and lipid transport (*125*), respectively.

In the mid-secretory phase, a population of glandular secretory cells emerges, expressing *PAEP* (glycodelin), *DPP4*, and *GPX3*. These genes are crucial for creating a supportive environment for potential embryo implantation and are characteristic of the receptive endometrium (*126*, *127*). Notably, *PAEP* is considered a hallmark of endometrial receptivity, while *DPP4* and *GPX3* are part of a gene set used to estimate the menstrual cycle stage (*128*).

### Ciliated epithelial cells

CE cells, which are present in the uterus of humans but not in mice (*129*), exist in two primary states: preciliated and ciliated. Preciliated cells emerge during the proliferative phase and are crucial for centriole amplification and deuterosome formation and may contribute to epithelial barrier formation and host-microbiota interactions. As the cycle progresses into the secretory phase, preciliated cells differentiate into fully ciliated cells.

Previously, the CL did not adequately separate luminal, glandular, and ciliated cells, making it difficult to map high-resolution datasets without losing spatial and functional metadata. To resolve this, CL now defines “epithelial cell of uterus” (http://purl.obolibrary.org/obo/CL_0002149) as the parent class for three subclasses: the newly added term “luminal epithelial cell of endometrium” (http://purl.obolibrary.org/obo/CL_4052050), the modified term “glandular endometrial unciliated epithelial cell” (http://purl.obolibrary.org/obo/CL_0002656), and “endometrial multiciliated epithelial cell” (http://purl.obolibrary.org/obo/CL_4030040). This update aligns the CL structure with the major epithelial lineages identified in the HECA, and the hierarchical framework established here allows new subtypes to be incorporated as emerging spatial and single-cell data continue to resolve finer cell states within these lineages.

## REFINING THE STROMA

The stroma of reproductive tissues plays critical roles in supporting tissue homeostasis and function. The stroma contains a diverse cell population including fibroblasts, endothelial cells, smooth muscle cells, pericytes, and immune cells; however, a major issue in current studies is the tendency to label diverse stroma populations under the broad term “stroma” without fully annotating and distinguishing them by specific cell types or states. This oversimplification obscures the complexity and function of these cells, especially in tissues like the ovary, FT, and uterus, where stromal cell composition changes dynamically throughout the menstrual cycle and reproductive life span.

In the ovary, stromal fibroblasts and immune cells exhibit distinct roles and shifting populations throughout folliculogenesis and the reproductive life span yet are often lumped together in datasets. The same issue arises in the uterus, where mesenchymal stromal cells undergo decidualization, and in the FT, where distinct cell types supporting tissue function remain poorly characterized. Without proper annotation, critical insights into how these populations contribute to tissue function in health and disease, such as endometriosis or ovarian cancer, are lost and tasks such as downstream integration are hindered.

In addition, stromal cell populations can differ in their sensitivity to standard single-cell processing workflows, which should be considered when interpreting dissociation-based datasets. Many stromal cell types, including fibroblasts and immune cells, are responsive to mechanical cues such as stiffness, stretch, compression, and shear stress, all of which are intrinsic features of the FRT microenvironment (*25*, *130*, *131*). Enzymatic digestion of the ECM and subsequent handling steps required for single-cell isolation may therefore influence cell viability or transient transcriptional states in a cell type–dependent manner. While single-cell transcriptomic approaches have been instrumental in defining stromal heterogeneity, these considerations highlight the value of integrating complementary approaches, including spatial transcriptomics and minimally perturbative methods, to contextualize stromal cell identities and states within their native tissue environment.

Below is an ontology proposal for the stromal cell populations, which requires cell type–level annotation. It should be noted that this is far from a comprehensive summary of all stromal cells across the ovary, FT, and uterus. Rather, this is an effort to highlight the need to annotate these cell types at a finer resolution to provide new insights into the diversity and dynamics of how these cells support tissue function and how their dysregulation leads to reproductive diseases such as endometriosis, infertility, and ovarian cancer. While the resolution offered by omics technologies (e.g., transcriptomics, proteomics, and spatial) continues to rapidly expand and grow our knowledge of the cells that make up these critical organs, we must improve the annotation and characterization of these diverse stromal cell populations to fully understand their contributions to both health and disease. Proposed markers for each stromal population are provided (table S1).

### Fibroblasts

The heterogeneity of fibroblasts and their role in maintaining tissue homeostasis through various cell states represent some of the most intricate challenges to address. As the predominant cell type within the connective tissue, fibroblasts are responsible for synthesizing ECM components in response to diverse stimuli. In addition, they facilitate tissue remodeling by degrading the ECM via enzymes such as matrix metalloproteinases (MMPs) (*132*). During mammalian development, fibroblasts originate from the primary mesenchyme during gastrulation (*133*). An accumulation of fibroblasts, particularly activated fibroblasts (also known as myofibroblasts), marked by increased expression of α-smooth muscle actin (*134*), is frequently linked to pathological conditions, including fibrosis (*135*, *136*), wound healing (*137*), tumor progression (*138*, *139*), and the aging process (*140*). To add to the complexity, various cell types in other tissues have been shown to trans-differentiate into myofibroblasts, including monocytes (*141*), macrophages (*142*), pericytes (*143*), smooth muscle cells (*144*), among others.

In the FT, fibroblasts produce growth factors essential for normal preimplantation embryo development (*145*). In the uterus, fibroblasts undergo decidualization during the menstrual cycle, transforming into secretory cells essential for implantation. In the aging ovary, the presence of excess ECM has been suggested to be linked to the role of stromal fibroblasts contributing to fibrosis and stiffening of the ovary, which may interfere with follicle development, hinder follicular rupture, and affect overall fertility (*146*–*148*).

### Endothelial cells

Endothelial cells are essential for vascular function, angiogenesis, and immune cell trafficking in reproductive tissues. In the ovary, they facilitate follicular development, ovulation, and corpus luteum formation. Dysregulation of angiogenic factors and overall dysfunction of endothelial cells are linked to conditions such as polycystic ovarian syndrome, ovarian hyperstimulation syndrome, subfertility, premature ovarian insufficiency, and aging (*148*, *149*). In the FT, endothelial cells support the vascularization necessary for epithelial and stromal functions involved in gamete and embryo transport. In the uterus, endothelial cells regulate vascularization of the endometrium during the menstrual cycle and pregnancy, with aberrations contributing to recurrent pregnancy loss and endometriosis. Along the same lines, lymphatic endothelial cells form lymphatic vessels and mediate communication between the FRT and draining lymph nodes, contributing to local immune regulation, tissue homeostasis, hormone transport, and nutrient delivery.

### Pericytes

Pericytes are supportive cells that regulate blood vessel stability, permeability, and angiogenesis by interacting with endothelial cells. In the ovary, pericytes assist in the dynamic vascular remodeling that occurs during follicular development, ovulation, and corpus luteum formation (*150*). In the FT, pericytes contribute to maintaining the integrity of the tubal vasculature, crucial for nutrient delivery to the tube’s epithelial and stromal compartments (*61*, *151*). In the uterus, pericytes play a role in the extensive vascular changes associated with the menstrual cycle and pregnancy, and their dysfunction may contribute to pathologies such as endometriosis, uterine fibroids, and recurrent pregnancy loss (*152*).

### Smooth muscle cells

Smooth muscle cells play a key role in the contractility of reproductive tissues. In the ovary, they are located primarily in the outer layer of the follicle in the theca externa, and they are involved in follicle rupture during ovulation, where coordinated contraction and vascular changes lead to the release of the egg (*150*, *153*). In the FT, smooth muscle cells are critical for sperm transport (*154*) and propelling the oocyte and embryo through the tube via peristaltic contractions. In the uterus, smooth muscle cells, which form the myometrium, are essential for uterine contractions during sperm transport, menstruation, and labor. Dysregulation of smooth muscle cell function in these tissues can lead to conditions such as infertility and smooth muscle metaplasia (*155*, *156*).

### Immune cells

Both resident and recruited immune cells play a critical role in ovarian, uterine, and FT function (see tables S1 and S2 for more detailed CL terms). Among many other functions, immune cells clear apoptotic follicles and regulate ovulation-related inflammation, with chronic immune dysregulation contributing to ovarian pathologies like cancer (*157*) and premature ovarian insufficiency (*158*, *159*). In the aging ovary, immune remodeling includes the emergence of multinucleated giant cells, which arise from macrophage fusion and an increase in abundance with age in both mouse and nonhuman primate ovaries. Because of their large, multinucleated structure, multinucleated giant cells have only recently been characterized using spatial imaging and laser capture microdissection approaches (*160*). In the FT, immune cells, including macrophages, dendritic cells, and lymphocytes, play a vital role in local immune surveillance, pathogen clearance, and tissue repair (*161*–*163*). Disruptions of immune cells in the FT lead to inflammatory conditions such as pelvic inflammatory disease associated with infertility, increased risk of ectopic pregnancy, and cancer (*161*, *164*, *165*). In addition, imbalances in the immune microenvironment in the FT, characterized by immunosuppressive tumor-associated macrophages and regulatory T cells (*163*) and myeloid cell alterations (*161*, *166*), may promote malignant transformation and progression. In the uterus, immune cells modulate inflammation and support immune tolerance during pregnancy, with abnormalities implicated in endometriosis. Single-cell transcriptomic analyses have identified three major subsets of uterine natural killer (uNK) cells in early pregnancy (uNK1, uNK2, and uNK3), each with distinct receptor repertoires and functional profiles (*167*). These subsets were subsequently confirmed and contextualized within the integrated cellular atlas of the nonpregnant endometrium, suggesting that similar uNK cell populations persist across reproductive states (*107*). To reflect this, “uterine natural kill cell” (http://purl.obolibrary.org/obo/CL_4052028) is added as a parent term for “uterine natural kill cell 1, human” (http://purl.obolibrary.org/obo/CL_4052051), “uterine natural kill cell 2, human” (http://purl.obolibrary.org/obo/CL_4052052), and “uterine natural kill cell 3, human” (http://purl.obolibrary.org/obo/CL_4052053) in the Cell Ontology.

### Peripheral nerves and glial cells

Neural innervation and associated glial cells are integral but often underappreciated components of the ovarian microenvironment. Sympathetic innervation of the ovary was first described in adult mammals in the early 1980s (*168*), with neural crest–derived fibers arising from the celiac ganglion and entering through the hilum to innervate the theca layer, interstitial glands, and ovarian vasculature. Subsequent developmental studies in mice demonstrated that sympathetic nerves migrate posteriorly from the celiac ganglion through the mesonephros to contact the dorsal ovary by embryonic day 16.5, form a dorsal plexus before birth, and densely innervate the dorsal medulla by postnatal day 5 development (*169*). Recent comparative analyses indicate that human fetal ovaries follow a substantially similar temporal and spatial program of sympathetic patterning that persists into adulthood (*17*).

Alongside neuronal inputs, peripheral glial cells are now recognized as key components of the ovarian stroma. Developmental studies in mice (*169*) and recent single-cell and spatial analyses (*17*) have identified ovarian glial populations in both mouse and human ovaries. As sympathetic neuronal cell bodies reside in the celiac ganglion, they are not captured by isolated ovarian datasets; however, their supporting glial cells are resident within the ovary and can be detected transcriptionally. Notably, glial cells are highly sensitive to enzymatic dissociation, which likely contributes to their underrepresentation or absence in many published ovarian single-cell atlases despite robust detection by immunostaining. Approaches that preserve spatial context, including spatial transcriptomics or single-nucleus sequencing, may therefore provide improved strategies for characterizing glial transcriptomes in situ.

Reported markers for human ovarian glia include *FOXD3*, *MYOT*, *CDH19*, *PLP1*, *S100B*, *SOX10*, *L1CAM*, *SFRP5*, *ERBB3*, and *MPZ*, whereas murine ovarian glia have been described using markers such as *Kcna1*, *Sox10*, *Rxrg*, *Sfrp5*, *Serpind1*, *Cdh19*, *Cadm2*, *Gfra2*, *L1cam*, and *S100b*. Beyond the ovary, the oviduct and uterus also receive dense sympathetic and sensory innervation that regulates smooth muscle contractility, epithelial transport, and vascular tone (*170*), although glial populations in these tissues have not yet been characterized to date. Incorporating neural and glial populations into ovarian and reproductive tract cell ontologies will be essential for accurately representing the neurovascular and neuroendocrine interactions that shape folliculogenesis, tissue homeostasis, aging, and disease.

## TOWARD AN INTEGRATED FEMALE REPRODUCTIVE ATLAS

We aim for the revised Cell Ontology terms presented here to facilitate improved annotation of existing single-cell datasets and streamline the contribution of forthcoming data. While integration of existing datasets and reannotation with the revised Cell Ontology terms were outside of the scope of this meeting and the summary within this review, this work provides a foundation for future efforts that would improve the biological understanding of the FRT. Integration efforts should first focus on creating atlases for the ovary, FT, and other organs in the same manner as the recent endeavor for the endometrium (*107*). Data providers and computational scientists should work iteratively to evaluate and select appropriate integration tools, apply stringent quality control filters, and clearly define criteria for arriving at consensus cell annotations and cell-specific marker genes. Ideally, these genes should be independently confirmed using an orthogonal method such as RNA in situ hybridization or spatial transcriptomics and be tested for concordance at the protein level by methods such as immunohistochemistry, Western blotting, or mass spectrometry. As a starting point, we have compiled a list of marker genes for cell types discussed (Tables 1 to 3 and table S1) and are in the process of incorporating both CL terms and marker genes into the Human Reference Atlas (table S2) (*171*–*173*) to facilitate their use. We encourage the community to explore these resources and to provide feedback for improvement (Tables 1 to 3 and table S1). Once organ-specific cell atlases are complete, all FRT tissues could then be aligned and/or integrated to assess overlap in cell annotations and phenotypic signatures, forming a basis for systems-level analyses and hypotheses to be formulated.

Future extensions of this ontology framework should prioritize dynamic physiological contexts including pregnancy, menopause, and development, as well as tissues not covered here such as the cervix, vagina, and external genitalia. Pathological settings including endometriosis, HGSC, and reproductive aging represent particularly important directions, as cell identity boundaries in these contexts may diverge from the baseline defined here in ways that are both biologically informative and clinically relevant. The stable reference established by this ontology will serve as the necessary comparator against which such deviations can be systematically identified and interpreted, making the present work a foundation for, rather than a limitation of, future translational and disease-focused efforts.

## Supplementary Materials

### This PDF file includes:

Supplementary Text Figs. S1 to S7 Legend for table S1 Table S2 References
