# Supplementary material for: Standardizing Cell Ontology terms for cross-study integration in the female reproductive tract
Source: Sci Adv. 2026 Oct 9;12(41):eaef2358. doi: 10.1126/sciadv.aef2358 (PMC13651846; doi:10.1126/sciadv.aef2358)
Supplement: Supplementary file 1 [file sciadv.aef2358_sm.pdf]

Science Advances
AAAS

Supplementary Materials for

Standardizing Cell Ontology terms for cross-study integration in the female reproductive tract

Huan Ting Ong *et al.*

Corresponding author: Huan Ting Ong, ong.ht@nus.edu.sg; Diane C. Saunders, diane.saunders@northwestern.edu; Ariella Shikanov, shikanov@umich.edu; Francesca Duncan, f-duncan@northwestern.edu; Hui Shen, hui.shen@vai.org; Jennifer L. Garrison, jennifer.garrison@ucsf.edu; Mariana Boroni, mariana.boroni@inca.gov.br; Michael B. Stout, michael-stout@omrf.org; Roser Vento-Tormo, rv4@sanger.ac.uk; Sophia H. L. George, sophia.george@med.miami.edu; Yousin Suh, ys3214@cumc.columbia.edu; Jennifer L. Young, biejly@nus.edu.sg; Monica M. Laronda, mlaronda@luriechildrens.org

Sci. Adv. **12**, eaef2358 (2026)
DOI: 10.1126/sciadv.aef2358

This PDF file includes:

Supplementary Text
Figs. S1 to S7
Legend for table S1
Table S2
References

Other Supplementary Material for this manuscript includes the following:

Table S1

Supplementary Text 1: CL Ontology updates for Female Reproductive System

Complete OWL axioms, equivalence axioms, SubClassOf axioms, synonym provenance, distinguishing criteria between sibling terms, and external ontology cross-references for all new and modified Cell Ontology terms across the ovary, fallopian tube, and uterus.

Overview

This document provides formal OWL axioms, logical definitions, and relationship mappings for all Cell Ontology (CL) terms that were added or significantly modified as part of the female reproductive system curation effort. All terms have been added to the Cell Ontology repository (https://github.com/obophenotype/cell-ontology) with stable identifiers and are available in the latest CL release. This document was created with the assistance of AI agents integrated into CL. Changes are organized by anatomical region: ovary (Section 1), fallopian tube (Section 2), and uterus (Section 3). For each term, we provide:

- **Term IRI and label**
- **Textual definition** with literature references
- **OWL logical axioms**: equivalence axioms (necessary and sufficient conditions) and SubClassOf axioms (necessary conditions), including named parent classes (is_a) and relationship axioms to external ontologies
- **Developmental relationships** (where applicable)
- **Synonyms** with provenance

Prefix Declarations

Prefix	Namespace
obo:	http://purl.obolibrary.org/obo/
CL:	http://purl.obolibrary.org/obo/CL_
UBERON:	http://purl.obolibrary.org/obo/UBERON_
GO:	http://purl.obolibrary.org/obo/GO_
PATO:	http://purl.obolibrary.org/obo/PATO_
PR:	http://purl.obolibrary.org/obo/PR_
NCBITaxon:	http://purl.obolibrary.org/obo/NCBITaxon_

Relationship Properties Used

Property	Label
BFO:0000050	`part_of`
RO:0000053	`has_characteristic`
RO:0000056	`participates_in`
RO:0002104	`has_plasma_membrane_part`

Property	Label
RO:0002162	`in_taxon`
RO:0002202	`develops_from`
RO:0002203	`develops_into`
RO:0002207	`directly_develops_from`
RO:0002210	`directly_develops_into`
RO:0002215	`capable_of`
RO:0002388	`has_direct_input`
CL:4030045	`lacks_part`
CL:4030046	`lacks_plasma_membrane_part`

1. Ovary

1.1 Granulosa Cell Lineage

The granulosa cell hierarchy was significantly restructured. The existing `granulosa cell` (CL:0000501) now has four new subtypes representing distinct developmental stages, plus new terms for atretic states. The developmental trajectory is:

```
pre-granulosa cell → squamous granulosa cell → cuboidal granulosa cell → { mural granulosa cell, cumulus granulosa cell }
```

1.1.1 pre-granulosa cell (CL:4033066) — NEW

Definition: A supporting cell that is part of the ovary and differentiates into a granulosa cell. A pre-granulosa cell develops from an early supporting gonadal cell by repressing testis determination, which can then proliferate to form primordial follicles. [PMID:17639032, PMID:32759216, PMID:33970370, PMID:38178246]

Logical Axioms:

```
SubClassOf(CL:4033066, CL:0000630)                          # is_a supporting cell
SubClassOf(CL:4033066, part_of some UBERON:0000992)          # part_of ovary
SubClassOf(CL:4033066, directly_develops_into some CL:4033083) # directly_develops_into squamous granulosa cell
```

Inferred parent (via develops_into): granulosa cell (CL:0000501) — `develops_from some pre-granulosa cell (CL:4033066)`

Synonyms:

- pregranulosa cell (exact) [PMID:17639032, PMID:32759216]
- ovarian pregranulosa cell (exact) [PMID:32759216]
- PG cell (related, abbreviation) [PMID:32759216]

Distinguishing criteria: Unlike granulosa cells, pre-granulosa cells are found prior to follicle formation and are classified as supporting cells rather than follicular cells of the ovary.

1.1.2 squamous granulosa cell (CL:4033083) — NEW

Definition: A granulosa cell that has a squamous morphology and forms a single layer around the oocyte in primordial follicles. This cell develops directly into a cuboidal granulosa cell during the primordial-to-primary follicle transition. [PMID:28892263]

Logical Axioms:

```
SubClassOf(CL:4033083, CL:0000501)                              # is_a granulosa cell
SubClassOf(CL:4033083, part_of some UBERON:0003981)              # part_of primordial ovarian follicle
SubClassOf(CL:4033083, has_characteristic some PATO:0002254)     # has_characteristic squamous
SubClassOf(CL:4033083, directly_develops_from some CL:4033066)   # directly_develops_from pre-granulosa cell
SubClassOf(CL:4033083, directly_develops_into some CL:4033084)   # directly_develops_into cuboidal granulosa cell
```

Synonyms:

- flattened granulosa cell (exact) [PMID:22402964]
- pre-granulosa cell (related) [PMID:33914868]
- squamous GC (related, abbreviation) [PMID:33914868]

Distinguishing criteria from cuboidal granulosa cell: Characterized by squamous (flat) morphology (PATO:0002254) and location in the primordial follicle (UBERON:0003981), versus cuboidal morphology and location in the ovarian follicle.

1.1.3 cuboidal granulosa cell (CL:4033084) — NEW

Definition: A granulosa cell that has a cuboidal morphology and develops from squamous granulosa cell during the transition between primordial follicle to primary follicle. Cuboidal granulosa cells proliferate to form a second layer within secondary follicles. [PMID:28892263]

Logical Axioms:

```
SubClassOf(CL:4033084, CL:0000501)                              # is_a granulosa cell
SubClassOf(CL:4033084, part_of some UBERON:0001305)              # part_of ovarian follicle
SubClassOf(CL:4033084, has_characteristic some PATO:0001872)     # has_characteristic cuboidal
SubClassOf(CL:4033084, directly_develops_from some CL:4033083)   # directly_develops_from squamous granulosa cell
```

Develops into (asserted on children):

- mural granulosa cell (CL:4033067) — `develops_from some CL:4033084`
- cumulus granulosa cell (CL:0000711) — `develops_from some CL:4033084`

Synonyms:

- columnar granulosa cell (exact) [PMID:19001500]
- cuboidal epithelial granulosa cell (exact) [PMID:31849844]
- columnar GC (related, abbreviation) [PMID:19001500]
- cuboidal GC (related, abbreviation) [PMID:33914868]

Distinguishing criteria from squamous granulosa cell: Cuboidal morphology (PATO:0001872) and broader location in the ovarian follicle (UBERON:0001305) versus squamous morphology in primordial follicles only.

1.1.4 mural granulosa cell (CL:4033067) — NEW

Definition: A follicular cell of ovary that differentiates from a cuboidal granulosa cell during the secondary follicle stage. Mural granulosa cells line the inner surface of the follicle wall, surrounding the fluid-filled antral cavity. These cells produce oestrogen during the follicular phase in response to follicle-stimulating hormone (FSH), and progesterone after ovulation in response to luteinizing hormone (LH). [PMID:27815559, PMID:33970370, PMID:34198768]

Logical Axioms:

```
SubClassOf(CL:4033067, CL:0000501)                          # is_a granulosa cell
SubClassOf(CL:4033067, part_of some UBERON:0000992)          # part_of ovary
SubClassOf(CL:4033067, develops_from some CL:4033084)        # develops_from cuboidal granulosa cell
```

Cross-references: EFO:0005289

Synonyms:

- MGC (related, abbreviation) [doi:10.1530/REP-16-0500]
- mural GC (related, abbreviation) [PMID:34198768]

Distinguishing criteria from cumulus granulosa cell (CL:0000711): Mural granulosa cells line the follicle wall, whereas cumulus granulosa cells surround and nourish the oocyte directly. Both develop from cuboidal granulosa cells.

1.1.5 cumulus granulosa cell (CL:0000711) — RENAMED

Previous label: cumulus cell

Definition: Cumulus cell is a specialized granulosa cell that surrounds and nourishes the oocyte. This cell-type surrounds the fully-grown oocyte to form a cumulus-oocyte complex (abbr. COC). [GOC:tfm]

Logical Axioms:

```
SubClassOf(CL:0000711, CL:0000154)                          # is_a protein secreting cell
SubClassOf(CL:0000711, CL:0000501)                          # is_a granulosa cell
SubClassOf(CL:0000711, develops_from some CL:4033084)        # develops_from cuboidal granulosa cell
```

Rationale for rename: Renamed from "cumulus cell" to "cumulus granulosa cell" for consistency with other granulosa cell subtype naming conventions (mural granulosa cell, squamous granulosa cell, cuboidal granulosa cell). The previous label is retained as an exact synonym.

1.1.6 atretic follicular cell of ovary (CL:4033089) — NEW

Definition: A follicular cell of ovary that has begun to degenerate and undergo atresia, a specialized apoptosis. This cell is found in an atretic follicle, which is an ovarian follicle that started to mature but failed to reach full development and instead regresses. [PMID:38069168]

Logical Axioms (Equivalence — necessary and sufficient conditions):

```
EquivalentClasses(CL:4033089,
    follicular cell of ovary (CL:0002174)
```

```
AND participates_in some ovarian follicle atresia (GO:0001552))
```

Children:

- atretic granulosa cell (CL:4033090)
- atretic theca cell (CL:4052027)

Distinguishing criteria: Defined by the intersection of being a follicular cell of the ovary AND participating in the ovarian follicle atresia process. This equivalence axiom provides machine-computable necessary and sufficient conditions.

1.1.7 atretic granulosa cell (CL:4033090) — NEW

Definition: A granulosa cell that is undergoing atresia. This cell type displays distinct morphological alterations compared with a healthy granulosa cell, including pyknosis (nuclear condensation) and cellular shrinkage. [PMID:15353131, PMID:38069168]

Logical Axioms:

```
SubClassOf(CL:4033090, CL:4033089)                          # is_a atretic follicular cell of ovary
SubClassOf(CL:4033090, develops_from some CL:0000501)        # develops_from granulosa cell
```

Synonyms:

- apoptotic granulosa cell (related) [PMID:15353131]

Distinguishing criteria from atretic theca cell (CL:4052027): Derives from granulosa cells (within the follicle) and shows pyknosis/shrinkage, whereas atretic theca cells derive from theca cells (surrounding the follicle) and show hypertrophy.

1.2 Theca Cell Lineage

The theca cell hierarchy was expanded with a precursor cell and two anatomically-defined subtypes:

```
pre-theca cell → theca cell → { interna theca cell, externa theca cell }
```

1.2.1 pre-theca cell (CL:4052010) — NEW

Definition: A stromal cell that serves as a precursor to the theca cell layers in the ovary growing follicles. This cell is present in the early stages of follicular growth, particularly in smaller follicles. Unlike mature theca cell, a pre-theca cell is initially non-steroidogenic and lacks luteinizing hormone receptors. [PMID:20628033, PMID:31320652, PMID:36205477]

Logical Axioms:

```
SubClassOf(CL:4052010, CL:0000499)                          # is_a stromal cell
SubClassOf(CL:4052010, part_of some UBERON:0012186)          # part_of ovarian cortical stroma
SubClassOf(CL:4052010, develops_into some CL:0000503)        # develops_into theca cell
```

Synonyms:

- early theca cell (exact) [PMID:36205477]
- progenitor theca cell (exact) [PMID:31320652]

Distinguishing criteria from theca cell (CL:0000503): Pre-theca cells are non-steroidogenic stromal cells lacking LH receptors, located in the cortical stroma. Theca cells are differentiated cells forming a layer around the follicle.

1.2.2 interna theca cell (CL:4052012) — NEW

Definition: A specialized theca cell that forms the inner, highly vascularized layer of the theca surrounding ovarian follicles. Originating from progenitor theca cells, the theca interna cell is steroidogenic, playing a crucial role in the production of androgens, which serves as a precursor for estrogen synthesis in granulosa cells. This cell expresses luteinizing hormone receptors, enabling it to respond to hormonal signals that regulate steroidogenesis. [PMID:15833266, PMID:31320652, PMID:32530882, Wikipedia:Theca_interna]

Logical Axioms (Equivalence — necessary and sufficient conditions):

```
EquivalentClasses(CL:4052012,
    theca cell (CL:0000503)
    AND part_of some theca interna (UBERON:0000157))
SubClassOf(CL:4052012, CL:0000593)                          # is_a androgen secreting cell
SubClassOf(CL:4052012, develops_from some CL:4052010)        # develops_from pre-theca cell
DisjointClasses(CL:4052012, CL:4052013)                     # disjoint with externa theca cell
```

Synonyms:

- androgenic theca cell (related) [PMID:36599970]
- inTC (related, abbreviation) [PMID:31320652]
- interna TC (related, abbreviation) [PMID:31320652]
- steroidogenic theca interna cell (related) [PMID:36599970]

Distinguishing criteria from externa theca cell (CL:4052013): Formally distinguished by anatomical location: part_of theca interna (UBERON:0000157) vs. part_of theca externa (UBERON:0000156). The two types are declared disjoint. Functionally, interna cells are steroidogenic (androgen-producing), while externa cells are structural (collagen-producing).

1.2.3 externa theca cell (CL:4052013) — NEW

Definition: A specialized theca cell that forms the outer layer of the theca surrounding the ovarian follicle, appearing at the antral follicle. Originating from progenitor theca cells, theca externa cell is characterized by its fibroblast-like appearance and primarily functions to provide structural support to the developing follicle. This cell produces collagen fibers and extracellular matrix components such as Col1a1 and Col1a2. [PMID:31320652, PMID:32530882, PMID:36758341, Wikipedia:Theca_externa]

Logical Axioms (Equivalence — necessary and sufficient conditions):

```
EquivalentClasses(CL:4052013,
    theca cell (CL:0000503)
    AND part_of some theca externa (UBERON:0000156))
SubClassOf(CL:4052013, develops_from some CL:4052010)        # develops_from pre-theca cell
DisjointClasses(CL:4052012, CL:4052013)                     # disjoint with interna theca cell
```

Synonyms:

- exTC (related, abbreviation) [PMID:31320652]
- externa TC (related, abbreviation) [PMID:31320652]
- structural theca cell (related) [PMID:36599970]

1.2.4 atretic theca cell (CL:4052027) — NEW

Definition: A theca cell undergoing atresia, characterized by distinct morphological changes including hypertrophy, altered cell shape, and disrupted layered organization. This cell exhibits reduced steroidogenic capacity and changes in surrounding vascularization. The onset of theca cell atresia can vary, occurring at different stages of follicle atresia depending on the phase of folliculogenesis. [PMID:20628033, PMID:38069168]

Logical Axioms:

```
SubClassOf(CL:4052027, CL:4033089)                          # is_a atretic follicular cell of ovary
SubClassOf(CL:4052027, develops_from some CL:0000503)        # develops_from theca cell
```

1.3 Theca Cell (CL:0000503) — MODIFIED (definition and hierarchy)

Updated definition: A specialized stromal cell that forms the theca layer outside the basal lamina lining the ovarian follicle, appearing during the secondary follicle stage. [MESH:D013799, PMID:15833266, PMID:36758341]

Logical Axioms:

```
SubClassOf(CL:0000503, CL:0000499)                          # is_a stromal cell
SubClassOf(CL:0000503, part_of some UBERON:0000155)          # part_of theca
```

New relationships to this term:

- pre-theca cell develops_into theca cell
- interna theca cell EquivalentTo theca cell AND part_of some theca interna
- externa theca cell EquivalentTo theca cell AND part_of some theca externa
- atretic theca cell develops_from theca cell

1.4 Luteal Cells

1.4.1 early luteal cell (CL:4052046) — NEW

Definition: A luteal cell that is part of the young, developing corpus luteum. This cell promotes progesterone synthesis, marked by high Parm1 expression in mice (Lan et al., 2024). An early luteal cell is associated with steroidogenesis and cell growth, contributing to early corpus luteum function and maturation. [PMID:36205477, doi:/10.1101/2024.12.15.628550]

Logical Axioms:

```
SubClassOf(CL:4052046, CL:0000175)                          # is_a luteal cell
SubClassOf(CL:4052046, capable_of some GO:0006701)           # capable_of progesterone biosynthetic process
```

Synonyms:

- active corpus luteum cell (exact) [PMID:36205477]

Distinguishing criteria from late luteal cell (CL:4052047): Early luteal cells are capable of progesterone biosynthesis (GO:0006701), whereas late luteal cells are capable of progesterone catabolic process (GO:0006709), reflecting their role in corpus luteum regression.

1.4.2 late luteal cell (CL:4052047) — NEW

Definition: A luteal cell that is part of the older, regressing corpus luteum. This cell exhibits increased expression of genes involved in progesterone metabolism - Akr1c18 in mice (Lan et al., 2024), cell cycle arrest, and apoptosis. A late luteal cell contributes to corpus luteum regression and eventual clearance from the ovary through luteolysis. [PMID:31803139, PMID:36205477, doi:/10.1101/2024.12.15.628550]

Logical Axioms:

```
SubClassOf(CL:4052047, CL:0000175)                          # is_a luteal cell
SubClassOf(CL:4052047, capable_of some GO:0006709)          # capable_of progesterone catabolic process
```

Synonyms:

- regressing corpus luteum cell (exact) [PMID:36205477]

1.5 Steroidogenic Stromal Cell

1.5.1 steroidogenic stromal cell of ovary (CL:4052045) — NEW

Definition: A stromal cell that is part of the ovarian stroma, characterized by its ability to synthesize steroid hormones. [PMID:36205477, doi:/10.1101/2024.12.15.628550]

Logical Axioms (Equivalence — necessary and sufficient conditions):

```
EquivalentClasses(CL:4052045,
    stromal cell (CL:0000499)
    AND part_of some ovarian stroma (UBERON:0006960)
    AND capable_of some steroid biosynthetic process (GO:0120178))
```

Synonyms:

- steroidogenic interstitial stromal cell (broad) [PMID:36205477]
- steroidogenic stromal cell (broad) [PMID:36205477]
- Enpp2- interstitial cell (related) [PMID:36205477]

1.6 Granulosa Cell (CL:0000501) — MODIFIED (hierarchy restructured)

The existing `granulosa cell` (CL:0000501) was updated to reflect the new developmental hierarchy:

Logical Axioms:

```
SubClassOf(CL:0000501, CL:0000174)                          # is_a steroid hormone secreting cell
SubClassOf(CL:0000501, CL:0002174)                          # is_a follicular cell of ovary
SubClassOf(CL:0000501, develops_from some CL:4033066)       # develops_from pre-granulosa cell
```

New subclasses:

- squamous granulosa cell (CL:4033083)
- cuboidal granulosa cell (CL:4033084)
- mural granulosa cell (CL:4033067)
- cumulus granulosa cell (CL:0000711) — renamed from "cumulus cell"

2. Fallopian Tube

2.1 fallopian tube epithelial cell (CL:4052018) — NEW

Definition: Any epithelial cell that is part of the fallopian tube. [Wikipedia:Fallopian_tube]

Logical Axioms (Equivalence — necessary and sufficient conditions):

```
EquivalentClasses(CL:4052018,
    epithelial cell (CL:0000066)
    AND part_of some fallopian tube (UBERON:0003889))
```

Synonyms:

- uterine tube epithelial cell (exact) [Wikipedia:Fallopian_tube]
- oviduct epithelial cell (broad) [Wikipedia:Fallopian_tube]

Role in hierarchy: This term serves as a grouping class. All fallopian tube epithelial cells (ciliated and non-ciliated subtypes) are automatically classified under this term by the OWL reasoner through the equivalence axiom.

2.2 fallopian tube multiciliated epithelial cell (CL:4030007) — RENAMED

Previous label: fallopian tube multiciliated cell

Definition: A multi-ciliated epithelial cell that is part of the fallopian tube, mainly found on the apex of the mucosal folds. This cell exhibits a columnar shape with an oval nucleus and is characterized by the presence of cilia on its surface. The coordinated beating of these cilia, together with peristaltic contractions, contributes to the self-propulsion of spermatozoa, the transport of ovum during ovulation and the transport of the fertilized ovum to the intramural fallopian tube. [PMID:16565155]

Logical Axioms:

```
SubClassOf(CL:4030007, CL:0005012)                          # is_a multiciliated epithelial cell
SubClassOf(CL:4030007, part_of some UBERON:0003889)          # part_of fallopian tube
SubClassOf(CL:4030007, part_of some UBERON:0007589)          # part_of ciliated epithelium (of fallopian tube)
```

Synonyms:

- fallopian tube ciliated cell (exact)
- fallopian tube multiciliated cell (exact) — previous label
- uterine tube ciliated epithelial cell (exact)
- oviduct ciliated cell (broad)

2.3 fallopian tube non-ciliated epithelial cell (CL:4052019) — NEW, RENAMED

Previous label (at creation): fallopian tube non-ciliated cell

Definition: Any epithelial cell that is part of the fallopian tube and lacks cilia.

Logical Axioms:

```
SubClassOf(CL:4052019, CL:0000066)                          # is_a epithelial cell
SubClassOf(CL:4052019, part_of some UBERON:0003889)          # part_of fallopian tube
SubClassOf(CL:4052019, lacks_part some GO:0005929)           # lacks_part cilium
```

Children (pre-existing, reclassified under this grouping term):

- fallopian tube secretory epithelial cell (CL:4030006)
- peg cell (CL:4033014)

Synonyms:

- fallopian tube non-ciliated cell (exact) — previous label

Distinguishing criteria from fallopian tube multiciliated epithelial cell (CL:4030007): Formally distinguished by the `lacks_part some cilium` axiom versus being a subclass of multiciliated epithelial cell. This provides a computable criterion for classification.

2.4 fallopian tube smooth muscle cell (CL:4052022) — NEW

Definition: A smooth muscle cell that is part of the fallopian tube. This cell is responsible for peristaltic contractions that facilitate gamete and embryo transport, fluid mixing, and embryo admission to the uterus. [PMID:31183831, PMID:31613440]

Logical Axioms (Equivalence — necessary and sufficient conditions):

```
EquivalentClasses(CL:4052022,
    smooth muscle cell (CL:0000192)
    AND part_of some fallopian tube (UBERON:0003889))
```

Synonyms:

- uterine tube smooth muscle cell (exact) [PMID:31613440]
- oviduct smooth muscle cell (broad) [PMID:31613440]

2.5 dual-feature fallopian tube progenitor cell (CL:4052070) — NEW

Definition: A bipotent progenitor cell within the human fallopian tube epithelium, characterized by the concurrent expression of epithelial (e.g., EpCAM/CD326) and endothelial (e.g., PECAM1/CD31) markers at both the cell surface and transcript levels. This cell has the capacity to differentiate into ciliated and secretory epithelial cells, as well as potentially endothelial/stromal lineages. Positioned at the apex of lineage bifurcation, the cell exhibits stem-like and endothelial features, representing an intermediate developmental state between undifferentiated progenitors and lineage-committed epithelial cells. [PMID:40475517]

Logical Axioms:

```
SubClassOf(CL:4052070, CL:0011026)                          # is_a progenitor cell
SubClassOf(CL:4052070, part_of some UBERON:8600124)          # part_of fallopian tube epithelium
SubClassOf(CL:4052070, in_taxon some NCBITaxon:9606)         # in_taxon Homo sapiens
```

Synonyms:

- unclassified fallopian tube progenitor (exact) [PMID:40475517]
- non-ciliated secretory epithelial cell 2-1 (NCSE2-1 cells) (narrow) [PMID:35320732]
- non-ciliated secretory epithelial cell 2-2 (NCSE2-2 cells) (narrow) [PMID:35320732]

3. Uterus

3.1 Endometrial Epithelial Cell Hierarchy

The endometrial epithelial cell hierarchy was reorganized to distinguish luminal from glandular epithelial cells, and to further classify ciliated vs. unciliated subtypes.

3.1.1 luminal epithelial cell of endometrium (CL:4052050) — NEW

Definition: An epithelial cell that is part of an endometrium luminal epithelium. [PMID:36273006]

Logical Axioms (Equivalence — necessary and sufficient conditions):

```
EquivalentClasses(CL:4052050,
    epithelial cell (CL:0000066)
    AND part_of some endometrium luminal epithelium (UBERON:0011949))
```

Synonyms:

- endometrial luminal epithelial cell (exact) [PMID:31317179]

Role in hierarchy: Grouping term for all luminal endometrial epithelial cells. The luminal endometrial unciliated epithelial cell (CL:4052023) is classified under this term.

3.1.2 luminal endometrial unciliated epithelial cell (CL:4052023) — NEW

Definition: An epithelial cell that is part of the endometrial luminal epithelium, forming a continuous layer lining the uterine cavity. This cell undergoes cyclical changes during the menstrual cycle, proliferating under estrogen in the proliferative phase, and differentiating under progesterone in the secretory phase to prepare for potential implantation. During the window of implantation, this cell changes from a tall columnar shape to a shorter columnar or cuboidal form, loses polarity, and becomes receptive to blastocyst implantation. [PMID:32929266, PMID:36581776, PMID:38559249, PMID:39198675]

Logical Axioms (Equivalence — necessary and sufficient conditions):

```
EquivalentClasses(CL:4052023,
    epithelial cell (CL:0000066)
    AND part_of some endometrium luminal epithelium (UBERON:0011949)
    AND capable_of some secretion by cell (GO:0032940))
```

Distinguishing criteria from glandular endometrial unciliated epithelial cell (CL:0002656): Both are unciliated secretory epithelial cells of the endometrium. The luminal cell is located in the endometrial luminal epithelium (UBERON:0011949), while the glandular cell is located in the endometrial gland (UBERON:0002451).

3.1.3 glandular endometrial unciliated epithelial cell (CL:0002656) — RENAMED

Previous label: glandular cell of endometrium

Definition: A glandular epithelial cell of the endometrium. Following ovulation, these cells secrete a glycogen-rich substance known as histotroph or uterine milk, which nourishes the embryo if implantation occurs. [GOC:tfm, PMID:25023676]

Logical Axioms (Equivalence — necessary and sufficient conditions):

```
EquivalentClasses(CL:0002656,
    epithelial cell (CL:0000066)
    AND part_of some endometrial gland (UBERON:0002451)
    AND capable_of some secretion by cell (GO:0032940))
```

Synonyms:

- glandular cell of endometrium (broad) — previous label

3.1.4 glandular epithelial cell of endometrium (CL:0009084) — RENAMED

Previous label: epithelial cell of endometrial gland

Definition: An epithelial cell that is part of an endometrium glandular epithelium. [ORCID:0000-0003-3440-1876, ORCID:0000-0003-4183-8865]

Logical Axioms (Equivalence — necessary and sufficient conditions):

```
EquivalentClasses(CL:0009084,
    epithelial cell (CL:0000066)
    AND part_of some endometrial glandular epithelium (UBERON:0012276))
```

Synonyms:

- endometrial glandular epithelial cell (exact)
- epithelial cell of endometrial gland (exact) — previous label

Cross-references: EFO:0010710

3.2 Stromal Cell of Endometrium (CL:0002255) — MODIFIED

Updated definition: A stromal cell of the endometrium, characterized by its fibroblast-like morphology, regenerative capacity, and ability to undergo decidualization. It differentiates into a decidual stromal cell during pregnancy, essential for embryo implantation and maintenance. This cell is involved in tissue proliferation, remodeling, and breakdown, responding to hormonal changes, particularly estrogen and progesterone. [PMID:11331626, PMID:30013421, PMID:30309298, PMID:31907034, Wikipedia:Decidualization]

Logical Axioms (Equivalence — necessary and sufficient conditions):

```
EquivalentClasses(CL:0002255,
    stromal cell (CL:0000499)
    AND part_of some endometrium (UBERON:0002337))
SubClassOf(CL:0002255, capable_of some decidualization (GO:0046697))
SubClassOf(CL:0002255, has_direct_input some CL:2000002)    # decidual cell
```

Synonyms updated:

- stromal cell of uterus (broad) — added
- endometrial stromal fibroblast (exact) [PMID:30309298] — added

3.3 Uterine Natural Killer Cell Hierarchy

A new hierarchy of uterine natural killer (uNK) cells was created, with one parent term and three human-specific subtypes distinguished by cell surface marker expression:

```
uterine natural killer cell → { uNK1 (human), uNK2 (human), uNK3 (human) }
```

Supplementary Text Table S1: The subtypes are defined using marker expression patterns with the `has_plasma_membrane_part` and `lacks_plasma_membrane_part` properties referencing Protein Ontology (PR) terms:

Marker	PR ID	uNK1	uNK2	uNK3
CD49a (ITGA1)	PR:P56199	+ (has)	+ (has)	+ (has)
NKp44 (NCR2)	PR:O95971	- (lacks)	- (lacks)	+ (has)
ITGB2 (CD18)	PR:P38570	- (lacks)	- (lacks)	+ (has)
CD39 (ENTPD1)	PR:P49961	+ (has)	- (lacks)	- (lacks)

Note: "+" in "has" column = has_plasma_membrane_part some PR:X; "-" in "lacks" column = lacks_plasma_membrane_part some PR:X

3.3.1 uterine natural killer cell (CL:4052028) — NEW

Definition: A natural killer cell that is part of the uterus, specifically within the endometrium during the non-pregnant state and in the decidua during pregnancy. This cell exhibits dynamic changes in frequency throughout the menstrual cycle, with lower levels during menstruation and a significant increase during the mid-secretory phase and early pregnancy. [PMID:35720413, PMID:39198675, Wikipedia:Uterine_natural_killer_cells]

Logical Axioms (Equivalence — necessary and sufficient conditions):

```
EquivalentClasses(CL:4052028,
    natural killer cell (CL:0000623)
    AND part_of some uterus (UBERON:0000995))
```

Children:

- decidual natural killer cell, human (CL:0002343) — pre-existing, reclassified as SubClassOf
- uterine natural killer cell 1, human (CL:4052051)
- uterine natural killer cell 2, human (CL:4052052)
- uterine natural killer cell 3, human (CL:4052053)

Synonyms:

- endometrial natural killer cell (narrow) [PMID:35720413]
- uNK cell (related, abbreviation) [PMID:39198675]

3.3.2 uterine natural killer cell 1, human (CL:4052051) — NEW

Definition: A uterine natural killer subset that is present in the endometrial lining during the non-pregnant state and in the decidua during pregnancy. It expresses the uterine resident marker CD49a and is distinguished from uNK2 and uNK3 by CD39 expression and the absence of CD103 and CD160. It also expresses higher levels of killer-cell immunoglobulin-like receptors (KIRs) and leukocyte immunoglobulin-like receptor B1 (LILRB1). Enriched in the endometrium post-ovulation and prominent in early pregnancy, uNK1 regulates trophoblast invasion and spiral artery remodeling. [PMID:30429548, PMID:34512661, PMID:34857954, PMID:35784314, PMID:39198675]

Logical Axioms (Equivalence — necessary and sufficient conditions):

```
EquivalentClasses(CL:4052051,
    uterine natural killer cell (CL:4052028)
    AND lacks_plasma_membrane_part some NKp44 (PR:O95971)
    AND lacks_plasma_membrane_part some ITGB2 (PR:P38570)
    AND has_plasma_membrane_part some CD39/ENTPD1 (PR:P49961)
    AND has_plasma_membrane_part some CD49a/ITGA1 (PR:P56199)
    AND in_taxon some Homo sapiens (NCBITaxon:9606))
```

Synonyms:

- uNK1 (related, abbreviation) [PMID:39198675]
- decidual natural killer cell 1 (narrow) [PMID:30429548]

3.3.3 uterine natural killer cell 2, human (CL:4052052) — NEW

Definition: A uterine natural killer subset that is present in the endometrial lining during the non-pregnant state and in the decidua during pregnancy, peaking in the first trimester. It expresses the uterine resident marker CD49a and is distinguished from uNK1 and uNK3 by the

absence of CD39, CD103, and CD160, with ITGB2 serving as a defining marker. Functionally, it produces more cytokines upon stimulation than uNK1, suggesting a role in immune defense, and secretes XCL1 chemokines, facilitating interactions with maternal dendritic cells and fetal extravillous trophoblasts. [PMID:30429548, PMID:34857954, PMID:35784314, PMID:39198675]

Logical Axioms (Equivalence — necessary and sufficient conditions):

```
EquivalentClasses(CL:4052052,
    uterine natural killer cell (CL:4052028)
    AND lacks_plasma_membrane_part some NKp44 (PR:O95971)
    AND lacks_plasma_membrane_part some ITGB2 (PR:P38570)
    AND lacks_plasma_membrane_part some CD39/ENTPD1 (PR:P49961)
    AND has_plasma_membrane_part some CD49a/ITGA1 (PR:P56199)
    AND in_taxon some Homo sapiens (NCBITaxon:9606))
```

Synonyms:

- uNK2 (related, abbreviation) [PMID:39198675]
- decidual natural killer cell 2 (narrow) [PMID:30429548]

3.3.4 uterine natural killer cell 3, human (CL:4052053) — NEW

Definition: A uterine natural killer subset found in the endometrial lining during the non-pregnant state and in the decidua during pregnancy, becoming the dominant uNK subset late in pregnancy. It expresses the uterine resident marker CD49a and is distinguished from uNK1 and uNK2 by CD160 and CD103 expression, the absence of CD39, and low KIR levels. Resembling intraepithelial ILC1 cells, uNK3 primarily supports uterine immune defense and indirectly influences trophoblast behavior through chemokine secretion, such as CCL5. [PMID:30429548, PMID:31959757, PMID:34857954, PMID:35784314, PMID:39198675]

Logical Axioms (Equivalence — necessary and sufficient conditions):

```
EquivalentClasses(CL:4052053,
    uterine natural killer cell (CL:4052028)
    AND lacks_plasma_membrane_part some CD39/ENTPD1 (PR:P49961)
    AND has_plasma_membrane_part some NKp44 (PR:O95971)
    AND has_plasma_membrane_part some ITGB2 (PR:P38570)
    AND has_plasma_membrane_part some CD49a/ITGA1 (PR:P56199)
    AND in_taxon some Homo sapiens (NCBITaxon:9606))
```

Synonyms:

- uNK3 (related, abbreviation) [PMID:39198675]
- decidual natural killer cell 3 (narrow) [PMID:30429548]

Distinguishing criteria among uNK subtypes: The three subtypes are formally distinguished by a combinatorial pattern of cell-surface markers encoded as OWL equivalence axioms. Each subtype is defined as a necessary and sufficient combination of the parent class (uterine natural killer cell), taxon constraint (Homo sapiens), and a specific pattern of `has_plasma_membrane_part` / `lacks_plasma_membrane_part` expressions for NKp44, ITGB2, CD39, and CD49a. This enables automated classification by OWL reasoners.

Summary Tables

Supplementary Text Table S2. New Cell Ontology Terms for the Female Reproductive System

CL ID	Term Label	Organ	Type	Equivalence Axiom	GitHub Issue
CL:4033066	pre-granulosa cell	Ovary	New	-	#2360
CL:4033067	mural granulosa cell	Ovary	New	-	#2360
CL:4033083	squamous granulosa cell	Ovary	New	-	#2602
CL:4033084	cuboidal granulosa cell	Ovary	New	-	#2602
CL:4033089	atretic follicular cell of ovary	Ovary	New	Yes	#2728
CL:4033090	atretic granulosa cell	Ovary	New	-	#2728
CL:4052010	pre-theca cell	Ovary	New	-	#2527
CL:4052012	interna theca cell	Ovary	New	Yes	#2528
CL:4052013	externa theca cell	Ovary	New	Yes	#2528
CL:4052027	atretic theca cell	Ovary	New	-	#2733
CL:4052045	steroidogenic stromal cell of ovary	Ovary	New	Yes	#2986
CL:4052046	early luteal cell	Ovary	New	-	#2980
CL:4052047	late luteal cell	Ovary	New	-	#2980
CL:4052018	fallopian tube epithelial cell	Fallopian tube	New	Yes	#2623
CL:4052019	fallopian tube non-ciliated epithelial cell	Fallopian tube	New	-	#2623
CL:4052022	fallopian tube smooth muscle cell	Fallopian tube	New	Yes	#2682
CL:4052070	dual-feature fallopian tube progenitor cell	Fallopian tube	New	-	#3196
CL:4052023	luminal endometrial unciliated epithelial cell	Uterus	New	Yes	#2687
CL:4052028	uterine natural killer cell	Uterus	New	Yes	#2688
CL:4052045	steroidogenic stromal cell of ovary	Ovary	New	Yes	#2986
CL:4052050	luminal epithelial cell of endometrium	Uterus	New	Yes	#2986

CL ID	Term Label	Organ	Type	Equivalence Axiom	GitHub Issue
CL:4052051	uterine natural killer cell 1, human	Uterus	New	Yes	#2984
CL:4052052	uterine natural killer cell 2, human	Uterus	New	Yes	#2991
CL:4052053	uterine natural killer cell 3, human	Uterus	New	Yes	#2992

Supplementary Text Table S3. Modified (Renamed/Restructured) Existing Terms

CL ID	New Label	Previous Label	Change Type	GitHub Issue
CL:0000711	cumulus granulosa cell	cumulus cell	Renamed	#2986
CL:0000501	granulosa cell	granulosa cell	Hierarchy restructured	#2602
CL:0000503	theca cell	theca cell	Definition updated, hierarchy expanded	#2527, #2528
CL:0002255	stromal cell of endometrium	stromal cell of endometrium	Definition enriched, axioms added	#2784
CL:0002656	glandular endometrial unciliated epithelial cell	glandular cell of endometrium	Renamed	#2695
CL:0009084	glandular epithelial cell of endometrium	epithelial cell of endometrial gland	Renamed	#2695, #2986
CL:4030007	fallopian tube multiciliated epithelial cell	fallopian tube multiciliated cell	Renamed	#2986
CL:4052019	fallopian tube non-ciliated epithelial cell	fallopian tube non-ciliated cell	Renamed	#2986

Supplementary Text Table S4. External Ontology Terms Referenced in Axioms

Ontology	ID	Label
UBERON	UBERON:0000155	theca of ovarian follicle
UBERON	UBERON:0000156	theca externa
UBERON	UBERON:0000157	theca interna
UBERON	UBERON:0000992	ovary
UBERON	UBERON:0000995	uterus
UBERON	UBERON:0001305	ovarian follicle
UBERON	UBERON:0002337	endometrium

Ontology	ID	Label
UBERON	UBERON:0002451	endometrial gland
UBERON	UBERON:0003889	fallopian tube
UBERON	UBERON:0003981	primordial ovarian follicle
UBERON	UBERON:0006960	ovarian stroma
UBERON	UBERON:0007589	ciliated epithelium
UBERON	UBERON:0011949	endometrium luminal epithelium
UBERON	UBERON:0012186	ovarian cortical stroma
UBERON	UBERON:0012276	endometrial glandular epithelium
UBERON	UBERON:8600124	fallopian tube epithelium
GO	GO:0001552	ovarian follicle atresia
GO	GO:0005929	cilium
GO	GO:0006701	progesterone biosynthetic process
GO	GO:0006709	progesterone catabolic process
GO	GO:0032940	secretion by cell
GO	GO:0046697	decidualization
GO	GO:0120178	steroid biosynthetic process
PATO	PATO:0001872	cuboidal
PATO	PATO:0002254	squamous
PR	PR:O95971	NKp44 (NCR2)
PR	PR:P38570	ITGB2 (CD18)
PR	PR:P49961	CD39 (ENTPD1)
PR	PR:P56199	CD49a (ITGA1)
NCBITaxon	NCBITaxon:9606	Homo sapiens

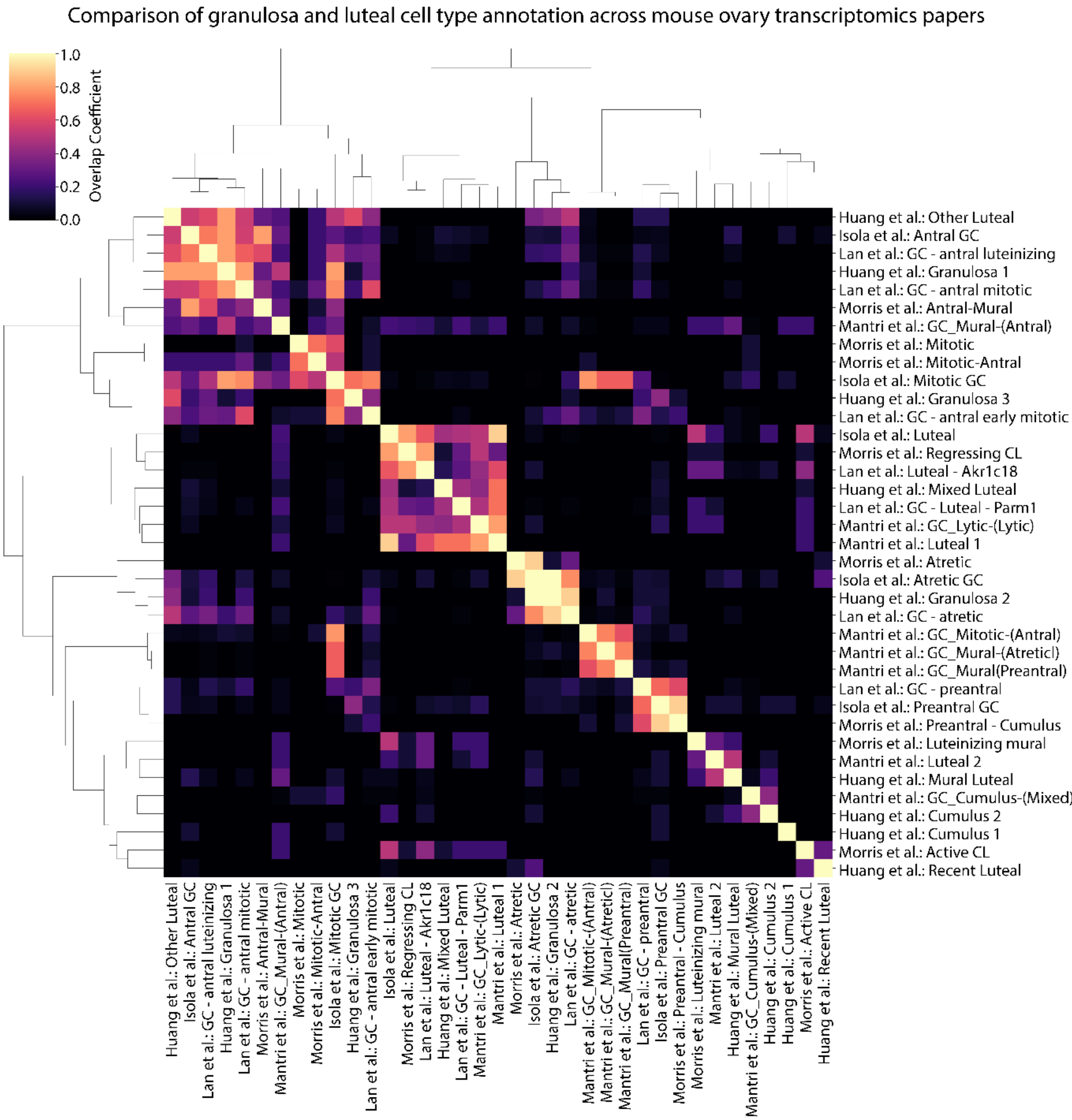

Fig. S1.

Comparison of cell type annotations across mouse ovary transcriptomics papers: Clustered heatmap of cell types using published marker genes to identify overlapping cell types across Morris *et al* (*16*), Mantri *et al* (*15*), Isola *et al* (*175*), Huang *et al* (*9*), and Lan *et al* (*8*).

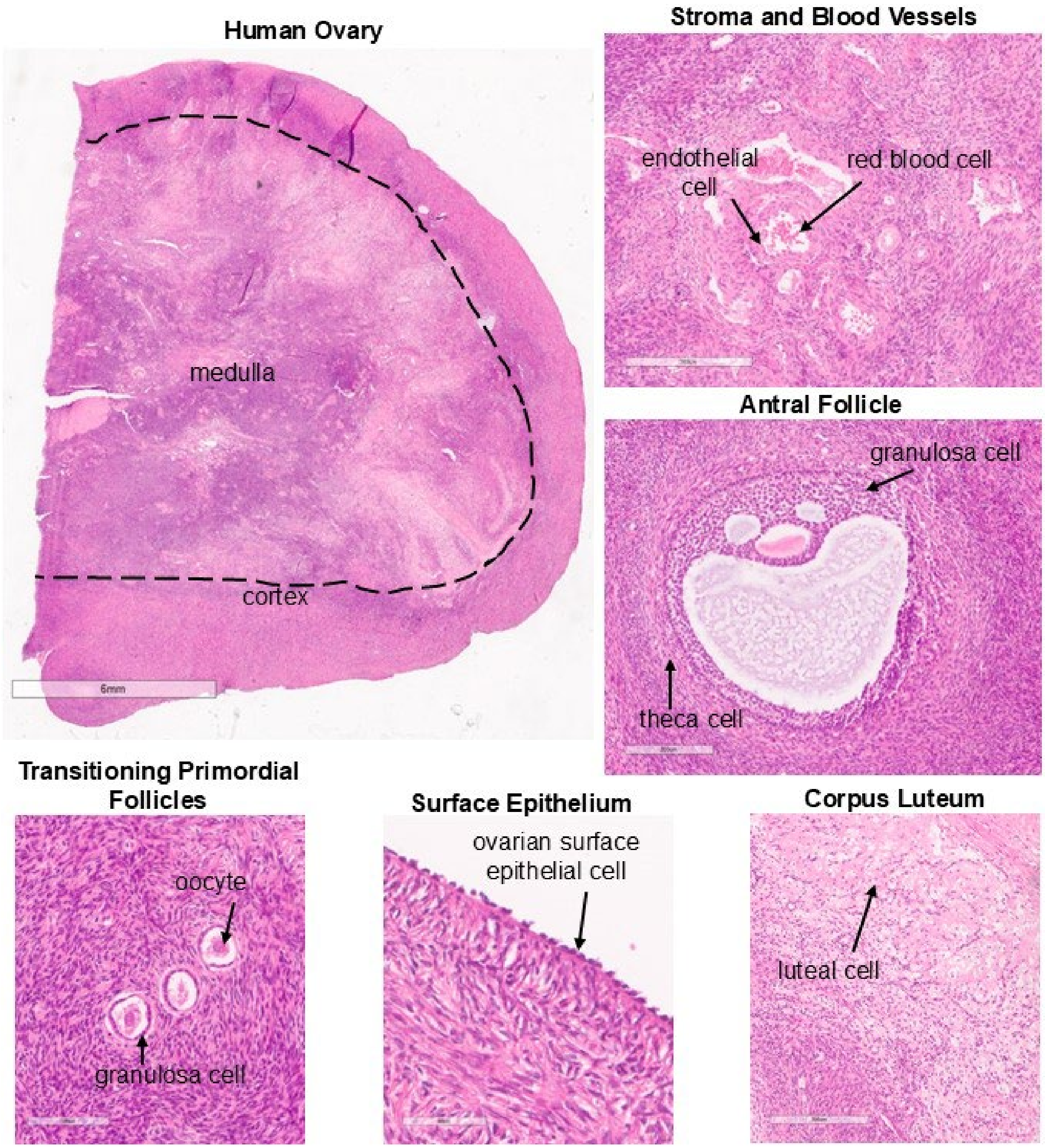

Fig. S2.

Annotated H&E Images of human ovary: H&E of healthy human ovarian tissue obtained from a 31-year-old donor.

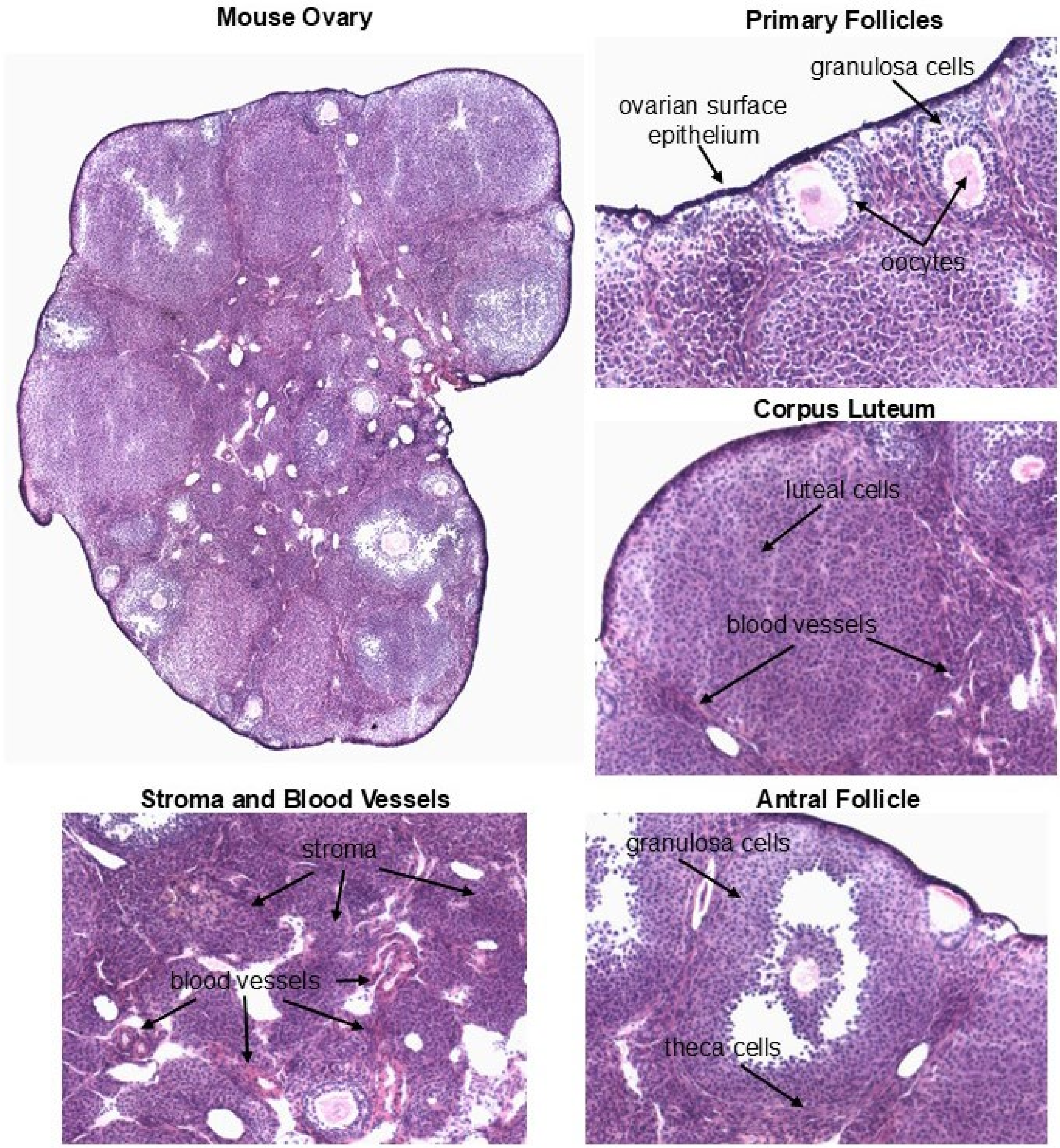

Fig. S3.

Annotated H&E Images of mouse ovary: H&E of healthy mouse ovarian tissue obtained from a 6-week-old C57BL/6J mouse.

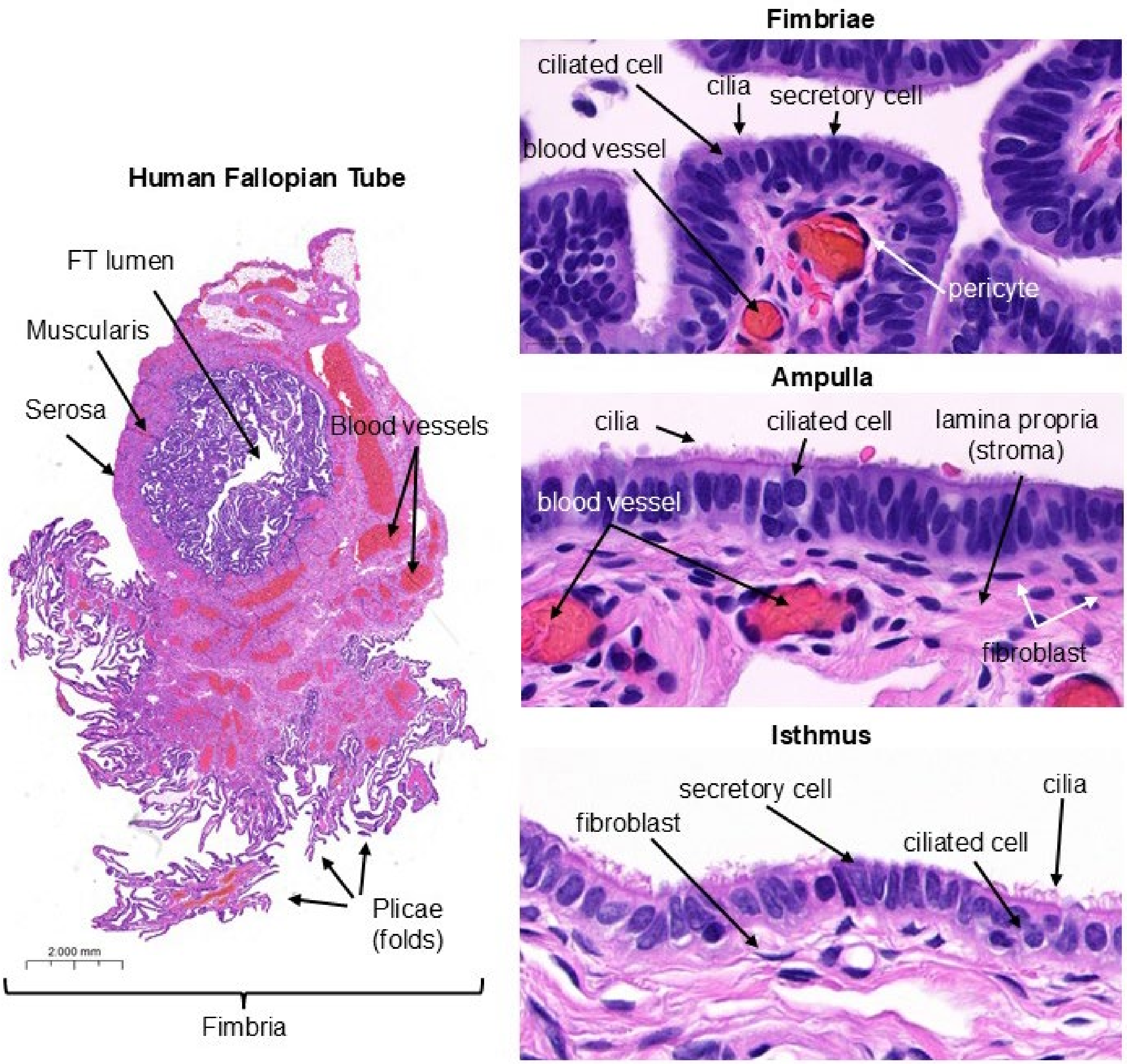

Fig. S4.

Annotated H&E Images of human fallopian tube: H&E of healthy human fallopian tube tissue obtained from a donor.

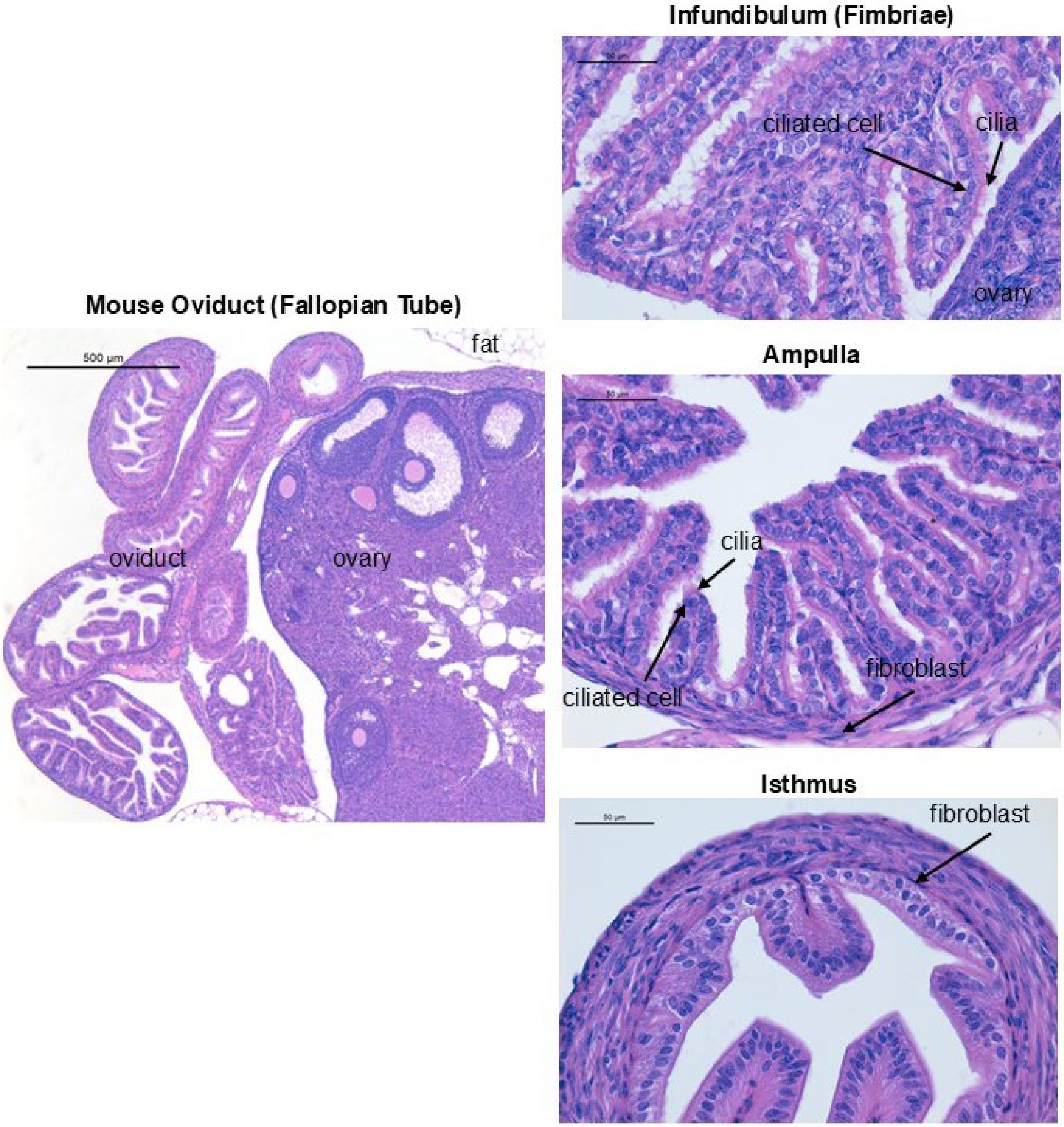

Fig. S5.

Annotated H&E Images of mouse oviduct: H&E of healthy mouse oviduct tissue obtained from a 4-month-old C57BL/6NHsd mouse.

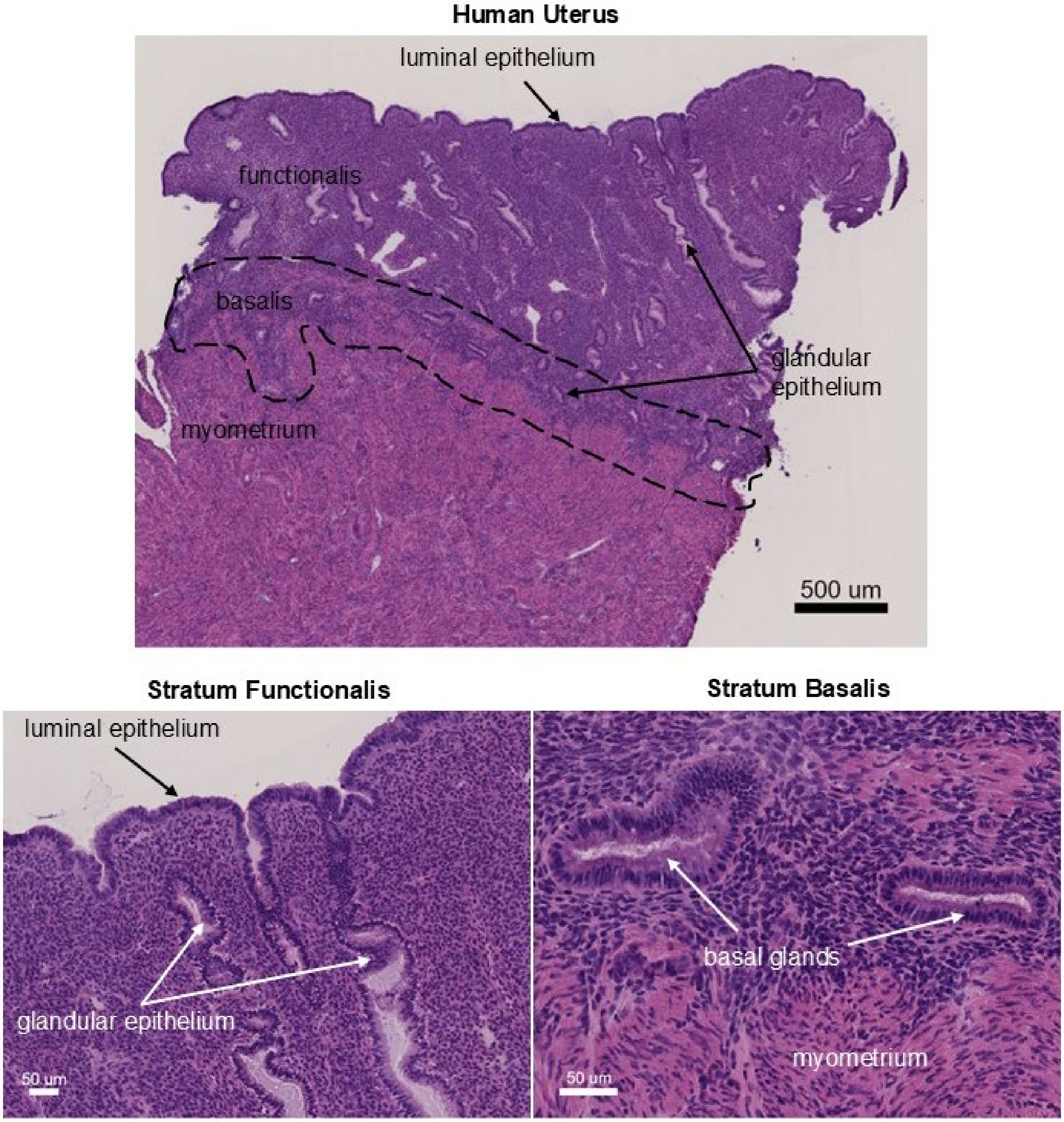

Fig. S6.

Annotated H&E Images of human uterus: Unpublished H&E of healthy human uterus tissue obtained from a donor in secretory phase.

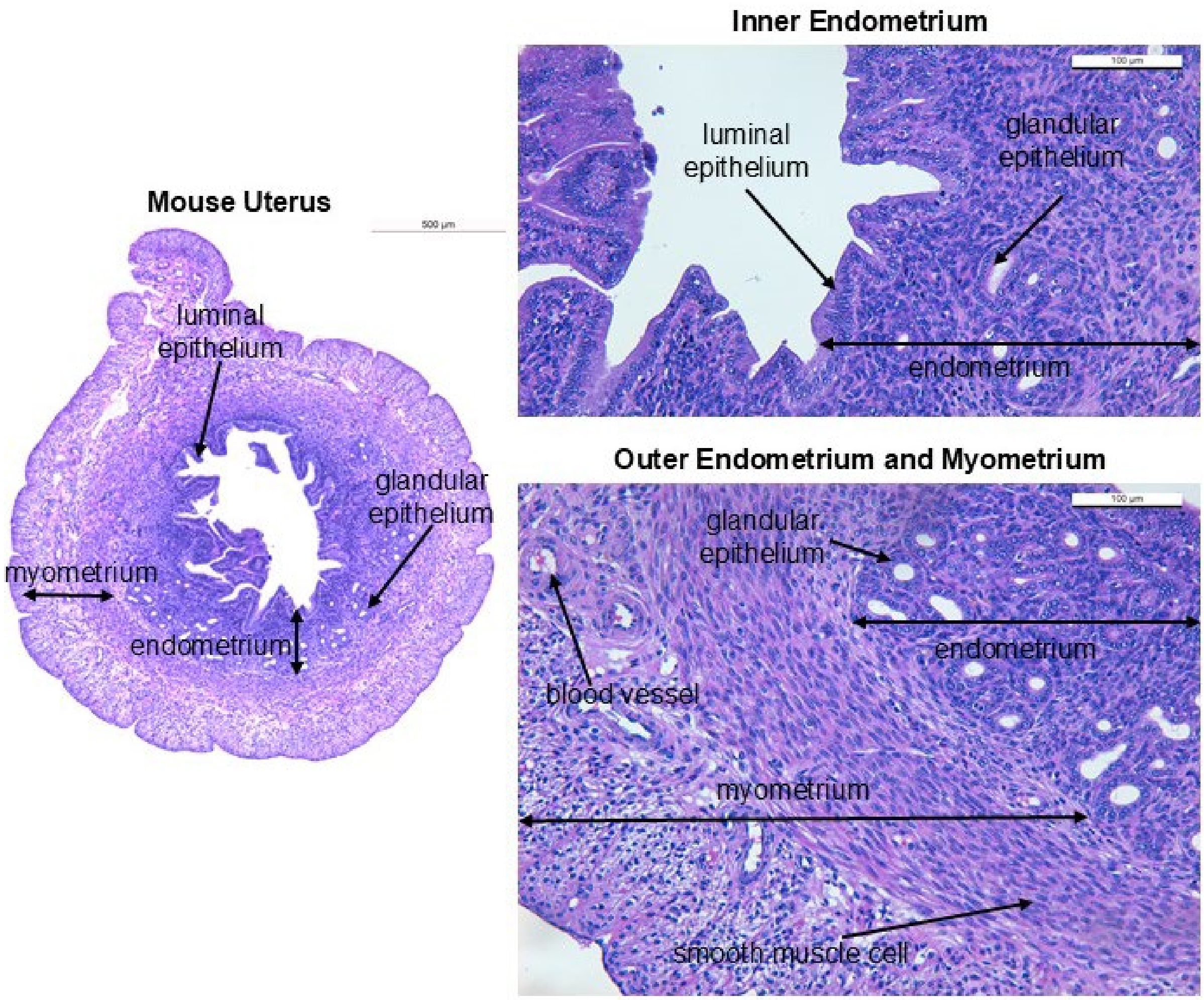

Fig. S7.

Annotated H&E Images of mouse uterine horn: H&E of healthy mouse uterine tissue obtained from a 4-month-old CD1 mouse in diestrus.

Table S1.

CL terms for female reproductive tract: This file contains cell type markers compiled by CZI Reproductive Tissue Atlas Annotation Jamboree attendees, where markers were mapped to CL terms for standardization. To include markers identified for cell phenotypes, some entries combined a CL term with a term from another ontology to describe the intended cell population (e.g., 'granulosa cell' with the modifier 'ovarian follicle atresia' to represent atretic granulosa cells). This is not an exhaustive list of markers and instead represents a snapshot of currently published data as a starting point for transcriptome dataset.

Refer to <*TableS1_Cell type markers_Final.xlsx*>

Table S2.
Human Reference Atlas (*174*) resources for human female reproductive tract.

**Organ**	**Description**	**Link to organ-specific data**
Ovary	The graph representation of the Anatomical Structures, Cell Types, plus Biomarkers (ASCT+B) table for Ovary dataset	Ovary
Uterus	The graph representation of the Anatomical Structures, Cell Types, plus Biomarkers (ASCT+B) table for Uterus dataset	Uterus
Fallopian tube	The graph representation of the Anatomical Structures, Cell Types, plus Biomarkers (ASCT+B) table for Fallopian Tube dataset	Fallopian tube
All organs	A visualization tool for human organ experts to explore and compare Anatomical Structures, Cell Types, and Biomarkers (ASCT+B) Tables	All organs

REFERENCES

1. S. Z. K. Tan, A. Puig-Barbe, D. Goutte-Gattat, C. Eastwood, B. Aevermann, A. Avola, J. P. Balhoff, I. U. Bayindir, J. Belfiore, A. R. Caron, D. S. Fischer, N. George, B. M. Gyori, M. A. Haendel, C. T. Hoyt, H. Kir, T. Lubiana, N. Matentzoglu, J. A. Overton, B. Peng, B. Peters, E. M. Quardokus, P. L. Ray, P. Roncaglia, A. D. Rivera, R. Stefancsik, W. K. Teh, S. Toro, N. Vasilevsky, C. Xu, Y. Zhang, R. H. Scheuermann, C. J. Mungall, A. D. Diehl, D. Osumi-Sutherland, The Cell Ontology in the age of single-cell omics. *Sci. Data* **13**, 946 (2026).

2. S. Wang, I. V. Larina, In vivo dynamic 3D imaging of oocytes and embryos in the mouse oviduct. *Cell Rep.* **36**, 109382 (2021).

3. C. Trapnell, Defining cell types and states with single-cell genomics. *Genome Res.* **25**, 1491–1498 (2015).

4. A. Dance, What is a cell type, really? The quest to categorize life's myriad forms. *Nature* **633**, 754–756 (2024).

5. H. Zeng, What is a cell type and how to define it? *Cell* **185**, 2739–2755 (2022).

6. Y. Panina, P. Karagiannis, A. Kurtz, G. N. Stacey, W. Fujibuchi, Human Cell Atlas and cell-type authentication for regenerative medicine. *Exp. Mol. Med.* **52**, 1443–1451 (2020).

7. S. Liu, C. Trapnell, Single-cell transcriptome sequencing: Recent advances and remaining challenges. *F1000Res* **5**, 182 (2016).

8. T. C. T. Lan, A. Kochersberger, R. Raichur, S. Szady, H. Tran, R. Simeonova, A. Helmuth, A. Minagar, M. J. Orozco Fuentes, V. Kumar, G. Marrero, I. Barrera, S. Mangiameli, A. K. Shalek, P. C. Sabeti, F. Chen, D. S. Fischer, J. L. Garrison, H. Chung, Aging disrupts spatiotemporal coordination in the cycling murine ovary. *Nat Aging* **6**, 1244–1266 (2026).

9. R. Huang, C. E. Kratka, J. Pea, C. McCann, J. Nelson, J. P. Bryan, L. T. Zhou, D. D. Russo, E. J. Zaniker-Gomez, A. H. Gandhi, A. K. Shalek, B. Cleary, S. L. Farhi, F. E. Duncan, B. A. Goods,

Single-cell and spatiotemporal profile of ovulation in the mouse ovary. *PLOS Biol.* **23**, e3003193 (2025).

10. D. Osumi-Sutherland, C. Xu, M. Keays, A. P. Levine, P. V. Kharchenko, A. Regev, E. Lein, S. A. Teichmann, Cell type ontologies of the Human Cell Atlas. *Nat. Cell Biol.* **23**, 1129–1135 (2021).

11. R. Hoehndorf, P. N. Schofield, G. V. Gkoutos, The role of ontologies in biological and biomedical research: A functional perspective. *Brief. Bioinform.* **16**, 1069–1080 (2015).

12. CZI Cell Science Program, S. Abdulla, B. Aevermann, P. Assis, S. Badajoz, S. M. Bell, E. Bezzi, B. Cakir, J. Chaffer, S. Chambers, J. M. Cherry, T. Chi, J. Chien, L. Dorman, P. Garcia-Nieto, N. Gloria, M. Hastie, D. Hegeman, J. Hilton, T. Huang, A. Infeld, A.-M. Istrate, I. Jelic, K. Katsuya, Y. J. Kim, K. Liang, M. Lin, M. Lombardo, B. Marshall, B. Martin, F. McDade, C. Megill, N. Patel, A. Predeus, B. Raymor, B. Robatmili, D. Rogers, E. Rutherford, D. Sadgat, A. Shin, C. Small, T. Smith, P. Sridharan, A. Tarashansky, N. Tavares, H. Thomas, A. Tolopko, M. Urisko, J. Yan, G. Yeretssian, J. Zamanian, A. Mani, J. Cool, A. Carr, CZ CELLxGENE Discover: A single-cell data platform for scalable exploration, analysis and modeling of aggregated data. *Nucleic Acids Res.* **53**, D886–D900 (2025).

13. HuBMAP Consortium, The human body at cellular resolution: The NIH Human Biomolecular Atlas Program. *Nature* **574**, 187–192 (2019).

14. A. Regev, S. A. Teichmann, E. S. Lander, I. Amit, C. Benoist, E. Birney, B. Bodenmiller, P. Campbell, P. Carninci, M. Clatworthy, H. Clevers, B. Deplancke, I. Dunham, J. Eberwine, R. Eils, W. Enard, A. Farmer, L. Fugger, B. Göttgens, N. Hacohen, M. Haniffa, M. Hemberg, S. Kim, P. Klenerman, A. Kriegstein, E. Lein, S. Linnarsson, E. Lundberg, J. Lundeberg, P. Majumder, J. C. Marioni, M. Merad, M. Mhlanga, M. Nawijn, M. Netea, G. Nolan, D. Pe’er, A. Phillipakis, C. P. Ponting, S. Quake, W. Reik, O. Rozenblatt-Rosen, J. Sanes, R. Satija, T. N. Schumacher, A. Shalek, E. Shapiro, P. Sharma, J. W. Shin, O. Stegle, M. Stratton, M. J. T. Stubbington, F. J. Theis, M. Uhlen, A. Van Oudenaarden, A. Wagner, F. Watt, J. Weissman, B. Wold, R. Xavier, N. Yosef, Human Cell Atlas Meeting Participants, The Human Cell Atlas. *eLife* **6**, e27041 (2017).

15. M. Mantri, H. H. Zhang, E. Spanos, Y. A. Ren, I. De Vlaminck, A spatiotemporal molecular atlas of the ovulating mouse ovary. *Proc. Natl. Acad. Sci. U.S.A.* **121**, e2317418121 (2024).

16. M. E. Morris, M.-C. Meinsohn, M. Chauvin, H. D. Saatcioglu, A. Kashiwagi, N. A. Sicher, N. Nguyen, S. Yuan, R. Stavely, M. Hyun, P. K. Donahoe, B. L. Sabatini, D. Pépin, A single-cell atlas of the cycling murine ovary. *eLife* **11**, e77239 (2022).

17. E. A. Gaylord, M. H. Foecke, R. M. Samuel, B. Soygur, A. M. Detweiler, T. I. McIntyre, L. C. Dorman, M. Borja, A. E. Laird, R. Arjyal, J. Du, J. M. Gardner, N. Neff, F. Fattahi, D. J. Laird, Comparative analysis of human and mouse ovaries across age. *Science* **390**, eadx0659 (2025).

18. B. Zhao, K. Dai, S. Li, G. Zhang, W. Lv, W. Liu, Y. Cai, F. Wang, H. Zhou, D. Guan, Y. Zhang, Integrative single-cell transcriptomics of sheep ovarian development reveals dynamic transcriptional programs relevant to reproductive traits. *iScience* **28**, 112422 (2025).

19. I. Winkler, A. Tolkachov, F. Lammers, P. Lacour, K. Daugelaite, N. Schneider, M.-L. Koch, J. Panten, F. Grünschläger, T. Poth, B. M. D. Ávila, A. Schneider, S. Haas, D. T. Odom, Â. Gonçalves, The cycling and aging mouse female reproductive tract at single-cell resolution. *Cell* **187**, 981–998.e25 (2024).

20. J. Sipes, S. Rayamajhi, L. E. Bantis, R. Madan, A. Mitra, R. V. Puri, M. M. Rahman, F. Ahmmed, H. B. Pathak, A. K. Godwin, Spatial transcriptomic profiling of the human fallopian tube epithelium reveals region-specific gene expression patterns. *Commun. Biol.* **8**, 520 (2025).

21. S. Cheng, G. Altmeppen, C. So, L. M. Welp, S. Penir, T. Ruhwedel, K. Menelaou, K. Harasimov, A. Stützer, M. Blayney, K. Elder, W. Möbius, H. Urlaub, M. Schuh, Mammalian oocytes store mRNAs in a mitochondria-associated membraneless compartment. *Science* **378**, eabq4835 (2022).

22. S. E. Pors, D. Nikiforov, J. Cadenas, Z. Ghezelayagh, Y. Wakimoto, L. A. Z. Jara, J. Cheng, M. Dueholm, K. T. Macklon, E. M. Flachs, L. S. Mamsen, S. G. Kristensen, C. Y. Andersen, Oocyte diameter predicts the maturation rate of human immature oocytes collected ex vivo. *J. Assist. Reprod. Genet.* **39**, 2209–2214 (2022).

23. S. Wang, Y. Zheng, J. Li, Y. Yu, W. Zhang, M. Song, Z. Liu, Z. Min, H. Hu, Y. Jing, X. He, L. Sun, L. Ma, C. R. Esteban, P. Chan, J. Qiao, Q. Zhou, J. C. Izpisua Belmonte, J. Qu, F. Tang, G.-H. Liu, Single-cell transcriptomic atlas of primate ovarian aging. *Cell* **180**, 585–600.e19 (2020).

24. Y. Zhang, Z. Yan, Q. Qin, V. Nisenblat, H.-M. Chang, Y. Yu, T. Wang, C. Lu, M. Yang, S. Yang, Y. Yao, X. Zhu, X. Xia, Y. Dang, Y. Ren, P. Yuan, R. Li, P. Liu, H. Guo, J. Han, H. He, K. Zhang, Y. Wang, Y. Wu, M. Li, J. Qiao, J. Yan, L. Yan, Transcriptome landscape of human folliculogenesis reveals oocyte and granulosa cell interactions. *Mol. Cell* **72**, 1021–1034.e4 (2018).

25. K. Tomida, H. T. Ong, J. L. Young, C. J. Chan, Capturing ovarian dynamics through spatial profiling of the mechano-microenvironment. *Semin. Cell Dev. Biol.* **175**, 103642 (2025).

26. Y.-Y. Chen, D. D. Russo, R. S. Drake, F. E. Duncan, A. K. Shalek, B. A. Goods, T. K. Woodruff, Single-cell transcriptomics of staged oocytes and somatic cells reveal novel regulators of follicle activation. *Reproduction* **164**, 55–70 (2022).

27. E. H. Ernst, M. L. Grøndahl, S. Grund, K. Hardy, A. Heuck, L. Sunde, S. Franks, C. Y. Andersen, P. Villesen, K. Lykke-Hartmann, Dormancy and activation of human oocytes from primordial and primary follicles: Molecular clues to oocyte regulation, *Hum. Reprod.* **32**, 1684–1700. (2017)

28. H. Kubo, C. J. McCauley, M. B. Zelinski, M. M. Laronda, Establishing the baseline: Understanding follicle activation morphometrics in multiple mammalian species. *Biol. Reprod.* **112**, 1123–1133 (2025).

29. J. C. Yano Maher, M. B. Zelinski, K. H. Oktay, F. E. Duncan, J. H. Segars, M. E. Lujan, H. Lou, B. H. Yun, S. N. Hanfling, L. E. Schwartz, M. M. Laronda, L. M. Halvorson, K. E. O'Neill, V. Gomez-Lobo, Classification system of human ovarian follicle morphology: Recommendations of the National Institute of Child Health and Human Development—Sponsored ovarian nomenclature workshop. *Fertil. Steril.* **123**, 761–778 (2025).

30. H. J. Clarke, History, origin, and function of transzonal projections: The bridges of communication between the oocyte and its environment. *Anim. Reprod.* **15**, 215–223 (2018).

31. S. E. Wamaitha, X. Nie, E. C. Pandolfi, X. Wang, Y. Yang, J.-B. Stukenborg, B. R. Cairns, J. Guo, A. T. Clark, Single-cell analysis of the developing human ovary defines distinct insights into ovarian somatic and germline progenitors. *Dev. Cell* **58**, 2097–2111.e3 (2023).

32. W. Niu, A. C. Spradling, Two distinct pathways of pregranulosa cell differentiation support follicle formation in the mouse ovary. *Proc. Natl. Acad. Sci. U.S.A.* **117**, 20015–20026 (2020).

33. H. Kishi, Y. Kitahara, F. Imai, K. Nakao, H. Suwa, Expression of the gonadotropin receptors during follicular development. *Reprod. Med. Biol.* **17**, 11–19 (2018).

34. S. Assou, T. Anahory, V. Pantesco, T. Le Carrour, F. Pellestor, B. Klein, L. Reyftmann, H. Dechaud, J. De Vos, S. Hamamah, The human cumulus-oocyte complex gene-expression profile. *Hum. Reprod.* **21**, 1705–1719 (2006).

35. A. Govahi, S. Eghbali, N. E. Ghiasi, Z. Zandieh, M. Ajdary, R. Mehdizadeh, M. Mehdizadeh, Changes in the transcriptomic profile of cumulus cells under the influence of cumulus-oocytes complex pre-incubation. *Sci. Rep.* **14**, 17183 (2024).

36. D. A. Magoffin, Ovarian theca cell. *Int. J. Biochem. Cell Biol.* **37**, 1344–1349 (2005).

37. N. L. Guahmich, L. Man, J. Wang, L. Arazi, E. Kallinos, A. Topper-Kroog, G. Grullon, K. Zhang, J. Stewart, N. Schatz-Siemers, S. H. Jones, R. Bodine, N. Zaninovic, G. Schattman, Z. Rosenwaks, D. James, Human theca arises from ovarian stroma and is comprised of three discrete subtypes. *Commun. Biol.* **6**, 7 (2023).

38. J. Nio-Kobayashi, T. Iwanaga, Differential cellular localization of galectin-1 and galectin-3 in the regressing corpus luteum of mice and their possible contribution to luteal cell elimination. *J. Histochem. Cytochem.* **58**, 741–749 (2010).

39. C. Stocco, C. Telleria, G. Gibori, The molecular control of corpus luteum formation, function, and regression. *Endocr. Rev.* **28**, 117–149 (2007).

40. A. Bachelot, N. Binart, Reproductive role of prolactin. *Reproduction* **133**, 361–369 (2007).

41. G. W. Smith, R. Meidan, Ever-changing cell interactions during the life span of the corpus luteum: Relevance to luteal regression. *Reprod. Biol.* **14**, 75–82 (2014).

42. M. C. Wiltbank, S. M. Salih, M. O. Atli, W. Luo, C. L. Bormann, J. S. Ottobre, C. M. Vezina, V. Mehta, F. J. Diaz, S. J. Tsai, R. Sartori, Comparison of endocrine and cellular mechanisms regulating the corpus luteum of primates and ruminants. *Anim. Reprod.* **9**, 242–259 (2012).

43. E. J. Zaniker, J. Zhang, D. Russo, R. Huang, K. Suritis, R. S. Drake, E. Barlow-Smith, A. K. Shalek, T. K. Woodruff, S. Xiao, B. A. Goods, F. E. Duncan, Follicle-intrinsic and spatially distinct molecular programs drive follicle rupture and luteinization during ex vivo mammalian ovulation. *Commun. Biol.* **7**, 1374 (2024).

44. X. Fan, S. M. C. De Sousa Lopes, Molecular makeup of the human adult ovary. *Curr. Opin. Endocr. Metab. Res.* **18**, 187–193 (2021).

45. N. Chegini, N. Ramani, C. V. Rao, Morphological and biochemical characterization of small and large bovine luteal cells during pregnancy. *Mol. Cell. Endocrinol.* **37**, 89–102 (1984).

46. Z.-H. Zhao, T.-G. Meng, X.-Y. Chen, F. Gao, H. Schatten, X.-H. Ou, Q.-Y. Sun, Spatiotemporal and single-cell atlases to dissect regional specific cell types of the developing ovary. *Commun. Biol.* **8**, 849 (2025).

47. G. F. Erickson, D. A. Magoffin, C. A. Dyer, C. Hofeditz, The ovarian androgen producing cells: A review of structure/function relationships. *Endocr. Rev.* **6**, 371–399 (1985).

48. P. P. Szotek, H. L. Chang, K. Brennand, A. Fujino, R. Pieretti-Vanmarcke, C. Lo Celso, D. Dombkowski, F. Preffer, K. S. Cohen, J. Teixeira, P. K. Donahoe, Normal ovarian surface epithelial label-retaining cells exhibit stem/progenitor cell characteristics. *Proc. Natl. Acad. Sci. U.S.A.* **105**, 12469–12473 (2008).

49. J. Wang, C. Liu, L. He, Z. Xie, L. Bai, W. Yu, Z. Wang, Y. Lu, D. Gao, J. Fu, L. Zhang, Y. A. Zeng, Selective YAP activation in Procr cells is essential for ovarian stem/progenitor expansion and epithelium repair. *eLife* **11**, e75449 (2022).

50. E. L. Tsui, K. E. O'Neill, R. D. LeDuc, A. Shikanov, V. Gomez-Lobo, M. M. Laronda, Creating a common language for the subanatomy of the ovary. *Biol. Reprod.* **108**, 1–4 (2023).

51. E. L. Tsui, C. J. Harris, E. E. Rowell, M. M. Laronda, Human ovarian gross morphology and subanatomy across puberty: Insights from tissue donated during fertility preservation. *F S Rep.* **4**, 196–205 (2023).

52. Y. Yamamoto, A. Kuwahara, Y. Taniguchi, M. Yamasaki, Y. Tanaka, Y. Mukai, M. Yamashita, T. Matsuzaki, T. Yasui, M. Irahara, Tumor necrosis factor alpha inhibits ovulation and induces granulosa cell death in rat ovaries. *Reprod. Med. Biol.* **14**, 107–115 (2015).

53. W. J. Murdoch, A. C. McDonnel, Roles of the ovarian surface epithelium in ovulation and carcinogenesis. *Reproduction* **123**, 743–750 (2002).

54. N. Auersperg, A. S. T. Wong, K.-C. Choi, S. K. Kang, P. C. K. Leung, Ovarian surface epithelium: Biology, endocrinology, and pathology. *Endocr. Rev.* **22**, 255–288 (2001).

55. N. J. Bowen, L. D. Walker, L. V. Matyunina, S. Logani, K. A. Totten, B. B. Benigno, J. F. McDonald, Gene expression profiling supports the hypothesis that human ovarian surface epithelia are multipotent and capable of serving as ovarian cancer initiating cells. *BMC Med. Genomics* **2**, 71 (2009).

56. L. Garcia-Alonso, V. Lorenzi, C. I. Mazzeo, J. P. Alves-Lopes, K. Roberts, C. Sancho-Serra, J. Engelbert, M. Marečková, W. H. Gruhn, R. A. Botting, T. Li, B. Crespo, S. Van Dongen, V. Y. Kiselev, E. Prigmore, M. Herbert, A. Moffett, A. Chédotal, O. A. Bayraktar, A. Surani, M. Haniffa, R. Vento-Tormo, Single-cell roadmap of human gonadal development. *Nature* **607**, 540–547 (2022).

57. R. Perets, G. A. Wyant, K. W. Muto, J. G. Bijron, B. B. Poole, K. T. Chin, J. Y. H. Chen, A. W. Ohman, C. D. Stepule, S. Kwak, A. M. Karst, M. S. Hirsch, S. R. Setlur, C. P. Crum, D. M.

Dinulescu, R. Drapkin, Transformation of the fallopian tube secretory epithelium leads to high-grade serous ovarian cancer in Brca;Tp53;Pten models. *Cancer Cell* **24**, 751–765 (2013).

58. S. Zhang, I. Dolgalev, T. Zhang, H. Ran, D. A. Levine, B. G. Neel, Both fallopian tube and ovarian surface epithelium are cells-of-origin for high-grade serous ovarian carcinoma. *Nat. Commun.* **10**, 5367 (2019).

59. A. Flesken-Nikitin, C. Q. Ralston, D.-J. Fu, A. J. De Micheli, D. J. Phuong, B. A. Harlan, C. S. Ashe, A. P. Armstrong, D. W. McKellar, S. Ghuwalewala, L. H. Ellenson, J. C. Schimenti, B. D. Cosgrove, A. Yu. Nikitin, Pre-ciliated tubal epithelial cells are prone to initiation of high-grade serous ovarian carcinoma. *Nat. Commun.* **15**, 8641 (2024).

60. I. Beddows, S. Djirackor, D. K. Omran, E. Jung, N. N. Shih, R. Roy, A. Hechmer, A. Olshen, G. Adelmant, A. Tom, J. Morrison, M. Adams, D. C. Rohrer, L. E. Schwartz, C. L. Pearce, H. Auman, J. A. Marto, C. W. Drescher, R. Drapkin, H. Shen, Impact of BRCA mutations, age, surgical indication, and hormone status on the molecular phenotype of the human fallopian tube. *Nat. Commun.* **16**, 2981 (2025).

61. M. Weigert, Y. Li, L. Zhu, H. Eckart, P. Bajwa, R. Krishnan, S. Ackroyd, R. Lastra, A. Bilecz, A. Basu, E. Lengyel, M. Chen, A cell atlas of the human fallopian tube throughout the menstrual cycle and menopause. *Nat. Commun.* **16**, 372 (2025).

62. C. A. Ishak, S. A. Marhon, N. Tchrakian, A. Hodgson, H. Loo Yau, I. M. Gonzaga, M. Peralta, I. M. Lungu, S. Gomez, S.-B. Liang, S. Y. Shen, R. Chen, J. Chen, B. Chatterjee, K. N. Wanniarachchi, J. Lee, N. Zehrbach, A. Hosseini, P. Mehdipour, S. Sun, A. Solovyov, I. Ettayebi, K. E. Francis, A. He, T. Wu, S. Feng, T. Da Silva Medina, F. Campos De Almeida, J. Bayani, J. Li, S. MacDonald, Y. Wang, S. S. Garcia, E. Arthofer, N. Diab, A. Srivastava, P. T. Austin, P. J. B. Sabatini, B. D. Greenbaum, C. A. O’Brien, T. G. Shepherd, M. S. Tsao, K. B. Chiappinelli, A. M. Oza, B. A. Clarke, R. Rottapel, S. Lheureux, D. D. De Carvalho, Chronic viral mimicry induction following p53 loss promotes immune evasion. *Cancer Discov.* **15**, 793–817 (2025).

63. D. Y. Paik, D. M. Janzen, A. M. Schafenacker, V. S. Velasco, M. S. Shung, D. Cheng, J. Huang, O. N. Witte, S. Memarzadeh, Stem-like epithelial cells are concentrated in the distal end of the Fallopian tube: A site for injury and serous cancer initiation. *Stem Cells* **30**, 2487–2497 (2012).

64. A. Ng, S. Tan, G. Singh, P. Rizk, Y. Swathi, T. Z. Tan, R. Y.-J. Huang, M. Leushacke, N. Barker, Lgr5 marks stem/progenitor cells in ovary and tubal epithelia. *Nat. Cell Biol.* **16**, 745–757 (2014).

65. R. Lefebvre, P. J. Chenoweth, M. Drost, C. T. LeClear, M. MacCubbin, J. T. Dutton, S. S. Suarez, Characterization of the oviductal sperm reservoir in cattle. *Biol. Reprod.* **53**, 1066–1074 (1995).

66. S. S. Suarez, The oviductal sperm reservoir in mammals: Mechanisms of formation. *Biol. Reprod.* **58**, 1105–1107 (1998).

67. B. E. Barton, G. G. Herrera, P. Anamthathmakula, J. K. Rock, A. M. Willie, E. A. Harris, K.-I. Takemaru, W. Winuthayanon, Roles of steroid hormones in oviductal function. *Reproduction* **159**, R125-R137 (2020).

68. T. Ito, Scanning electron microscopic observations on the surface of the human fallopian tube and uterine endometrium. *Keio J. Med.* **24**, 159–174 (1975).

69. F. N. Critoph, K. J. Dennis, The cellular composition of the human oviduct epithelium. *BJOG* **84**, 219–221 (1977).

70. M. T. Comer, H. J. Leese, J. Southgate, Induction of a differentiated ciliated cell phenotype in primary cultures of fallopian tube epithelium. *Hum. Reprod.* **13**, 3114–3120 (1998).

71. J. Donnez, F. Casanas-Roux, J. Caprasse, J. Ferin, K. Thomas, Cyclic changes in ciliation, cell height, and mitotic activity in human tubal epithelium during reproductive life. *Fertil. Steril.* **43**, 554–559 (1985).

72. E. Lengyel, Y. Li, M. Weigert, L. Zhu, H. Eckart, M. Javellana, S. Ackroyd, J. Xiao, S. Olalekan, D. Glass, S. Iyer, R. Krishnan, A. J. Bilecz, R. Lastra, M. Chen, A. Basu, A molecular atlas of the human postmenopausal fallopian tube and ovary from single-cell RNA and ATAC sequencing. *Cell Rep.* **41**, 111838 (2022).

73. N. D. Ulrich, Y.-C. Shen, Q. Ma, K. Yang, D. F. Hannum, A. Jones, J. Machlin, J. F. Randolph Jr., Y. R. Smith, S. B. Schon, A. Shikanov, E. E. Marsh, R. Lieberman, S. J. Gurczynski, B. B. Moore, J. Z. Li, S. Hammoud, Cellular heterogeneity of human fallopian tubes in normal and hydrosalpinx disease states identified using scRNA-seq. *Dev. Cell* **57**, 914–929.e7 (2022).

74. A. Ghosh, S. M. Syed, P. S. Tanwar, In vivo genetic cell lineage tracing reveals that oviductal secretory cells self-renew and give rise to ciliated cells. *Development* **144**, 3031–3041 (2017).

75. H. Q. Dinh, X. Lin, F. Abbasi, R. Nameki, M. Haro, C. E. Olingy, H. Chang, L. Hernandez, S. A. Gayther, K. N. Wright, P.-J. Aspuria, B. Y. Karlan, R. I. Corona, A. Li, B. J. Rimel, M. T. Siedhoff, F. Medeiros, K. Lawrenson, Single-cell transcriptomics identifies gene expression networks driving differentiation and tumorigenesis in the human fallopian tube. *Cell Rep.* **35**, 108978 (2021).

76. M. T. Richardson, M. S. Recouvreux, B. Y. Karlan, A. E. Walts, S. Orsulic, Ciliated cells in ovarian cancer decrease with increasing tumor grade and disease progression. *Cells* **11**, 4009 (2022).

77. Z. Hu, M. Artibani, A. Alsaadi, N. Wietek, M. Morotti, T. Shi, Z. Zhong, L. Santana Gonzalez, S. El-Sahhar, E. M. Carrami, G. Mallett, Y. Feng, K. Masuda, Y. Zheng, K. Chong, S. Damato, S. Dhar, L. Campo, R. Garruto Campanile, H. Soleymani Majd, V. Rai, D. Maldonado-Perez, S. Jones, V. Cerundolo, T. Sauka-Spengler, C. Yau, A. A. Ahmed, The repertoire of serous ovarian cancer non-genetic heterogeneity revealed by single-cell sequencing of normal fallopian tube epithelial cells. *Cancer Cell* **37**, 226–242.e7 (2020).

78. N. Karnezis, K. R. Cho, C. B. Gilks, C. L. Pearce, D. G. Huntsman, The disparate origins of ovarian cancers: Pathogenesis and prevention strategies. *Nat. Rev. Cancer* **17**, 65–74 (2017).

79. N. Yucer, R. Ahdoot, M. J. Workman, A. H. Laperle, M. S. Recouvreux, K. Kurowski, D. J. Naboulsi, V. Liang, Y. Qu, J. T. Plummer, S. A. Gayther, S. Orsulic, B. Y. Karlan, C. N. Svendsen, Human iPSC-derived fallopian tube organoids with BRCA1 mutation recapitulate early-stage carcinogenesis. *Cell Rep.* **37**, 110146 (2021).

80. R. J. Kurman, I.-M. Shih, The origin and pathogenesis of epithelial ovarian cancer: A proposed unifying theory. *Am. J. Surg. Pathol.* **34**, 433–443 (2010).

81. H. Chai, Q. Ren, Q. Fan, L. Ye, G. Du, H. Du, W. Xu, Y. Li, L. Zhang, Z. Cheng, PAX8 is a potential marker for the diagnosis of primary epithelial ovarian cancer. *Oncol. Lett.* **14**, 5871–5875 (2017).

82. B. K. Johnson, M. F. Majewski, H. J. Jang, R. A. Siwicki, M. Wegener, A. Semwal, J. Morrison, K. Gallik, M. Hagemann-Jensen, K. Foy, L. L. Rossell, E. J. Siegwald, D. W. Chesla, J. M. Teixeira, M. Adams, R. Drapkin, C. Esquibel, R. T. C. Sheridan, T. Wang, R. Sandberg, T. J. Triche Jr., H. Shen, Efficient profiling of total RNA in single cells with STORM-seq. bioRxiv 484332 [Preprint] (2025). https://doi.org/10.1101/2022.03.14.484332.

83. J. Crow, N. N. Amso, J. Lewin, R. W. Shaw, Physiology: Morphology and ultrastructure of fallopian tube epithelium at different stages of the menstrual cycle and menopause. *Hum. Reprod.* **9**, 2224–2233 (1994).

84. E. Novak, H. S. Everett, Cyclical and other variations in the tubal epithelium. *Am. J. Obstet. Gynecol.* **16**, 499–530 (1928).

85. S. Hou, Z. Li, X. Zheng, Y. Gao, J. Dong, Y. Ni, X. Wang, Y. Li, X. Ding, Z. Chang, S. Li, Y. Hu, X. Fan, Y. Hou, L. Wen, B. Liu, F. Tang, Y. Lan, Embryonic endothelial evolution towards first hematopoietic stem cells revealed by single-cell transcriptomic and functional analyses. *Cell Res.* **30**, 376–392 (2020).

86. W. Pu, H. Zhu, M. Zhang, M. Pikiolek, C. Ercan, J. Li, X. Huang, X. Han, Z. Zhang, Z. Lv, Y. Li, K. Liu, L. He, X. Liu, M. H. Heim, L. M. Terracciano, J. S. Tchorz, B. Zhou, Bipotent

transitional liver progenitor cells contribute to liver regeneration. *Nat. Genet.* **55**, 651–664 (2023).

87. M. N. Wicker, K.-U. Wagner, Cellular plasticity in mammary gland development and breast cancer. *Cancer* **15**, 5605 (2023).

88. H. O. D. Critchley, J. A. Maybin, G. M. Armstrong, A. R. W. Williams, Physiology of the endometrium and regulation of menstruation. *Physiol. Rev.* **100**, 1149–1179 (2020).

89. A. Salamonsen, J. C. Hutchison, C. E. Gargett, Cyclical endometrial repair and regeneration. *Development* **148**, dev199577 (2021).

90. B. Gellersen, J. Brosens, Cyclic AMP and progesterone receptor cross-talk in human endometrium: A decidualizing affair. *J. Endocrinol.* **178**, 357–372 (2003).

91. R. W. Noyes, A. T. Hertig, J. Rock, Dating the endometrial biopsy. *Am. J. Obstet. Gynecol.* **122**, 262–263 (1975).

92. C. A. Gray, F. F. Bartol, B. J. Tarleton, A. A. Wiley, G. A. Johnson, F. W. Bazer, T. E. Spencer, Developmental biology of uterine glands. *Biol. Reprod.* **65**, 1311–1323 (2001)

93. N. Tempest, A. Maclean, D. K. Hapangama, Endometrial stem cell markers: Current concepts and unresolved questions. *Int. J. Mol. Sci.* **19**, 3240 (2018).

94. M. Adler, A. R. Chavan, R. Medzhitov, Tissue biology: In search of a new paradigm. *Annu. Rev. Cell Dev. Biol.* **39**, 67–89 (2023).

95. S. Tulac, N. R. Nayak, L. C. Kao, M. Van Waes, J. Huang, S. Lobo, A. Germeyer, B. A. Lessey, R. N. Taylor, E. Suchanek, L. C. Giudice, Identification, characterization, and regulation of the canonical Wnt signaling pathway in human endometrium. *J. Clin. Endocrinol. Metabol.* **88**, 3860–3866 (2003).

96. W. Wang, F. Vilella, P. Alama, I. Moreno, M. Mignardi, A. Isakova, W. Pan, C. Simon, S. R. Quake, Single-cell transcriptomic atlas of the human endometrium during the menstrual cycle. *Nat. Med.* **26**, 1644–1653 (2020).

97. Y. Tan, W. F. Flynn, S. Sivajothi, D. Luo, S. B. Bozal, M. Davé, A. A. Luciano, P. Robson, D. E. Luciano, E. T. Courtois, Single-cell analysis of endometriosis reveals a coordinated transcriptional programme driving immunotolerance and angiogenesis across eutopic and ectopic tissues. *Nat. Cell Biol.* **24**, 1306–1318 (2022).

98. M. A. S. Fonseca, M. Haro, K. N. Wright, X. Lin, F. Abbasi, J. Sun, L. Hernandez, N. L. Orr, J. Hong, Y. Choi-Kuaea, H. M. Maluf, B. L. Balzer, A. Fishburn, R. Hickey, I. Cass, H. S. Goodridge, M. Truong, Y. Wang, M. D. Pisarska, H. Q. Dinh, A. EL-Naggar, D. G. Huntsman, M. S. Anglesio, M. T. Goodman, F. Medeiros, M. Siedhoff, K. Lawrenson, Single-cell transcriptomic analysis of endometriosis. *Nat. Genet.* **55**, 255–267 (2023).

99. E. M. Armstrong, I. A. R. More, D. McSeveney, W. R. Chatfield, Reappraisal of the ultrastructure of the human endometrial glandular cell. *BJOG* **80**, 446–460 (1973).

100. S. Fleming, D. N. Tweeddale, J. W. Roddick Jr., Ciliated endometrial cells. *Am. J. Obstet. Gynecol.* **102**, 186–191 (1968).

101. H. O. D. Critchley, R. M. Brenner, T. A. Henderson, K. Williams, N. R. Nayak, O. D. Slayden, M. R. Millar, P. T. K. Saunders, Estrogen receptor β, but not estrogen receptor β, is present in the vascular endothelium of the human and nonhuman primate endometrium. *J. Clin. Endocrinol. Metabol.* **86**, 1370–1378 (2001).

102. S. Matsuzaki, T. Fukaya, T. Suzuki, T. Murakami, H. Sasano, A. Yajima, Oestrogen receptor α and β mRNA expression in human endometrium throughout the menstrual cycle. *Mol. Hum. Reprod.* **5**, 559–564 (1999).

103. A. Ferenczy, G. Bertrand, M. M. Gelfand, Proliferation kinetics of human endometrium during the normal menstrual cycle. *Am. J. Obstet. Gynecol.* **133**, 859–867 (1979).

104. T. Kurita, P. Young, J. R. Brody, J. P. Lydon, B. W. O'Malley, G. R. Cunha, Stromal progesterone receptors mediate the inhibitory effects of progesterone on estrogen-induced uterine epithelial cell deoxyribonucleic acid synthesis. *Endocrinology* **139**, 4708–4713 (1998).

105. L. Garcia-Alonso, L.-F. Handfield, K. Roberts, K. Nikolakopoulou, R. C. Fernando, L. Gardner, B. Woodhams, A. Arutyunyan, K. Polanski, R. Hoo, C. Sancho-Serra, T. Li, K. Kwakwa, E. Tuck, V. Lorenzi, H. Massalha, M. Prete, V. Kleshchevnikov, A. Tarkowska, T. Porter, C. I. Mazzeo, S. Van Dongen, M. Dabrowska, V. Vaskivskyi, K. T. Mahbubani, J. Park, M. Jimenez-Linan, L. Campos, V. Yu. Kiselev, C. Lindskog, P. Ayuk, E. Prigmore, M. R. Stratton, K. Saeb-Parsy, A. Moffett, L. Moore, O. A. Bayraktar, S. A. Teichmann, M. Y. Turco, R. Vento-Tormo, Mapping the temporal and spatial dynamics of the human endometrium in vivo and in vitro. *Nat. Genet.* **53**, 1698–1711 (2021).

106. X. Huang, L. Wu, T. Pei, D. Liu, C. Liu, B. Luo, L. Xiao, Y. Li, R. Wang, Y. Ouyang, H. Zhu, W. Huang, Single-cell transcriptome analysis reveals endometrial immune microenvironment in minimal/mild endometriosis. *Clin. Exp. Immunol.* **212**, 285–295 (2023).

107. M. Marečková, L. Garcia-Alonso, M. Moullet, V. Lorenzi, R. Petryszak, C. Sancho-Serra, A. Oszlanczi, C. Icoresi Mazzeo, F. C. K. Wong, I. Kelava, S. Hoffman, M. Krassowski, K. Garbutt, K. Gaitskell, S. Yancheva, E. V. Woon, V. Male, I. Granne, K. Hellner, K. T. Mahbubani, K. Saeb-Parsy, M. Lotfollahi, E. Prigmore, J. Southcombe, R. A. Dragovic, C. M. Becker, K. T. Zondervan, R. Vento-Tormo, An integrated single-cell reference atlas of the human endometrium. *Nat. Genet.* **56**, 1925–1937 (2024).

108. Z.-Z. Lai, Y. Wang, W.-J. Zhou, Z. Liang, J.-W. Shi, H.-L. Yang, F. Xie, W.-D. Chen, R. Zhu, C. Zhang, J. Mei, J.-Y. Zhao, J.-F. Ye, T. Zhang, M.-Q. Li, Single-cell transcriptome profiling of the human endometrium of patients with recurrent implantation failure. *Theranostics* **12**, 6527–6547 (2022).

109. W.-J. Chae, A. L. M. Bothwell, Canonical and non-canonical Wnt signaling in immune cells. *Trends Immunol.* **39**, 830–847 (2018).

110. J. Von Maltzahn, C. F. Bentzinger, M. A. Rudnicki, Wnt7a-Fzd7 signalling directly activates the Akt/mTOR anabolic growth pathway in skeletal muscle. *Nat. Cell Biol.* **14**, 186–191 (2011).

111. B.-K. Koo, H. Clevers, Stem cells marked by the R-spondin receptor LGR5. *Gastroenterology* **147**, 289–302 (2014).

112. G. L. Rubin, A. J. Harrold, J. A. Mills, C. N. Falany, M. W. Coughtrie, Regulation of sulphotransferase expression in the endometrium during the menstrual cycle, by oral contraceptives and during early pregnancy. *Mol. Hum. Reprod.* **5**, 995–1002 (1999).

113. W. Fei, D. Kijima, M. Hashimoto, M. Hashimura, Y. Oguri, S. Kajita, T. Matsumoto, A. Yokoi, M. Saegusa, A functional role of LEFTY during progesterone therapy for endometrial carcinoma. *Cell Commun. Signal* **15**, 56 (2017).

114. A. Vilotić, M. Nacka-Aleksić, A. Pirković, Ž. Bojić-Trbojević, D. Dekanski, M. Jovanović Krivokuća, IL-6 and IL-8: An overview of their roles in healthy and pathological pregnancies. *Int. J. Mol. Sci.* **23**, 14574 (2022).

115. L. Marions, K. G. Danielsson, Expression of cyclo-oxygenase in human endometrium during the implantation period. *Mol. Hum. Reprod.* **5**, 961–965 (1999).

116. E. Jho, T. Zhang, C. Domon, C.-K. Joo, J.-N. Freund, F. Costantini, Wnt/β-catenin/Tcf signaling induces the transcription of Axin2, a negative regulator of the signaling pathway. *Mol. Cell. Biol.* **22**, 1172–1183 (2002).

117. H. Gehart, H. Clevers, Tales from the crypt: New insights into intestinal stem cells. *Nat. Rev. Gastroenterol. Hepatol.* **16**, 19–34 (2019).

118. E. J. Ostrin, D. R. Little, K. N. Gerner-Mauro, E. A. Sumner, R. Ríos-Corzo, E. Ambrosio, S. E. Holt, N. Forcioli-Conti, H. Akiyama, S. M. Hanash, S. Kimura, S. X. L. Huang, J. Chen, β-Catenin maintains lung epithelial progenitors after lung specification. *Development* **145**, dev160788 (2018).

119. E. C. Roberson, N. K. Tran, A. N. Godambe, H. Mark, M. Nguimtsop, T. Rust, E. Ung, L. J. Barker, R. D. Fitch, J. B. Wallingford, Hedgehog signaling is required for endometrial remodeling and myometrial homeostasis in the cycling mouse uterus. *iScience* **26**, 107993 (2023).

120. I. Kastrati, E. Canestrari, J. Frasor, PHLDA1 expression is controlled by an estrogen receptor-NFκB-miR-181 regulatory loop and is essential for formation of ER+ mammospheres. *Oncogene* **34**, 2309–2316 (2015).

121. D. M. Stone, M. Murone, S.-M. Luoh, W. Ye, M. P. Armanini, A. Gurney, H. Phillips, J. Brush, A. Goddard, F. J. D. Sauvage, A. Rosenthal, Characterization of the human Suppressor of fused, a negative regulator of the zinc-finger transcription factor Gli. *J. Cell Sci.* **112**, 4437–4448 (1999).

122. H. Kim, J. M. Kim, Y. Choi, Y. K. Shin, H. Lee, I. O. Seong, B. K. Kim, S. W. Chae, Y. Chung, S. Kim, Expression of Sonic hedgehog signaling molecules in normal, hyperplastic and carcinomatous endometrium. *Pathol. Int.* **59**, 279–287 (2009).

123. P. Li, X. Mao, Y. Ren, P. Liu, Epithelial cell polarity determinant CRB3 in cancer development. *Int. J. Biol. Sci.* **11**, 31–37 (2015).

124. I. Classen-Linke, S. Moss, K. Gröting, H. M. Beier, J. Alfer, C. A. Krusche, Mammaglobin 1: Not only a breast-specific and tumour-specific marker, but also a hormone-responsive endometrial protein. *Histopathology* **61**, 955–965 (2012).

125. O. Mani, M. Körner, M. T. Sorensen, K. Sejrsen, C. Wotzkow, C. E. Ontsouka, R. R. Friis, R. M. Bruckmaier, C. Albrecht, Expression, localization, and functional model of cholesterol transporters in lactating and nonlactating mammary tissues of murine, bovine, and human origin. *Am. J. Physiol. Regul. Integr. Comp. Physiol.* **299**, R642–R654 (2010).

126. I. Mylonas, U. Jeschke, C. Kunert-Keil, N. Shabani, D. Dian, I. Bauerfeind, C. Kuhn, M. S. Kupka, K. Friese, Glycodelin A is expressed differentially in normal human endometrial tissue

throughout the menstrual cycle as assessed by immunohistochemistry and in situ hybridization. *Fertil. Steril.* **86**, 1488–1497 (2006).

127. O. V. Burmenskaya, V. K. Bozhenko, V. Y. Smolnikova, E. A. Kalinina, I. E. Korneeva, A. E. Donnikov, E. P. Beyk, V. A. Naumov, N. V. Aleksandrova, P. I. Borovikov, D. Y. Trofimov, Transcription profile analysis of the endometrium revealed molecular markers of the personalized 'window of implantation' during in vitro fertilization. *Gynecol. Endocrinol.* **33**, 22–27 (2017).

128. J. Lipecki, A. E. Mitchell, J. Muter, E. S. Lucas, K. Makwana, K. Fishwick, J. Odendaal, A. Hawkes, P. Vrljicak, J. J. Brosens, S. Ott, EndoTime: Non-categorical timing estimates for luteal endometrium. *Hum. Reprod.* **37**, 747–761 (2022).

129. M. I. Hunter, K. M. Thies, W. Winuthayanon, Hormonal regulation of cilia in the female reproductive tract. *Curr. Opin. Endocr. Metab. Res.* **34**, 100503 (2024).

130. H. T. Ong, M. Sriram, H. H. Susapto, Y. Li, Y. Jiang, N. H. Voelcker, J. L. Young, A. W. Holle, R. Elnathan, The rise of mechanobiology for advanced cell engineering and manufacturing. *Adv. Mater.* **37**, e2501640 (2025).

131. M. D'Urso, N. A. Kurniawan, Mechanical and physical regulation of fibroblast-myofibroblast transition: From cellular mechanoresponse to tissue pathology. *Front. Bioeng. Biotechnol.* **8**, 609653 (2020).

132. D. Lindner, C. Zietsch, P. M. Becher, K. Schulze, H.-P. Schultheiss, C. Tschöpe, D. Westermann, Differential expression of matrix metalloproteases in human fibroblasts with different origins. *Biochem. Res. Int.* **2012**, 1–10 (2012).

133. V. S. LeBleu, E. G. Neilson, Origin and functional heterogeneity of fibroblasts. *FASEB J.* **34**, 3519–3536 (2020).

134. A. M. Sousa, T. Liu, O. Guevara, J. Stevens, B. L. Fanburg, M. Gaestel, D. Toksoz, U. S. Kayyali, Smooth muscle α-actin expression and myofibroblast differentiation by TGFβ are dependent upon MK2. *J. Cell. Biochem.* **100**, 1581–1592 (2006).

135. M. Gu, Y. Wang, Y. Yu, Ovarian fibrosis: Molecular mechanisms and potential therapeutic targets. *J. Ovarian Res.* **17**, 139 (2024).

136. D. A. Landry, E. Yakubovich, D. P. Cook, S. Fasih, J. Upham, B. C. Vanderhyden, Metformin prevents age-associated ovarian fibrosis by modulating the immune landscape in female mice. *Sci. Adv.* **8**, eabq1475 (2022).

137. M. Rodrigues, N. Kosaric, C. A. Bonham, G. C. Gurtner, Wound healing: A cellular perspective. *Physiol. Rev.* **99**, 665–706 (2019).

138. M. Zhang, Z. Chen, Y. Wang, H. Zhao, Y. Du, The role of cancer-associated fibroblasts in ovarian cancer. *Cancer* **14**, 2637 (2022).

139. H. Axemaker, S. Plesselova, K. Calar, M. Jorgensen, J. Wollman, P. De La Puente, Reprogramming of normal fibroblasts into ovarian cancer-associated fibroblasts via non-vesicular paracrine signaling induces an activated fibroblast phenotype. *Biochim. Biophys. Acta* **1871**, 119801 (2024).

140. L. Shen, J. Liu, A. Luo, S. Wang, The stromal microenvironment and ovarian aging: Mechanisms and therapeutic opportunities. *J. Ovarian Res.* **16**, 237 (2023).

141. X. Huang, C. He, X. Hua, A. Kan, Y. Mao, S. Sun, F. Duan, J. Wang, P. Huang, S. Li, Oxidative stress induces monocyte-to-myofibroblast transdifferentiation through p38 in pancreatic ductal adenocarcinoma. *Clin. Transl. Med.* **10**, e41 (2020).

142. X.-M. Meng, S. Wang, X.-R. Huang, C. Yang, J. Xiao, Y. Zhang, K.-F. To, D. J. Nikolic-Paterson, H.-Y. Lan, Inflammatory macrophages can transdifferentiate into myofibroblasts during renal fibrosis. *Cell Death Dis.* **7**, e2495-e2495 (2016).

143. G. Castellano, R. Franzin, A. Stasi, C. Divella, F. Sallustio, P. Pontrelli, G. Lucarelli, M. Battaglia, F. Staffieri, A. Crovace, G. Stallone, M. Seelen, M. R. Daha, G. Grandaliano, L. Gesualdo, Complement activation during ischemia/reperfusion injury induces pericyte-to-myofibroblast transdifferentiation regulating peritubular capillary lumen reduction through pERK signaling. *Front. Immunol.* **9**, 1002 (2018).

144. V. Sorokin, K. Vickneson, T. Kofidis, C. C. Woo, X. Y. Lin, R. Foo, C. M. Shanahan, Role of vascular smooth muscle cell plasticity and interactions in vessel wall inflammation. *Front. Immunol.* **11**, 599415 (2020).

145. E. A. McGlade, G. G. Herrera, K. K. Stephens, S. L. W. Olsen, S. Winuthayanon, J. Guner, S. C. Hewitt, K. S. Korach, F. J. DeMayo, J. P. Lydon, D. Monsivais, W. Winuthayanon, Cell-type specific analysis of physiological action of estrogen in mouse oviducts. *FASEB J.* **35**, e21563 (2021).

146. S. M. Briley, S. Jasti, J. M. McCracken, J. E. Hornick, B. Fegley, M. T. Pritchard, F. E. Duncan, Reproductive age-associated fibrosis in the stroma of the mammalian ovary. *Reproduction* **152**, 245–260 (2016).

147. F. Amargant, S. L. Manuel, Q. Tu, W. S. Parkes, F. Rivas, L. T. Zhou, J. E. Rowley, C. E. Villanueva, J. E. Hornick, G. S. Shekhawat, J. Wei, M. E. Pavone, A. R. Hall, M. T. Pritchard, F. E. Duncan, Ovarian stiffness increases with age in the mammalian ovary and depends on collagen and hyaluronan matrices. *Aging Cell* **19**, e13259 (2020).

148. T. Umehara, Y. E. Winstanley, E. Andreas, A. Morimoto, E. J. Williams, K. M. Smith, J. Carroll, M. A. Febbraio, M. Shimada, D. L. Russell, R. L. Robker, Female reproductive life span is extended by targeted removal of fibrotic collagen from the mouse ovary. *Sci. Adv.* **8**, eabn4564 (2022).

149. E. Geva, R. B. Jaffe, Role of vascular endothelial growth factor in ovarian physiology and pathology. *Fertil. Steril.* **74**, 429–438 (2000).

150. T. Israely, H. Dafni, D. Granot, N. Nevo, A. Tsafriri, M. Neeman, Vascular remodeling and angiogenesis in ectopic ovarian transplants: A crucial role of pericytes and vascular smooth muscle cells in maintenance of ovarian grafts. *Biol. Reprod.* **68**, 2055–2064 (2003).

151. J. D. Gordon, S. Mesiano, C. J. Zaloudek, R. B. Jaffe, Vascular endothelial growth factor localization in human ovary and fallopian tubes: Possible role in reproductive function and ovarian cyst formation. *J. Clin. Endocrinol. Metabol.* **81**, 353–359 (1996).

152. Y. Tang, C. Frisendahl, T. T. Piltonen, R. K. Arffman, P. G. Lalitkumar, K. Gemzell-Danielsson, Human endometrial pericytes: A comprehensive overview of their physiological functions and implications in uterine disorders. *Cells* **13**, 1510 (2024).

153. R. M. Hirschberg, J. Plendl, S. Kaessmeyer, Alpha smooth muscle actin in the cycling ovary—An immunohistochemical study. *Clin. Hemorheol. Microcirc.* **50**, 113–129 (2012).

154. T. Hino, R. Yanagimachi, Active peristaltic movements and fluid production of the mouse oviduct: Their roles in fluid and sperm transport and fertilization. *Biol. Reprod.* **101**, 40–49 (2019).

155. B. J. Doss, S. M. Wanek, S. M. Jacques, F. Qureshi, N. C. Ramirez, W. D. Lawrence, Ovarian smooth muscle metaplasia. *Int. J. Gynecol. Pathol.* **18**, 58–62 (1999).

156. M. Fukunaga, Smooth muscle metaplasia in ovarian endometriosis. *Histopathology* **36**, 348–352 (2000).

157. G. R. Guimarães, G. R. Maklouf, C. E. Teixeira, L. De Oliveira Santos, N. G. Tessarollo, N. E. De Toledo, A. F. Serain, C. A. De Lanna, M. A. Pretti, J. G. V. Da Cruz, M. Falchetti, M. M. Dimas, I. S. Filgueiras, O. Cabral-Marques, R. N. Ramos, F. C. De Macedo, F. R. Rodrigues, N. C. Bastos, J. L. Da Silva, E. Lummertz Da Rocha, C. B. P. Chaves, A. C. De Melo, P. M. M. Moraes-Vieira, M. A. Mori, M. Boroni, Single-cell resolution characterization of myeloid-derived cell states with implication in cancer outcome. *Nat. Commun.* **15**, 5694 (2024).

158. M. Kirshenbaum, R. Orvieto, Premature ovarian insufficiency (POI) and autoimmunity—An update appraisal. *J. Assist. Reprod. Genet.* **36**, 2207–2215 (2019).

159. S. M. Savukoski, H. Silvén, P. Pesonen, E. Pukkala, M. Gissler, E. Suvanto, M.-M. Ollila, M. Niinimäki, Excess of severe autoimmune diseases in women with premature ovarian insufficiency: A population-based study. *Hum. Reprod.* **39**, 2601–2607 (2024).

160. A. Converse, M. J. Perry, S. S. Dipali, J. V. V. Isola, E. B. Kelly, J. M. Varberg, M. B. Zelinski, M. B. Stout, M. T. Pritchard, F. E. Duncan, Multinucleated giant cells are hallmarks of

ovarian aging with unique immune and degradation-associated molecular signatures. *PLOS Biol.* **23**, e3003204 (2025).

161. S. H. L. George, A. Milea, P. A. Shaw, Proliferation in the normal FTE is a hallmark of the follicular phase, not BRCA mutation status. *Clin. Cancer Res.* **18**, 6199–6207 (2012).

162. L. Ardighieri, S. Lonardi, D. Moratto, F. Facchetti, I.-M. Shih, W. Vermi, R. J. Kurman, Characterization of the immune cell repertoire in the normal fallopian tube. *Int. J. Gynecol. Pathol.* **33**, 581–591 (2014).

163. T. Kader, J.-R. Lin, C. B. Hug, S. Coy, Y.-A. Chen, I. De Bruijn, N. Shih, E. Jung, R. J. Pelletier, M. Lopez Leon, G. Mingo, D. K. Omran, J. S. Lee, C. Yapp, B. A. Satravada, R. Kundra, Y. Xu, S. Chan, J. B. Tefft, J. L. Muhlich, S. H. Kim, S. M. Gysler, J. Agudo, J. R. Heath, N. Schultz, C. W. Drescher, P. K. Sorger, R. Drapkin, S. Santagata, Multimodal spatial profiling reveals immune suppression and microenvironment remodeling in fallopian tube precursors to high-grade serous ovarian carcinoma *Cancer Discov.* **15**, 1180–1202 (2025).

164. S. H. George, J. Greenaway, A. Milea, V. Clary, S. Shaw, M. Sharma, C. Virtanen, P. A. Shaw, Identification of abrogated pathways in fallopian tube epithelium from BRCA1 mutation carriers. *J. Pathol.* **225**, 106–117 (2011).

165. W. H. Kutteh, R. E. Blackwell, H. Gore, C. C. Kutteh, B. R. Carr, J. Mestecky, Secretory immune system of the female reproductive tract II. Local immune system in normal and infected fallopian tube. *Fertil. Steril.* **54**, 51–55 (1990).

166. J. Brand, M. Haro, X. Lin, B. J. Rimel, S. M. McGregor, K. Lawrenson, H. Q. Dinh, Fallopian tube single cell analysis reveals myeloid cell alterations in high-grade serous ovarian cancer. *iScience* **27**, 108990 (2024).

167. R. Vento-Tormo, M. Efremova, R. A. Botting, M. Y. Turco, M. Vento-Tormo, K. B. Meyer, J.-E. Park, E. Stephenson, K. Polański, A. Goncalves, L. Gardner, S. Holmqvist, J. Henriksson, A. Zou, A. M. Sharkey, B. Millar, B. Innes, L. Wood, A. Wilbrey-Clark, R. P. Payne, M. A. Ivarsson, S. Lisgo, A. Filby, D. H. Rowitch, J. N. Bulmer, G. J. Wright, M. J. T. Stubbington, M.

Haniffa, A. Moffett, S. A. Teichmann, Single-cell reconstruction of the early maternal-fetal interface in humans. *Nature* **563**, 347–353 (2018).

168. I. E. Lawrence Jr., H. W. Burden, The origin of the extrinsic adrenergic innervation to the rat ovary. *Anat. Rec.* **196**, 51–59 (1980).

169. J. McKey, C. Bunce, I. S. Batchvarov, D. M. Ornitz, B. Capel, Neural crest-derived neurons invade the ovary but not the testis during mouse gonad development. *Proc. Natl. Acad. Sci. U.S.A.* **116**, 5570–5575 (2019).

170. M. M. Brauer, P. G. Smith, Estrogen and female reproductive tract innervation: Cellular and molecular mechanisms of autonomic neuroplasticity. *Auton. Neurosci.* **187**, 1–17 (2015).

171. B. W. Herr II, J. Hardi, E. M. Quardokus, A. Bueckle, L. Chen, F. Wang, A. R. Caron, D. Osumi-Sutherland, M. A. Musen, K. Börner, Specimen, biological structure, and spatial ontologies in support of a Human Reference Atlas. *Sci. Data* **10**, 171 (2023).

172. E. M. Quardokus, D. C. Saunders, E. McDonough, J. W. Hickey, C. Werlein, C. Surrette, P. Rajbhandari, A. M. Casals, H. Tian, L. Lowery, E. K. Neumann, F. Björklund, T. V. Neelakantan, J. Croteau, A. E. Wiblin, J. Fisher, A. J. Livengood, K. G. Dowell, J. C. Silverstein, J. M. Spraggins, G. S. Pryhuber, G. Deutsch, F. Ginty, G. P. Nolan, S. Melov, D. Jonigk, M. A. Caldwell, I. S. Vlachos, W. Muller, N. Gehlenborg, B. R. Stockwell, E. Lundberg, M. P. Snyder, R. N. Germain, J. M. Camarillo, N. L. Kelleher, K. Börner, A. J. Radtke, Organ Mapping Antibody Panels: A community resource for standardized multiplexed tissue imaging. *Nat. Methods* **20**, 1174–1178 (2023).

173. K. Börner, S. A. Teichmann, E. M. Quardokus, J. C. Gee, K. Browne, D. Osumi-Sutherland, B. W. Herr, A. Bueckle, H. Paul, M. Haniffa, L. Jardine, A. Bernard, S.-L. Ding, J. A. Miller, S. Lin, M. K. Halushka, A. Boppana, T. A. Longacre, J. Hickey, Y. Lin, M. T. Valerius, Y. He, G. Pryhuber, X. Sun, M. Jorgensen, A. J. Radtke, C. Wasserfall, F. Ginty, J. Ho, J. Sunshine, R. T. Beuschel, M. Brusko, S. Lee, R. Malhotra, S. Jain, G. Weber, Anatomical structures, cell types and biomarkers of the Human Reference Atlas. *Nat. Cell Biol.* **23**, 1117–1128 (2021).

174. J. V. V. Isola, S. R. Ocañas, C. R. Hubbart, S. Ko, S. A. Mondal, J. D. Hense, H. N. C. Carter, A. Schneider, S. Kovats, J. Alberola-Ila, W. M. Freeman, M. B. Stout, A single-cell atlas of the aging mouse ovary. *Nat. Aging* **4**, 145–162 (2024).
